# Viral expression and molecular profiling in liver tissue versus microdissected hepatocytes in hepatitis B virus - associated hepatocellular carcinoma

**DOI:** 10.1186/s12967-014-0230-1

**Published:** 2014-08-21

**Authors:** Marta Melis, Giacomo Diaz, David E Kleiner, Fausto Zamboni, Juraj Kabat, Jinping Lai, Giulia Mogavero, Ashley Tice, Ronald E Engle, Steven Becker, Charles R Brown, Jeffrey C Hanson, Jaime Rodriguez-Canales, Michael Emmert-Buck, Sugantha Govindarajan, Michael Kew, Patrizia Farci

**Affiliations:** Hepatic Pathogenesis Section, Laboratory of Infectious Diseases, National Institute of Allergy and Infectious Diseases, National Institutes of Health, Bethesda, MD USA; Department of Biomedical Sciences, University of Cagliari, Cagliari, Italy; Laboratory of Pathology, National Cancer Institute, National Institutes of Health, Bethesda, MD USA; Liver Transplantation Center, Brotzu Hospital, Cagliari, Italy; Biological Imaging Facility/Research Technologies Branch, National Institute of Allergy and Infectious Disease, National Institutes of Health, Bethesda, MD USA; Laboratory of Molecular Microbiology, National Institute of Allergy and Infectious Diseases, National Institutes of Health, Bethesda, MD USA; Laser Capture Microdissection Core Facility, Laboratory of Pathology, National Cancer Institute, National Institutes of Health, Bethesda, MD USA; Department of Pathology, Rancho Los Amigos Hospital, University of Southern California, Downey, CA USA; Department of Medicine, Groote Schuur Hospital and University of Cape Town, Cape Town, South Africa

**Keywords:** Hepatocellular carcinoma, Hepatitis B virus, Gene expression profiling, Laser capture microdissection, Confocal microscopy

## Abstract

**Background:**

The molecular mechanisms whereby hepatitis B virus (HBV) induces hepatocellular carcinoma (HCC) remain elusive. We used genomic and molecular techniques to investigate host-virus interactions by studying multiple areas of the same liver from patients with HCC.

**Methods:**

We compared the gene signature of whole liver tissue (WLT) versus laser capture-microdissected (LCM) hepatocytes along with the intrahepatic expression of HBV. Gene expression profiling was performed on up to 17 WLT specimens obtained at various distances from the tumor center from individual livers of 11 patients with HCC and on selected LCM samples. HBV markers in liver and serum were determined by real-time polymerase chain reaction (PCR) and confocal immunofluorescence.

**Results:**

Analysis of 5 areas of the liver showed a sharp change in gene expression between the immediate perilesional area and tumor periphery that correlated with a significant decrease in the intrahepatic expression of HB surface antigen (HBsAg). The tumor was characterized by a large preponderance of down-regulated genes, mostly involved in the metabolism of lipids and fatty acids, glucose, amino acids and drugs, with down-regulation of pathways involved in the activation of PXR/RXR and PPARα/RXRα nuclear receptors, comprising PGC-1α and FOXO1, two key regulators critically involved not only in the metabolic functions of the liver but also in the life cycle of HBV, acting as essential transcription factors for viral gene expression. These findings were confirmed by gene expression of microdissected hepatocytes. Moreover, LCM of malignant hepatocytes also revealed up-regulation of unique genes associated with cancer and signaling pathways, including two novel HCC-associated cancer testis antigen genes, *NUF2* and *TTK*.

**Conclusions:**

Integrated gene expression profiling of whole liver tissue with that of microdissected hepatocytes demonstrated that HBV-associated HCC is characterized by a metabolism switch-off and by a significant reduction in HBsAg. LCM proved to be a critical tool to validate gene signatures associated with HCC and to identify genes that may play a role in hepatocarcinogenesis, opening new perspectives for the discovery of novel diagnostic markers and therapeutic targets.

**Electronic supplementary material:**

The online version of this article (doi:10.1186/s12967-014-0230-1) contains supplementary material, which is available to authorized users.

## Background

Hepatocellular carcinoma (HCC) is the third leading cause of cancer-related death worldwide [1]. In most patients, HCC arises in the setting of chronic liver disease of various etiologies, with cirrhosis being present in about 80% of the cases [2]. Chronic infection with hepatitis B virus (HBV) and hepatitis C virus (HCV) is responsible for over 80% of HCC cases worldwide. HBV was one of the first viruses for which a direct link with the development of HCC was demonstrated [3,4]. Thus, HCC was the first human cancer for which a viral cause was established, and the first to be shown to be preventable by universal vaccination [5]. Although the availability of an effective vaccine against HBV promises the eventual elimination of HBV-associated HCC, more than 350 million chronic carriers of HBV in the world are still at increased risk of developing cirrhosis and HCC, making HBV, along with tobacco, the most important environmental carcinogen [6]. Although the causal association between HBV and HCC has been well established, the molecular mechanisms of hepatocarcinogenesis remain elusive.

The advent of post-genomic technologies has provided tools to investigate the pathogenesis of liver cancer, making study of the simultaneous expression of mRNA of thousands of genes in a single array possible [7]. However, many studies of HCC derived from gene expression profiling have focused mainly on the host and little on the virus. HCC patients are often analyzed as a single group regardless of the etiologic factor involved, and the clinical, virologic and histologic features studied are often limited or missing. Moreover, there is limited information on the gene expression profiles of the surrounding non-tumorous tissue [8,9].

We took advantage of a unique collection of liver specimens from patients who underwent orthotopic liver transplantation (OLT) or partial hepatectomy for HBV-associated HCC to study simultaneously host and viral factors that contribute to hepatocarcinogenesis. To the best of our knowledge, this is the first study that reports the results of an extensive microarray analysis in which up to 17 specimens per patient were analyzed, including samples from the tumor, the neighboring tissue, and the most distant non-tumorous tissues, along with the intrahepatic expression of HBV. Moreover, because the liver contains heterogeneous cell populations, we investigated the gene expression profiles of malignant versus non-malignant hepatocytes isolated by laser capture microdissection (LCM). The combined study of gene expression profiling of whole liver tissue (WLT) with malignant and non-malignant microdissected hepatocytes, along with the analysis of the intrahepatic expression and distribution of HBV, provided new insights into the molecular programs involved in the pathogenesis of HBV-associated HCC, opening new perspectives for the identification of novel tumor markers, which are needed for the early detection of HCC and for the development of novel forms of therapy.

## Methods

### Patients

Multiple liver specimens were obtained from a cohort of 11 patients who underwent OLT or partial hepatectomy for HBV-associated HCC between 2004 and 2008 at the Liver Transplantation Center of the Brotzu Hospital in Cagliari, Italy. The patient characteristics are described in the Results section. The study was approved by the Office of Human Subjects Research of the National Institutes of Health, granted on the condition that all samples be made anonymous.

### Liver pathology

Liver biopsies were evaluated blindly by two expert hepatopathologists (D.K. and S.G.). For each liver biopsy specimen, activity grade and stage of fibrosis were established according to Ishak scoring system [10]. The grade of tumor differentiation was evaluated according to the Edmondson and Steiner grading system [11]. The HCC subtype was defined according to the classification of Yamashita et al. [12] based on EpCAM and alpha-fetoprotein (AFP) expression.

### Design of the study

#### Whole liver tissue

**Figure 1:**
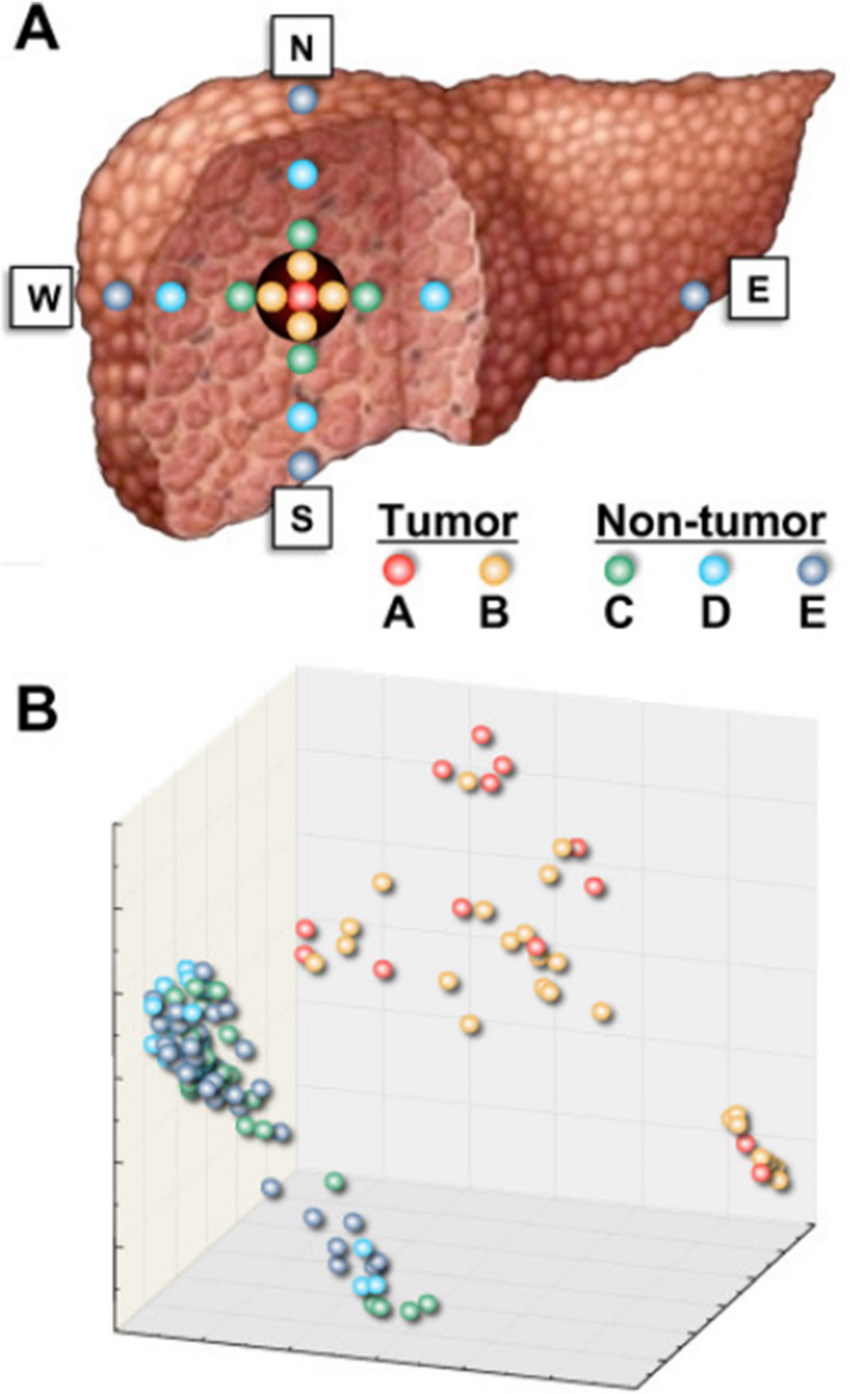
**Schematic representation of the study design illustrating the liver specimens analyzed for gene expression profiling and the multidimensional scaling plot. (A)** Different colors represent samples collected at different distances from HCC in the four directions (North, South, East and West). Red: HCC center (A area). Orange: HCC periphery (B area). Green: perilesional, non-tumorous tissue (C area). Turquoise: 2-3 cm from HCC (D area). Indigo: edge of the liver, 6-10 cm from HCC (E area). **(B)** Gene expression profiling of HBV-associated HCC. Multidimensional scaling plot showing the 3D projection of 120 liver specimens obtained at different distances from HCC from 11 patients. Each point of the scatterplot represents a liver specimen, and the distance between points is proportional to the overall dissimilarity of gene expression profiles.

Because of the complexity of HCC, our initial approach was to investigate, by microarray, the molecular heterogeneity within and outside the tumor by mapping the entire liver containing HCC. For this purpose, we analyzed up to 17 liver specimens for each of the 11 patients, taken in all 4 directions, termed north (N), south (S), east (E), and west (W) for simplicity, starting from the center of the tumor (Figure 1A). Specifically, the design included 5 biopsies from the tumor, one at the center (A) and 4 in the periphery of the tumor (B: N, S, E and W); 4 biopsies from the perilesional area (C: N, S, E and W); 4 biopsies taken 2-3 cm from the tumor (D: N, S, E and W); and 4 biopsies from the edges of the liver (E: N, S, E and W). In some cases, however, collection of non-tumorous liver specimens at all distances and directions from the center of the tumor was not possible because of the location of the tumor. On average, 11 liver specimens were collected from each patient from a total of 120 liver samples (39 from the tumor and 81 from the non-tumorous tissue) from the 11 patients. Each sample was divided into two pieces: one was snap-frozen for molecular studies and the other was formalin-fixed and paraffin-embedded (FFPE) for pathological examination. Importantly, when FFPE sections obtained from the tumor or the perilesional area showed a mixed population of tumor and non-tumor hepatocytes, the corresponding frozen liver specimens were excluded from microarray analysis. Of the 122 liver specimens analyzed, only two were excluded for the presence of a mixed population.

#### Laser capture microdissection

Because the liver contains a heterogeneous cell population, we also performed gene expression profiling on malignant and non-malignant hepatocytes, isolated by LCM, in 10 of the 11 patients previously investigated by microarray using WLT. For each patient, one biopsy from the center of the tumor (A) and one from the most distant area (E) were selected for LCM. We optimized the LCM method based on the procedure previously published by Erickson et al. [13]. Frozen liver sections were cut to a thickness of 8 μm and mounted on PEN membrane frame slides (Life Technologies, Carlsbad, CA, USA). Serial frozen sections were formalin fixed, H&E stained, and evaluated by a pathologist to determine areas of interest before dissection. To preserve RNA integrity, H&E staining was performed on ice and optimized for tissue visualization. Briefly, the excess of OCT was removed by submerging the slides in 70% ethanol (30 seconds), followed by molecular grade water (20 seconds), hematoxylin (30 seconds), molecular grade water (20 seconds), 1× bluing solution (20 seconds), 70% ethanol (30 seconds), eosin (3 seconds), 95% ethanol (30 seconds twice), 100% ethanol (30 seconds twice), and finally xylene (30 seconds). Xylene was allowed to completely evaporate from the slides in a chemical hood before use. LCM was performed using the Arcturus XT^TM^ Microdissection System (Life Technologies, Carlsbad, CA, USA). Areas of interest identified by a trained pathologist were selected on the freshly stained PEN membrane sections prior to placing the cap on the tissue. After cap placement, an infrared laser was used to melt the cap polymers to the areas of interest and a UV laser was used to cut these areas (Additional file 1: Figure S1). The cell populations were collected on the cap, removed from the PEN membrane slide, and incubated in the lysis buffer. RNA extraction was completed using the Arcturus PicoPure RNA Isolation Kit (Life Technologies, Carlsbad, CA, USA) according to the manufacturer’s recommendations. Approximately 2-7 square millimeters of tissue was cut from the section. The cell population recovered depended on the amount of artifact present, the cellular density and the size of the tissue section. In some cases, cells were collected from multiple sequential tissue slides. In order to minimize degradation of RNA from microdissected cells, LCM was completed within 60 minutes after staining and the RNA quality and integrity were assessed.

### Gene expression profiling

All liver specimens were analyzed by microarray using Affymetrix Human U133 Plus 2.0 arrays, which contain 54,675 transcripts representing approximately 27,000 unique human genes. Total RNA from WLT was extracted from frozen liver specimens as previously described [14] using TRIzol reagent (Invitrogen, Carlsbad, CA, USA) according to the manufacturer’s recommendations; total RNA from microdissected hepatocytes was extracted using the Arcturus PicoPure RNA Isolation Kit (Life Technologies, Carlsbad, CA, USA). The specimens used for RNA extraction and LCM were derived from the same frozen liver samples. Total RNA quality and integrity were assessed using the Agilent 2100 Bioanalyzer (Santa Clara, CA, USA). To maintain comparability between LCM and WLT, gene expression profiling was performed using the same technique as previously reported [14]. Total liver RNA (50 ng) obtained from whole liver tissue and microdissected hepatocytes was subjected to two successive rounds of amplification [15], and the resultant RNA was then subjected to biotin labeling, hybridization, staining, washing, and scanning procedures according to standard Affymetrix protocols.

### Serological and virological assays

Serologic markers of infection with hepatitis viruses were available in all patients at the time of OLT or partial hepatectomy. HBsAg, anti-HBs, anti-HBc, IgM anti-HBc, HBeAg, anti-HBe, antibody to HCV (anti-HCV), and antibody to human immunodeficiency virus (anti-HIV) were measured with a commercial enzyme immunoassay (Abbott Laboratories, North Chicago, IL, USA). Antibodies against hepatitis delta antigen (HDAg), IgG and IgM anti-HD, were measured using commercial enzyme immunoassays (Sorin Biomedica, Saluggia, Italy). Serum HBV DNA was quantified by a commercial assay (Amplicor, HBV Monitor test; Roche Diagnostics, Branchburg, NJ, USA). Serum HCV RNA was measured by a commercial assay (Cobas Amplicor HCV Monitor 2.0, Roche Diagnostics). Serum HDV RNA was evaluated by PCR as previously reported [16].

### Real-time polymerase chain reaction (PCR) assay for liver HBV DNA

HBV DNA in liver was quantified using a modification of a previously described method [17]. Briefly, 50 ng of total liver DNA were tested by real-time PCR. The primers/probe were located near the 5′ end of the S gene. Each 20 μL reaction contained 45 pmol of forward (5′- GGA CCC CTG CTC GTG TTA CA-3′) and reverse (3′- TTG AGA GAA GTC CAC CAC GAG TC-5′) primers, 12.5 pmol of non-fluorogenic-quenched-probe (6FAM- TGT TGA CAA GAA TCC TCA) and TaqMan® Fast Universal PCR Master Mix (Applied Biosystems, Foster City, CA). PCR was performed using an ABI PRISM® 7900HT Sequence Detection System (Applied Biosystems, Foster City, CA). Conditions included incubation at 95°C for 20 seconds followed by 45 PCR cycles of 1 second at 95°C and 20 seconds at 60°C. Viral titers were expressed as log_10_ IU per mL. The Acrometrix Optiquant (Acrometrix, Benicia, CA) HBV viral DNA panel was used to construct a standard curve from which IU quantities were determined. It consisted of 6 samples containing 2×10^2^ to 2×10^7^ IU/mL of HBV plus a negative control. The quantities of HBV DNA were calibrated using the WHO international standards (97/746), as previously described by Saldanha et al [18].

### Real-time quantitative PCR to validate cancer testis antigens identified by gene expression profiling

Real time reverse transcriptase quantitative polymerase chain reaction (RT QPCR) was used to validate four cancer testis antigens (CTA) found to be differentially expressed in microdissected hepatocytes between tumor and non-tumorous tissue. We used total RNA obtained from whole liver tissue because total RNA from microdissected hepatocytes had been entirely used for gene expression profiling. Reverse transcription was performed with a Bio-Rad iScript cDNA Synthesis Kit (Bio-Rad, Hercules, CA) and 25 ng of cDNA was used for each target in the SYBR Green QPCR method. We used commercially available primers for all four targets, as well as for the reference housekeeping gene (GAPDH) (PrimePCR, Bio-Rad, Hercules, CA). Reaction mixtures included SsoAdvanced Universal SYBR Green Supermix (Bio-Rad), target primer mix and samples, all used at the manufacturer’s recommended concentrations. PCR was carried out using an ABI PRISM® 7900HT Sequence Detection System (Applied Biosystems, Foster City, CA). Cycling conditions included 2 minutes at 95°C followed by 40 cycles of 5 seconds denaturation at 95°C, and 30 seconds annealing/extension at 60°C. Samples were tested in triplicate and the average results were expressed as –ΔΔCt [19], where ΔΔCt = (CtTarget - CtGAPDH )Tumor - (CtTarget - CtGAPDH)Non-Tumor.

### Immunohistochemistry, immunofluorescence, confocal microscopy, and image analysis

To define the HCC subtype, we investigated the expression of EPCAM and AFP in tumor liver sections [12]. Immunohistochemical staining of formalin fixed paraffin-embedded liver sections was performed using antibodies against EPCAM (Dako M0804, 1:200) and AFP (Dako A008, 1:2500). A BenchMark XT autostainer was used for antigen retrieval (if necessary), primary and secondary antibody incubation and detection according to the manufacturer’s instructions. Immunohistochemical staining of EPCAM was performed after antigen retrieval using treatment with Protease 1 (Ventana) for 4 minutes. Detection of AFP did not require antigen retrieval. To investigate the expression of the HBV markers, HBsAg and HBcAg, throughout the entire liver, we performed immunofluorescence and confocal microscopy in 10 of the 11 patients previously analyzed by microarray. We analyzed 4 liver specimens for each patient including two from the tumor (A and B) and 2 from the non-tumorous tissue, including the perilesional area (C) and the most distant non-tumorous tissue (E), along a single direction. Thus, a total of 20 tumor and 20 non-tumorous tissues were examined by immunofluorescence. As a negative control, we used FFPE liver sections obtained from a liver donor who showed normal liver histology and was negative for markers of infection with hepatitis viruses. To detect intrahepatic HBsAg and HBcAg, we used the method reported by Mensa et al. with some modifications [20]. Representative FFPE sections of 3 to 5 μm were heated overnight at 37°C, subsequently deparaffinized by xylene, and rehydrated in successive graded alcohol to distilled water. Antigen retrieval was performed with a pressure cooker (Dako, Glostrup, Denmark) by submerging sections in DIVA Decloaker Solution (Biocare Medical, LLC, Concord, CA, USA); the sections were then heated at 125°C at 21 psi for 4 minutes and 30 seconds. Non-specific binding sites were blocked with 10% goat serum for 30 minutes, and then slides were stained with mouse monoclonal anti-HBsAg (Dako) or rabbit polyclonal anti-HBcAg (Dako) for 1 hour at room temperature. After rinsing with 1× phosphate buffered saline (PBS), sections were incubated with a 1:400 dilution of the corresponding secondary antibody, Alexa Fluor 568 F(ab’) 2 fragment of goat anti-mouse IgG or Alexa Fluor 488 goat anti-rabbit IgG (both from Invitrogen, Carlsbad, CA, USA) for 30 minutes. After 3 washes with 1× PBS, samples were stained with DAPI (Invitrogen) and mounted with Fluorescence Mounting Medium (Dako). Samples were kept overnight in the dark before observation with a confocal microscope. Images were obtained using a Leica SP5 (Leica Microsystems, Exton, PA, USA) equipped with the 63× oil immersion objective NA 1.4. DAPI was excited using a 364 nm Enterprise II UV laser (Coherent, Santa Clara, CA, USA). To avoid emission crosstalk, sequential frame averaged scans were set up for each fluorophore. Data were deconvolved with Huygens Essential software (version 4.2.1p4, Scientific Volume Imaging BV, Hilversum, Netherlands). Sequential Z-sections of stained cells were acquired for 3-D reconstruction and iso-surface modeling with Imaris software (version 7.5.1, Bitplane AG, Zurich, Switzerland). The number of negative and positive cells was determined using the spot, surface and masking function of Imaris, and statistical data were calculated from multiple samples (at least 4 images for section) for each experiment.

### Statistical analysis

Microarray data were analyzed using BRB-Array Tools Version 4.2 [21], as previously reported [14]. Briefly, microarray raw data (.CEL files) were summarized and normalized by the RMA method. Transcripts showing minimal variation (less than 1.5-fold deviations from the median in more than 80% of the arrays) were excluded from the analysis. After filtering, only 21% of transcripts (11,377 from WLT and 11,224 from LCM microarrays) were eligible for subsequent analyses. Preliminary tests by Anova mixed model showed a prominent effect of the relative distance of the samples (A, B, C, D, and E) from the center of the tumor, whereas the direction of samples (N, S, E and W, relative to the center of the tumor) had no effect on gene expression. Thus, data from samples obtained in the 4 directions of the same liver area were averaged to increase the power of subsequent statistical analyses. To identify genes that were differentially expressed among the five liver areas, the areas were globally compared by a multivariate permutation F-test with a FDR <10% with 80% confidence level.^10^ Changes between specific liver areas were then detected by multiple pairwise t-tests at p = 0.001, using the subset of genes previously identified by the F-test. A second series of tests were performed to investigate the gene expression profile of microdissected hepatocytes by comparing microarray data of malignant and non-malignant cells isolated by LCM using a t-test with a FDR <10%. A parallel analysis was done to compare microarray data of WLT samples obtained from the center of the tumor (A) with those obtained from the most distant non-tumorous tissue (E). Gene expression fold changes were calculated as the ratio between the geometric means of tumor area (A) and non-tumorous tissue area (E), or malignant and non-malignant microdissected hepatocytes. Fold changes < 1 were expressed by the inverse ratio with a negative sign. Multidimensional scaling and hierarchical clustering were performed using individual samples from each patient. Multivariate analysis and heat maps were done using R software (R Development Core Team, http://www.r-project.org). Genes were organized into functional categories according to the Gene Ontology database (http://www.geneontology.org/). Pathway and network analysis was performed using Ingenuity Pathway Analysis (IPA) v 9.0 (http://www.ingenuity.com/). The association of genes to IPA canonical pathways was evaluated as the ratio between the number of genes present in the data set and the total number of genes that map to the same pathway. The Fisher’s exact test was also used to calculate the probability of such association. The complete microarray dataset is available at the Gene Expression Omnibus (http://www.ncbi.nlm.nih.gov/geo/; accession no.GSE55092).

## Results

### Characteristics of the patients

We studied 11 patients with HBV-associated HCC. Demographic, clinical, serological, and pathological features, including the grade and size of the tumor, are indicated in Table 1. All but one (91%) were males with a mean age (±SD) of 59.7 ± 7.7 years. Serum α-fetoprotein levels were normal in 8 subjects, with a mean (±SD) of 6.1 ± 2.7 ng/mL (normal range, <10.0 ng/mL) and abnormal in 2 patients with values of 20 and 292 ng/mL, respectively. The value was not available in one patient. All patients were positive for hepatitis B surface antigen (HBsAg), antibody to hepatitis B core antigen (anti-HBc) and antibody to hepatitis B e antigen (anti-HBe), and negative for hepatitis B e antigen (HBeAg) and antibody to HBsAg (anti-HBs). Patients were receiving antiviral treatment with nucleos(t)ide analogues prior to surgery. All, except one, were negative for antibodies to HCV and all were negative for serum HCV RNA. Another patient was found to be positive for IgG anti-HDV, but repeatedly negative for IgM anti-HDV and serum HDV RNA, whereas the remaining patients were negative for all markers of HDV infection. Of the 11 patients studied, 9 (82%) had underlying cirrhosis. The activity grade and stage of fibrosis of the surrounding non-tumorous tissue are reported in Table 1. The tumor size was less or equal to 3 cm in 8 patients (73%) and larger than 3 cm in the remaining three patients (Table 1). The grade of tumor differentiation was found to be G2 in seven patients, G3 in three patients, and G4 in the remaining patient (Table 1). To define the HCC subtypes of the 11 patients studied, we used the classification of Yamashita et al. [12] based on the expression of EPCAM and AFP, tested by immunohistochemistry on tumor sections. The results of the immunohistochemistry were correlated with the expression levels of EPCAM and AFP at the level of mRNA. We found that only one of the 11 patients with HBV-associated HCC in our series was positive for EPCAM but negative for AFP by immunohistochemistry. This patient was also the only one to show high EPCAM-mRNA levels within the tumor both in WLT and LCM. AFP was positive by immunohistochemistry in only one of the 11 HCC analyzed, but the level of AFP-mRNA in this patient was similar to the average level of the other patients. Thus, none of the 11 patients was positive for both EPCAM and AFP.

**Table 1:** **Base-line characteristics of the 11 patients with HBV-associated HCC**

**Characteristic**	
Age, *yr*	57.7 ± 7.7
Male, No. (*%*)	10 (90.9)
Alanine aminotransferase, *U/L* ^*a*^	36.18 ± 17.8
Aspartate aminotransferase, *U/L* ^*b*^	39.09 ± 17.0
Total bilirubin, *mg/dL*	0.88 ± 0.47
Albumin, *g/dL* ^*c*^	3.91 ± 0.57
γ-glutamyltransferase, *U/L*	93.9 ± 83.56
Prothombin time, *INR* ^*e*^	1.13 ± 0.14
Platelet count, *per mm* ^*3*^	153,81 ± 93,73
α-fetoprotein, *ng/mL* ^*d*^	
Normal, No.	8
Abnormal, No.	2
Non-tumorous tissue	
Activity Grade	5.75 ± 3.06
Fibrosis Stage	5.1 ± 1.56
F5/F6, No.	9
Tumor Grade^f^	
G2, No.	7
G3, No.	3
G4, No.	1
Tumor size	
≥2 cm, No.	4
≥2 and ≤ 3 cm, No.	4
>3 cm, No.	3
HBsAg-positive, No.	11
HBeAg-positive, No.	0
Anti-HBc, No.	11
Anti-HBe, No.	11
IgG anti-HDAg positive, No.	1
IgM anti-HDAg, No.	0
Serum HDV RNA positive, No.	0
Anti-HCV positive, No.	1
Serum HCV RNA positive, No.	0

NOTE. Plus-minus values are means ± SD. To convert values for total bilirubin to micromoles per liter, multiply by 17.1. HBsAg, hepatitis B surface antigen; HBeAg, hepatitis B e antigen; HDAg, hepatitis delta antigen; HBV, hepatitis B virus; and HDV, hepatitis D virus.

^a^Normal range, ≤43 U per liter.

^b^Normal range, ≤42 U per liter.

^c^Normal range, ≥3.6 - ≤5.0.

^d^Normal range, <10.0 ng/mL. Data were not available from one patient.

^e^Normal range, 0.80-1.20 international normalized ratio (INR).

^f^Tumors were graded using the Edmondson-Steiner criteria.

### Differential gene expression between tumor and non-tumorous areas

**Figure 2:**
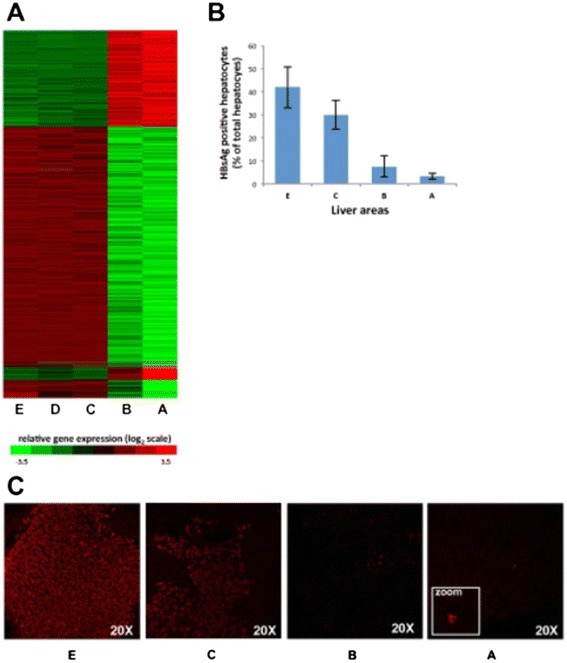
**Gene expression profiling and intrahepatic expression of HBV antigens in HBV-associated HCC.** **(A)** Heat map of 1,486 genes differentially expressed in different areas of livers containing HCC. These data were obtained from whole liver tissue. A denotes tumor center; B tumor periphery; C the perilesional non-tumorous area, outside the tumor margin; D a distance of 2-3 cm from the margin of the tumor; E the most distant non-tumorous area, the edge of the liver. Data from the center of the tumor (A) represent individual samples, whereas data from each of the remaining liver areas (from B to E) represent the average of multiple specimens obtained in the 4 directions (N, S, E, and W). Each cell represents the expression of a particular gene (rows) of a particular liver area (columns). The color in each cell reflects the level of expression of the corresponding gene in the corresponding area, relative to its mean level of expression in the entire set of 120 samples. Ratios were log2-transformed and row-wise standardized. Up-regulated genes are shown in shades of red, down-regulated genes in shades of green. **(B)** Frequency of positive hepatocytes for HBsAg immunostaining within and outside the tumor of 11 patients. Data are represented as mean SE. Liver samples for HBsAg staining from areas B and C were available in 8 patients **(C)**. Sections representing different liver areas show a significant decrease in number of HBsAg-positive hepatocytes between the perilesional non-tumorous area (C) and the periphery of the tumor margin (B), which further decreased in the center of the tumor (A). HBsAg positivity was almost diffuse in the most distant non-tumorous area E, with a gradual decrease in area C, whereas scattered or isolated positive hepatocytes (*Inset*) were mostly found in areas B and A, respectively (Original magnification 20X).

To investigate the molecular changes that occur within a liver containing HCC, we performed gene expression profiling of multiple liver specimens of the same liver, both within and outside the tumor, from 11 patients with HBV-associated HCC. An unsupervised multidimensional scaling of all 120 specimens obtained from the five areas of HCC-containing livers disclosed 2 distinct clusters corresponding to tumor areas (A and B) and non-tumorous areas (C, D and E) (Figure 1B). To identify the genes that were mostly responsible for such marked difference between the tumor and non-tumorous tissue, microarray data of the five different areas of HCC-containing livers were compared by a multivariate permutation F-test [false discovery rate (FDR) <10%]. The analysis identified 1,486 genes differentially expressed, with the majority (two-thirds) of genes down-regulated in the tumor (Additional file 2: Table S1). The F-test confirmed the overall difference between tumor and non-tumorous tissue, as all genes that were up-regulated in the tumor (A and B) areas were down-regulated in all non-tumorous areas (C, D and E); conversely all genes down-regulated in the tumor areas were up-regulated in all non-tumorous areas. A sharp demarcation between tumor and non-tumorous tissues is also shown by the heat map obtained from WLT gene expression data (Figure 2A).

### Correlation between gene expression and intrahepatic expression of HBsAg, HBcAg and HBV DNA in tumor and non-tumorous areas and in serum

**Figure 3:**
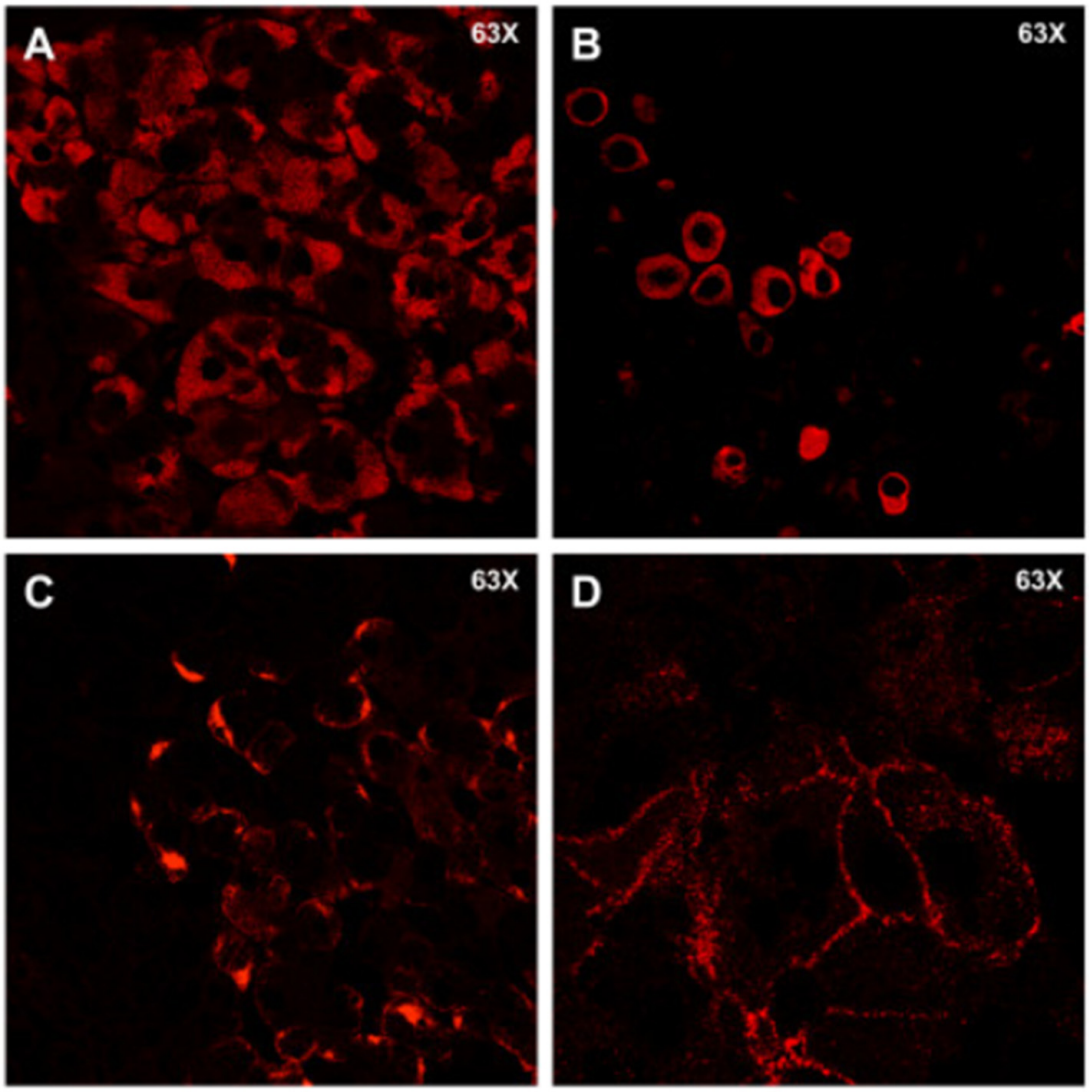
**Patterns of HBsAg expression detected by immunofluorescence in the liver of patients with HBV-associated HCC.** The images illustrate four representative patterns of HBsAg positivity: cytoplasmic diffuse, extended to vast areas of the liver section, found in non-tumorous areas **(A)** cytoplasmic scattered, found in isolated hepatocytes both in tumoral and non-tumorous areas **(B)** cytoplasmic spotty, found in limited areas of the hepatocyte cytoplasm both in tumoral and non-tumorous areas **(C)** membranous, limited to the plasma membrane of hepatocytes within the tumoral areas **(D)**. (Original magnification 63×).

**Figure 4:**
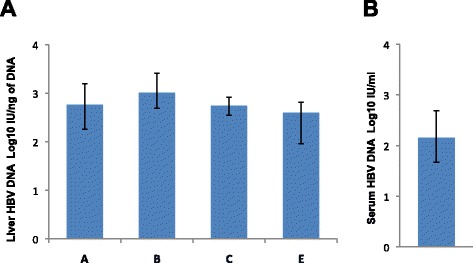
**HBV DNA levels in liver and serum of patients with HBV-associated HCC. (A)** HBV DNA levels in different areas of the liver, within the center **(A)** and the periphery of the tumor **(B)** and progressively distal to the tumor (C and E), as measured by real-time PCR in 11 patients with HBV-associated HCC. **(B)** HBV DNA levels in serum of patients with HBV-associated HCC. Quantities are expressed as Log_10_ IU/ng (liver) or Log_10_ IU/mL (serum) as compared to an international HBV DNA standard. The levels of HBV DNA in the different areas of the liver and in serum are shown as median ± interquartile range. Liver samples for HBV DNA quantification from areas B and C were available in only 8 patients.

Access to a unique series of liver specimens from 11 patients with HCC provided us with the opportunity to study the intrahepatic expression of HBsAg and HBcAg by confocal immunofluorescence in different areas of the liver, including the center and the periphery of the tumor, the perilesional area outside the tumor and the most distant area from the tumor along a single direction. This analysis allowed us to evaluate the relationship between gene expression and the intrahepatic expression of HBV. HBsAg immunostaining was positive in only 6 of 11 tumor tissues, but in all non-tumorous specimens. When we measured the number of hepatocytes positive for HBsAg in the different areas of the liver, we observed a significant drop in the number of positive hepatocytes from the perilesional non-tumorous area (C) to the periphery of the tumor area (B) (Figure 2B and C), which mirrored the sharp demarcation observed in the gene expression profile (Figure 2A). Such a decrease in HBsAg positivity continued until the center of the tumor, which was characterized by the lowest levels of HBsAg expression (Figure 2B). Conversely, no significant differences were observed in the number of HBsAg-positive hepatocytes among the non-tumorous areas. In addition to quantitative differences, HBsAg exhibited different expression patterns in tumor and non-tumorous areas (Figure 3). Whereas non-tumorous liver tissues were characterized by a diffuse, scattered or spotty cytoplasmic pattern (Figure 3A, B and C), tumor tissues showed a scattered or membranous pattern, the latter being localized on the plasma membrane uniquely on malignant hepatocytes (Figure 3D). In contrast, HBcAg was negative both in tumor and non-tumorous tissues, and was positive only in a few cells in one non-tumorous tissue. Quantification of HBV DNA in liver by real-time PCR showed low levels of HBV DNA, without differences between tumor and non-tumorous areas (Figure 4). These findings were consistent with the low levels of HBV DNA detected in serum (Figure 4).

### Genes differentially expressed in malignant versus non-malignant hepatocytes (LCM) and in tumor versus non-tumorous tissue (WLT)

**Figure 5:**
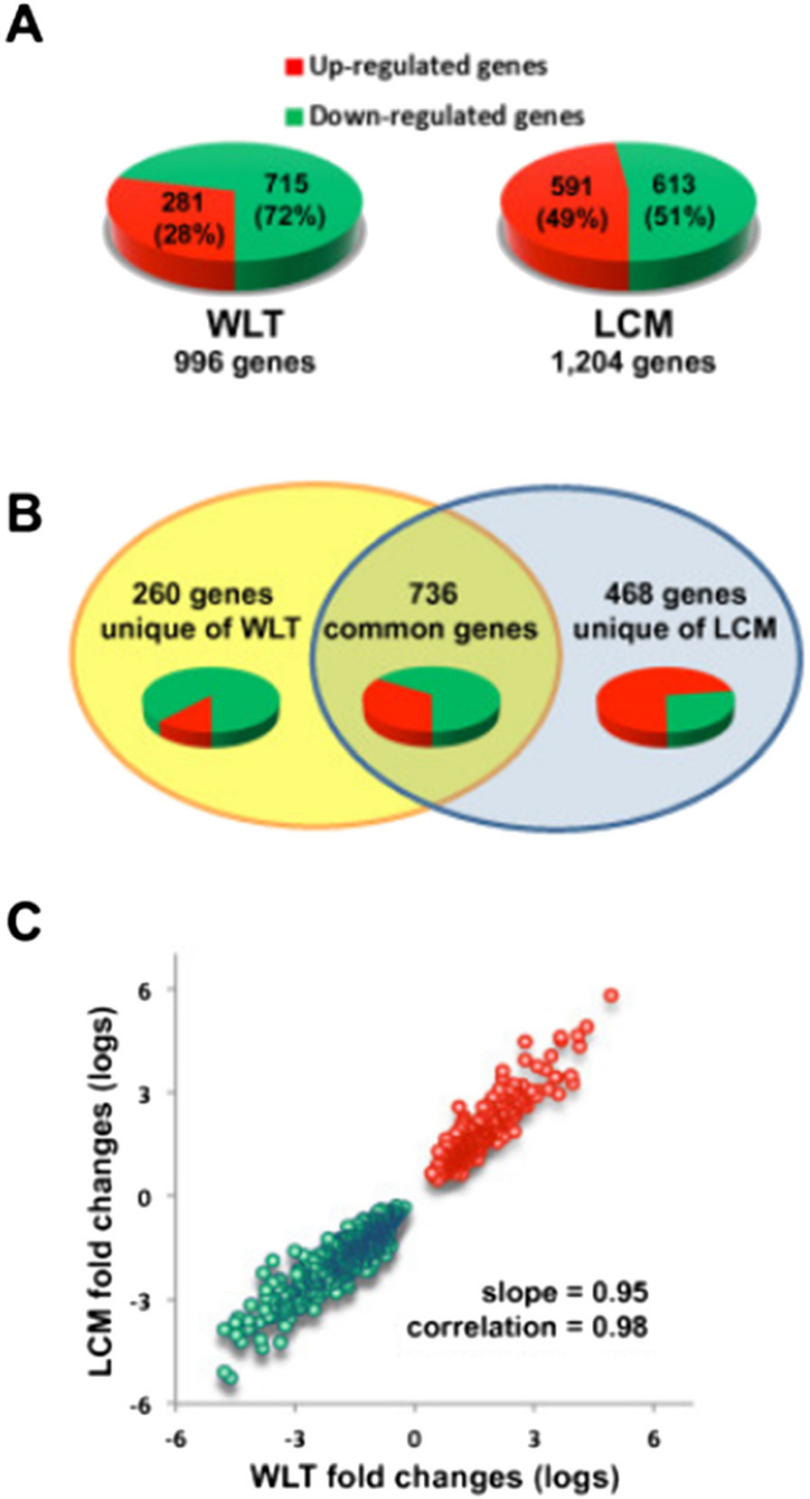
**Differentially expressed genes in whole liver tissue (WLT) and laser capture microdissected hepatocytes (LCM). (A)** The pie diagrams show the percentage of the differentially expressed genes in WLT (n = 996) and LCM (n -= 1,204) obtained by a multivariate permutation t-test with a FDR < 10%. In both techniques the two sets of genes were obtained by comparing the center of the tumor with the most distant non-tumorous area. Up-regulated genes are depicted in red, whereas down-regulated genes are in green. **(B)** Venn diagram showing the common and unique genes among the sets of genes detected by t-test (FDR < 10%) in WLT and LCM samples. **(C)** Correlation of log-transformed fold changes of the 736 common genes identified by LCM and WLT. LCM fold changes were calculated as the ratio between gene expression of malignant and non-malignant hepatocytes. WLT fold changes were calculated as the ratio between gene expression of tumor and non-tumorous tissue areas (A and E, respectively). Red and green points represent up-regulated and down-regulated genes, respectively.

To identify genes that were differentially expressed in malignant hepatocytes, a multivariate permutation t-test with a FDR <10% was performed between microdissected malignant hepatocytes isolated from the central area of the tumor (A) and microdissected hepatocytes isolated from the most peripheral non-tumorous area (E) by LCM. This analysis identified a total of 1,204 genes differentially expressed, 591 (49%) of which were found to be up-regulated and 613 (51%) down-regulated (Figure 5A and Additional file 3: Table S2). The same t-test was then applied to identify genes differentially expressed at the tissue level (WLT) by comparing the center of the tumor area (A) with the most distant non-tumorous area (E). Statistical analysis identified 996 genes differentially expressed in the tumor tissue, with 281 (28%) genes up-regulated and 715 (72%) down-regulated (Figure 5A and Additional file 4: Table S3). The marked difference between the percentages of up- and down-regulated genes detected by LCM and WLT (49/51% vs 28/72%, respectively) was statistically significant (p < 0.0001 by Yates’ chi-square). This led us to compare the sets of genes in order to identify: 1) genes differentially expressed both in LCM and WLT samples; 2) genes differentially expressed only in WLT; and 3) genes differentially expressed only in LCM samples (Figure 5B).

#### Genes differentially expressed both in malignant hepatocytes and tumor tissue (LCM-WLT-common genes)

**Figure 6:**
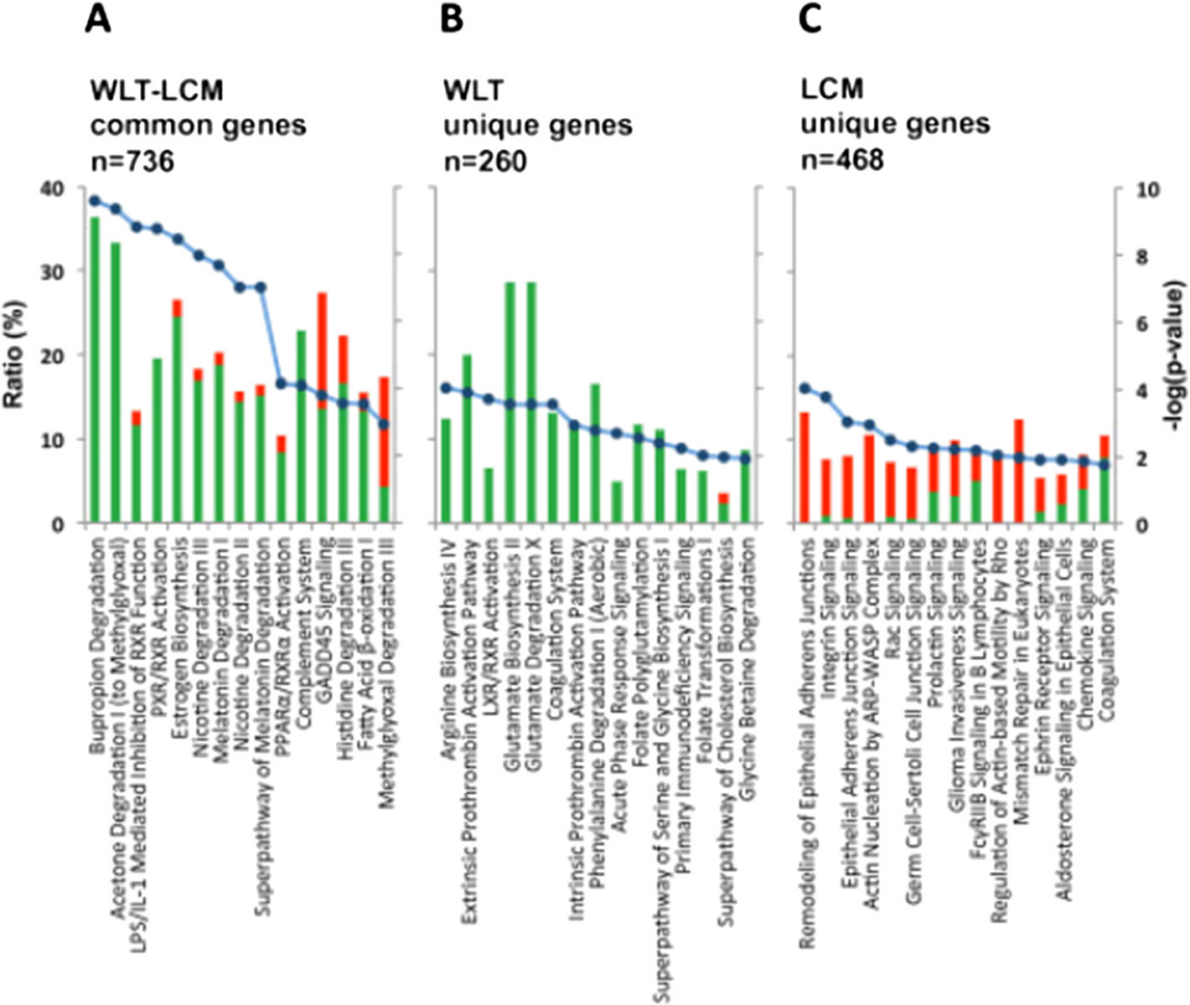
**Top-scored canonical pathways of the three gene subsets obtained from the cross analysis comparing WLT (n = 996) and LCM (n = 1,204) gene lists: (A) LCM-WLT-common genes (n = 736); (B) WLT-unique genes (n = 260); (C) LCM-unique genes (n = 468).** The top-scored canonical pathways of LCM-WLT-common and WLT-unique genes show a striking majority of down-regulated pathways involved in metabolic processes (A and B). In contrast, the LCM-unique genes (C) are characterized by a predominance of up-regulated pathways involving cell remodeling and cell motility. Expression of actin/cytoskeleton-, integrins-, and adherens junctions-related pathways suggests cell remodeling activity, whereas Rac- and Rho-associated pathways are indicative of increased cell motility of malignant hepatocytes. Columns (left axis) represent the percentage ratio of the number of genes in a given pathway divided by the total number of genes that make up that pathway. Green and red columns indicate down- and up-regulated genes, respectively. Blue points (right axis) display the -log of p-value calculated by Fisher’s exact test right-tailed. (Ingenuity Pathway Analysis, www.ingenuity.com). For each dataset, pathways are ordered from the most significant p-values.

Microdissected malignant hepatocytes and whole tumor tissue shared 736 differentially expressed genes (Figure 5B), accounting for 61% of LCM genes and 74% of WLT genes (Additional file 5: Table S4). Bivariate analyses of fold changes showed a close correlation (r = 0.98) and an almost unitary slope (b = 0.95), which attested to a consistent agreement of gene expressions observed in malignant hepatocytes and in tumor tissue (Figure 5C). Among the 736 genes in common, 251 (34%) were up-regulated, whereas the vast majority were down-regulated (485, 66%). The high prevalence of down-regulated genes was also reflected by the analysis of canonical pathways (Figure 6A), which showed an extensive down-regulation of pathways involved in the metabolism and degradation of various substrates (bupropion, acetone, nicotine, melatonin, histidine and methylglyoxal), activation of PXR/RXR and PPARα/RXRα nuclear receptors (NR), estrogen biosynthesis, complement system and fatty acid β-oxidation I. Consistent with down-regulation of the metabolism-related canonical pathways, the most represented down-regulated genes were members of the cytochrome family (*CYP2E1, CYP2C9, CYP2A13, CYP2A6, CYP2A7, CYP3A4, CYP3A5, CYP3A7, CYP1A2, CYP2B6, CYP2C8, CYP2C19, CYP4A11, CYP4A22*) and genes involved in the metabolism of lipids and hormones (*AKR1D1, ESR1, HMGCS2, LIPC, ABCG5, CETP, PPAP2B, RDH16, SLC27A2, LCAT, APOA5, ACAA2*), carbohydrates (*GYS2, PCK1, FBP1, G6PC, ALDOB*), and amino acids (*HAO2, HAL, ASPA, IDO2*) (Additional file 5: Table S4). Also, several genes related to hepatic synthesis, including complement system and inflammation (*C9, FCN3, FCN2, CXCL14*), as well as immune response genes (*CLEC1B, CLEC4G, CLEC4M*) were down-regulated. Within NR-associated canonical pathways, the key transcriptional regulators *PPARGC-1α/ PGC-1α* (peroxisome proliferator-activated receptor gamma coactivator-1 alpha) and *FOXO1* (forkhead box O1) were down-regulated. Moreover, most of down-regulated metabolism-related genes were downstream of NRs, thus confirming the major regulatory role of NRs in HBV-associated HCC. Consistent with increased cell growth, several negative regulators of cell proliferation (*GADD45A, GADD45B, GADD45G*) were down-regulated (Additional file 5: Table S4). Another gene that was considerably down-regulated within the tumor was sodium dependent taurocolic cotransporting polypeptide (*NTCP/ SLC10A1*), which was recently identified as a receptor for human hepatitis B virus [22]. Among up-regulated genes, the most represented were those involved in cell cycle and proliferation (*AKR1B10, ANLN, TOP2A, IGF2BP3, ASPM, CDK1, ECT2, CCNB1, NDC80, PRC1, NEK2, CENPF*) (Additional file 5: Table S4).

#### Genes differentially expressed in tumor tissue but not in microdissected malignant hepatocytes (WLT-unique genes)

The set of genes differentially expressed in tumor tissue but not in microdissected malignant hepatocytes comprised 260 genes, the majority of which (88.5%) were down-regulated (Figure 5B and Additional file 6: Table S5). This was consistent with the analysis of the canonical pathways, which showed a dramatic down-regulation of pathways involved in amino acid biosynthesis and degradation, activation of LXR/RXR NR, extrinsic and intrinsic prothrombin activation pathway, coagulation system, and acute phase response signaling. In line with these findings, there was an overriding enrichment of metabolism-related genes (*ADH1A, HPGD, HSD17B2, BBOX1, AGXT2L1*) followed by genes involved in detoxification (*AGXT*), inflammation (*C7, HPGD, HRG, HPX, IL13RA2, PTGS2*) and immune response (*IGHM and IGL@)* (Figure 6B). Only a minority of genes were up-regulated (11.5%), and among them those involved in cell-proliferation were the most up-regulated (*SFN* and *MKI67*).

#### Genes differentially expressed in microdissected malignant hepatocytes but not in tumor tissue (LCM-unique genes)

Cross-comparison of LCM and WLT data identified 468 genes differentially expressed by microdissected malignant hepatocytes but not in tumor tissue. Unlike the results obtained with WLT, we observed a predominance of up-regulated genes (73%) (Figure 5B). Moreover, the top-scored pathways were not associated with metabolism but rather with signal transduction cascades mediated chiefly by integrins and cadherins (integrin signaling, adherens junction remodeling and signaling, actin nucleation by ARP-WASP complex and Rac signaling) (Figure 6C and Additional file 7: Table S6). These pathways control a range of cell activities such as cell adhesion, motility, contractility and proliferation by means of cytoskeletal interactions and rearrangements, as well as activation of transcription factors. However, these pathways included only 10% (45 out of 468) LCM-unique genes. On the other hand, the analysis of single gene functions was more specifically related to the nature of malignant hepatocytes, with more than 50% of the 468 LCM-unique genes associated with cancer, 29% with cell proliferation, 22% with apoptosis and 12% with cell survival. Importantly, among the most up-regulated genes (i.e., genes with fold changes > 4) four were cancer testis antigen (CTA) genes (*MAGEA3, NUF2, CEP55* and *TTK*) (Additional file 7: Table S6). Expression of the 4 CTA genes was also tested by real-time PCR. Interestingly, the gene expression profiles of these 4 genes were very similar when two independent methods were used, showing a good concordance between signal intensities measured by gene expression profiling and gene expression measured by real-time PCR (Additional file 8: Figure S2 and Additional file 9: Figure S3). Recently, Nault et al [23], reported a 5-gene score in HCC, comprising *HN1, RAN, RAMP3, KRT19, and TAF9,* which was associated with patient survival after liver resection. We have analyzed the 5-gene score in our data set to see whether these genes were differently expressed in WLT and LCM. Interestingly, we found that only 2 out of the 5 genes (*HN1* and *RAN*) were differentially expressed in our cohort of patients but only by LCM, suggesting that these genes are specifically expressed by malignant hepatocytes. The lack of information on the long-term clinical outcome and the limited number of patients included in this study did not allow us to assess a statistical correlation between gene expression and survival.

## Discussion

Our comparative analysis of multiple liver specimens sampled at various distances from the center of the tumor allowed us to demonstrate that the liver containing HBV-associated HCC is characterized by a sharp change in gene expression at the immediate perilesional area, within millimeters of the tumor margin. Moreover, we documented that this change is highly specific, as all genes down-regulated within the tumor were up-regulated in all non-tumorous liver areas and, conversely, all genes up-regulated in the tumor were down-regulated in all non-tumorous areas.

To the best of our knowledge, this is the first study that integrated gene expression profiling of whole liver tissue with that of microdissected hepatocytes from the same cohort of patients with HBV-associated HCC. Accruing evidence has documented the importance of the LCM technique to study isolated cell populations in tumors that arise in histologically heterogeneous tissues [24]. The comparison of WLT and LCM data made it possible to distinguish three subsets of genes: genes detected in both WLT and LCM samples, genes detected only in LCM samples, and genes detected only in WLT samples. Interestingly, genes detected in both WLT and LCM samples showed comparable fold changes and the vast majority were down-regulated like most of WLT-unique genes. Conversely, most LCM-unique genes were up-regulated, indicating that malignant hepatocytes and whole tumor tissue diverge not only in the nature of genes differentially expressed but also in the overall direction of the change. Such a discrepancy underlines the importance of LCM to collect specific information on the gene regulation of malignant hepatocytes.

Among genes detected by both WLT and LCM samples, an overwhelming number of genes were involved in the metabolism of lipids and fatty acids, glucose, amino acids and drugs and were down-regulated. In agreement with our observations, previous studies showed down-regulation of metabolism-related genes in HBV-related HCC [25], a feature that is preferentially linked to HBV- rather than to HCV-associated HCC [26,27]. The reasons for this dramatic down-regulation of metabolism-associated genes are presently unknown, although some studies have suggested that this phenomenon is part of the de-differentiation program of liver tumor cells, which is particularly evident in HCC associated with HBV infection [28]. Consistent with this hypothesis, Nagata *et al.* [29] found a remarkable down-regulation of cytochrome-associated genes in fetal human liver compared to adult liver, and explained this finding with the absence of hepatocyte-specific function in fetal livers.

Two of the most overexpressed genes in HBV-associated HCC, found both by LCM and WLT, that may be of particular interest are *AKR1B10* and *IGF2BP3. AKR1B10* has recently been associated with several tumors including, but not limited to, pancreatic carcinoma [30], breast cancer [31], and papillary renal carcinoma [32]. However, studies in HCC are limited [33]. Although its function is still largely unknown, *AKR1B10* was shown to deplete cells of retinoic acid, which controls cell proliferation [34]. In support of a possible role of retinoic acid depletion, we also detected down-regulation of *RDH16*, the key enzyme responsible for retinoic acid synthesis. Regarding *IGF2BP3*, whose function is even less well established, its depletion has been correlated with a decrease in cell motility, invasion and transendothelial migration [35]. In a recent study published by our group [36], *AKR1B10* and *IGF2BP3* were found to be highly up-regulated in the liver of patients with HBV-associated acute liver failure and evidence of liver regeneration, emphasizing the need to further dissect the relationship between liver regeneration and liver cancer.

An important finding, which further highlights the value of LCM, is the up-regulation of four CTA genes (MAGEA3, NUF2, CEP55, and TTK) that we detected in microdissected malignant hepatocytes, and confirmed by real-time PCR. MAGEA3, one of the first CTAs associated with HCC, is a member of the MAGE gene family and a candidate for specific HCC immunotherapy [37]. It has been proposed that MAGEA3 may enhance the ubiquitin ligase activity of TRIM28 and stimulate p53/TP53 ubiquitination by TRIM28 [38]. The other three CTAs are directly involved, at various levels, in the mitotic machinery. CEP55 has previously been associated with HCC [39], whereas, to our knowledge, NUF2 and TTK are novel HCC-associated CTAs. Members of the CTA family have been suggested as potential targets for cancer immunotherapy because, unlike most auto-antigens, they are highly immunogenic, even in autologous cancer-bearing patients.

One of the major goals of our study was to investigate the relationship between gene expression and viral biomarkers in the liver. The sharp change in gene expression that we documented between the perilesional area and the periphery of the tumor was mirrored by a significant decrease in HBsAg expression. Conversely, the levels of intrahepatic HBV replication were uniformly low in all the areas within and outside the tumor. Accordingly, HBcAg was not detectable in any liver specimens with the exception of a single non-tumorous area from a single patient. Although the low levels of HBV replication may be explained by the fact that these patients were all anti-HBe positive and under antiviral therapy with nucleos(t)ide analogues prior to surgery, the dramatic and significant decrease in HBsAg expression within the tumor cannot be explained by antiviral therapy, because nucleos(t)ide analogues have no direct effect on transcription and translation of HBsAg, which was not suppressed in the tumor-surrounding tissue. The reasons for the dramatic decrease of HBsAg within the tumor remain to be fully elucidated. Previous reports have found a lower expression of HBsAg in HCC as compared with matched non-tumorous tissues [40,41]. One of the reasons proposed to explain this observation is an increased rate of integration of HBV DNA into the host genome inside the tumor [40,42,43]. However, integration of HBV has also been reported in non-malignant hepatocytes [40,42,43]. In a recent extensive genome-wide study, HBV integration was reported in 86% of HCC and in 31% of the surrounding non-tumorous tissue [44].

Although a role for HBV integration in our cohort of patients cannot be excluded, one of the most interesting findings in our study was the down-regulation within the tumor of canonical pathways that are part of the nuclear receptor (NR)-associated network. This network is critically involved not only in the metabolic functions of the liver but also in the life cycle of HBV, acting as essential transcription factors for viral gene expression [45,46]. Most notably, we observed down-regulation of PPARγ coactivator-1 alpha (PGC-1α), a key transcriptional co-activator that acts as a master switcher for a large number of nuclear receptors [46]. PGC-1α serves critical functions in the control of cellular energy metabolic pathways [47]. In the liver, it is a key regulator of gluconeogenesis and fatty acid oxidative metabolism, and coordinates adaptation to metabolic alterations. Shlomai et al. [48], demonstrated in a mouse model that starvation, by turning on the gluconeogenic program, robustly induces HBV gene expression through the induction of PGC-1α, which serves as a co-activator of HBV transcription. The importance of PGC-1α in the HBV life cycle is further highlighted by the strong inhibition of HBV expression observed upon degradation of PGC-1α using the natural phenolic compound curcumin [49]. In addition to NRs, an important non-NR partner of PGC-1α, FOXO1, was found to be down-regulated in tumor tissue. FOXO1 is a central mediator of glucose metabolism in the liver that was shown to bind HBV and activate its transcription [50]. Thus, down-regulation of these genes may contribute to the sudden and significant decrease of HBsAg expression that we documented in the tumor. Through the exploitation of several liver-specific transcription factors and coactivators that regulate vital metabolic functions, HBV has acquired the ability to specifically replicate in liver tissue, reducing the risk of development of host cell resistance and leading to the definition of HBV as a “metabolovirus” [51]. However, our data documented down-regulation of essential hepatic metabolism genes, including PGC-1α and FOXO1, suggesting that the malignant hepatocyte has developed an autonomous metabolic regulation. Given the fact that PGC-1α and FOXO1 are essential coactivators of HBV transcription, we hypothesize that down-regulation of these genes may contribute to the sudden and significant decrease of HBsAg expression that we documented in the tumor. Another possibility that we cannot rule out is whether the reduced expression of HBsAg may be related to the increased proliferation rate of tumor cells.

In conclusion, microarray analysis of multiple areas of livers with HBV-associated HCC provided evidence for a remarkable change of gene expression in the perilesional area, within millimeters of the tumor margin. This sudden change is paralleled by a sharp decrease in HBsAg expression. The combined application of WLT and LCM techniques validated most of the gene expression changes associated with the tumor. Moreover, LCM allowed us to identify a series of genes not found in whole liver tissue (WLT) that may play a role in HCC, adding a new tool for dissecting pathogenesis and discovering new cancer markers.

## Additional files

Additional file 1: Figure S1. Representative images of LCM performed on tumor and non-tumor liver tissue from a patient with HBV-associated HCC. Panels A and E show the H&E stained tissues on cover-slipped glass slides, which were evaluated by a trained pathologist prior to dissection by LCM. Panels B and F show the H&E stained tissues on the PEN membrane as described in Materials and Methods; the diffraction due to the absence of a cover slip results in the various shades of brown. Panels C and G show the selected areas before performing LCM. Panels D and H show the remaining tissue after LCM. All images are at 2× magnification. Additional file 2: Table S1. Genes Differentially Expressed among the Five Liver Areas. Additional file 3: Table S2. Genes Differentially Expressed between Microdissected Malignant Hepatocytes and Non-Malignant Hepatocytes. Additional file 4: Table S3. Genes Differentially Expressed Between Malignant and Non-Malignant Liver Tissue. Additional file 5: Table S4. Differentially Expressed Genes in Common between Whole Liver Tissue (WLT) and Microdissected Hepatocytes (LCM). Additional file 6: Table S5. Differentially Expressed Genes Unique to Whole Liver Tissue. Additional file 7: Table S6. Differentially Expressed Genes Unique to Microdissected Hepatocytes. Additional file 8: Figure S2. Expression levels of four cancer testis antigen (CTA) genes (MAGEA3, CEP55, TTK and NUF2) of malignant hepatocytes (red bars) and non-malignant hepatocytes (blue bars), isolated by LCM from 10 HCC patients (numbered 1 to 10). All four CTA genes were up-regulated in malignant hepatocytes, with fold changes > 4. However, as shown by the plot, the expression was higher in some patients (leftmost cases) than in others (rightmost cases). Additional file 9: Figure S3. Expression of four CTA genes (MAGEA3, CEP55, TTK and NUF2) in tumor and non-tumorous liver tissue of 10 HCC patients (numbered 1 to 10) evaluated by RT-qPCR. Data are represented by –ΔΔCt (red bars), where ΔΔCt = (CtTarget - CtGAPDH )Tumor - (CtTarget - CtGAPDH)Non-tumor. Results are mean ± standard error.

## Abbreviations

HBV: Hepatitis B virus
HCC: Hepatocellular carcinoma
GEP: Gene expression profiling
WLT: Whole liver tissue
LCM: Laser capture microdissection
FDR: False discovery rate
FC: Fold change
