# Supplementary figures and images for: Viral expression and molecular profiling in liver tissue versus microdissected hepatocytes in hepatitis B virus - associated hepatocellular carcinoma
Source: J Transl Med. 2014 Aug 21;12:230. doi: 10.1186/s12967-014-0230-1 (PMC4142136; doi:10.1186/s12967-014-0230-1)

**Figure S1**

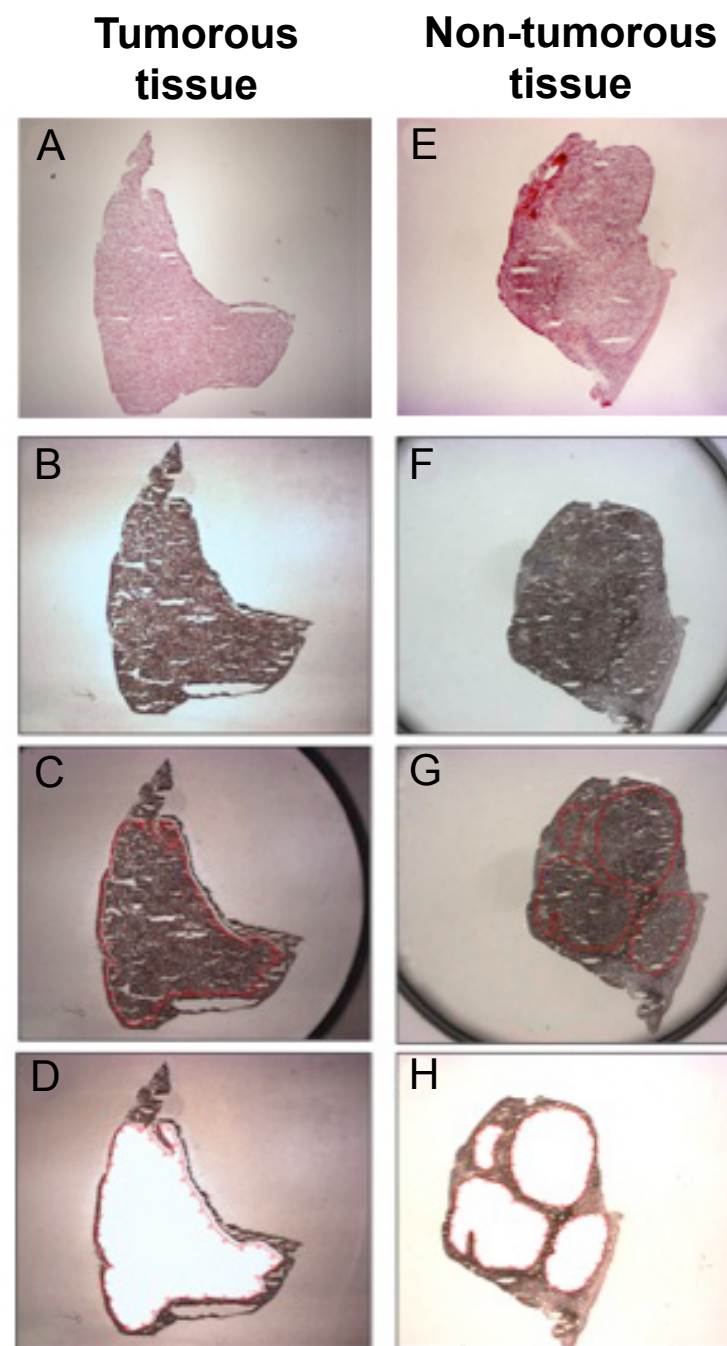

Supplement: Additional file 1: Figure S1. — Representative images of LCM performed on tumor and non-tumor liver tissue from a patient with HBV-associated HCC. Panels A and E show the H&E stained tissues on cover-slipped glass slides, which were evaluated by a trained pathologist prior to dissection by LCM. Panels B and F show the H&E stained tissues on the PEN membrane as described in Materials and Methods; the diffraction due to the absence of a cover slip results in the various shades of brown. Panels C and G show the selected areas before performing LCM. Panels D and H show the remaining tissue after LCM. All images are at 2× magnification. [file 12967_2014_230_MOESM1_ESM.pdf]

**Figure S2**

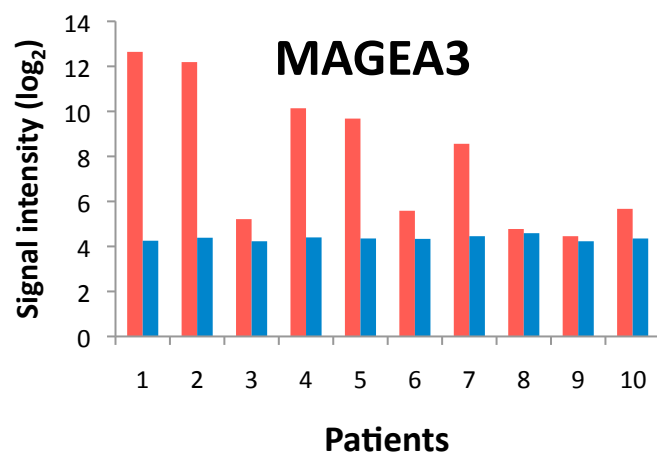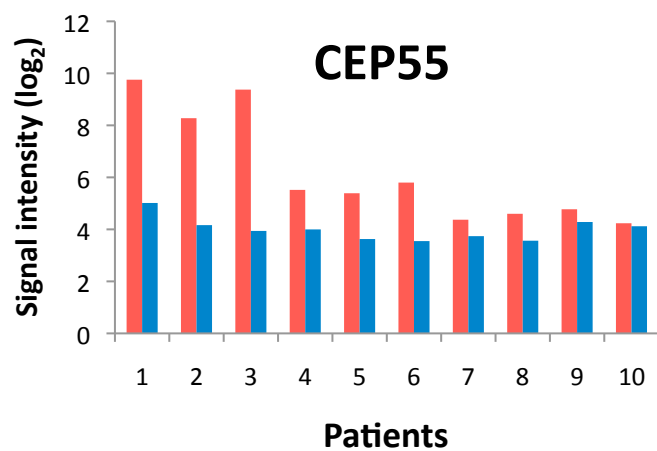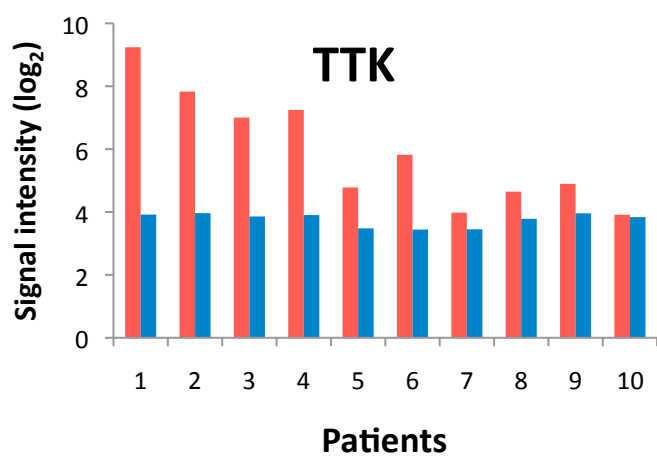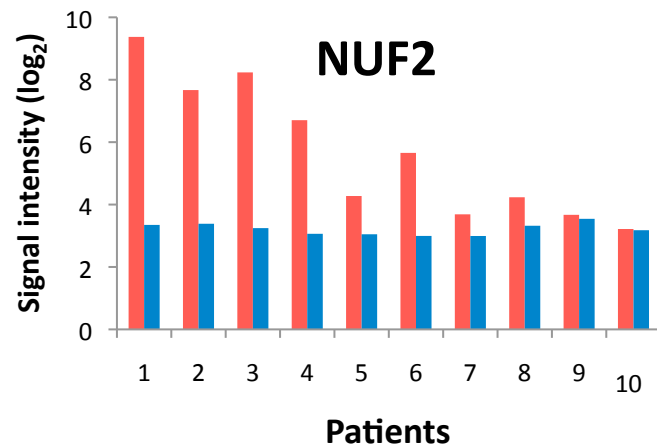

Supplement: Additional file 8: Figure S2. — Expression levels of four cancer testis antigen (CTA) genes (MAGEA3, CEP55, TTK and NUF2) of malignant hepatocytes (red bars) and non-malignant hepatocytes (blue bars), isolated by LCM from 10 HCC patients (numbered 1 to 10). All four CTA genes were up-regulated in malignant hepatocytes, with fold changes > 4. However, as shown by the plot, the expression was higher in some patients (leftmost cases) than in others (rightmost cases). [file 12967_2014_230_MOESM8_ESM.pdf]

**Figure S3**

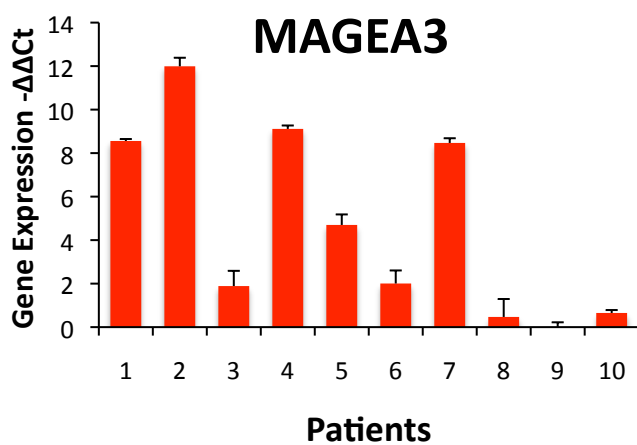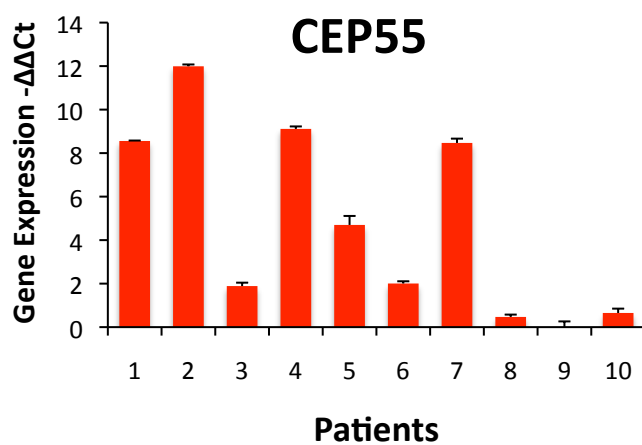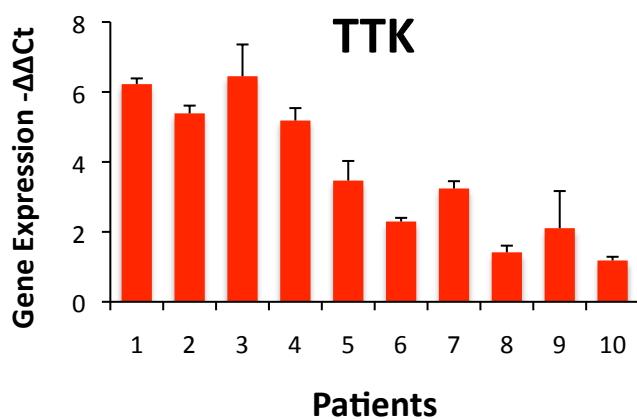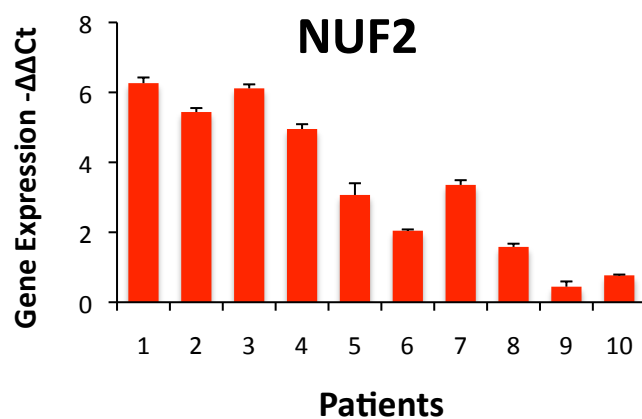

Supplement: Additional file 9: Figure S3. — Expression of four CTA genes (MAGEA3, CEP55, TTK and NUF2) in tumor and non-tumorous liver tissue of 10 HCC patients (numbered 1 to 10) evaluated by RT-qPCR. Data are represented by –ΔΔCt (red bars), where ΔΔCt = (CtTarget - CtGAPDH )Tumor - (CtTarget - CtGAPDH)Non-tumor. Results are mean ± standard error. [file 12967_2014_230_MOESM9_ESM.pdf]
