# Supplementary material for: Viral expression and molecular profiling in liver tissue versus microdissected hepatocytes in hepatitis B virus - associated hepatocellular carcinoma
Source: J Transl Med. 2014 Aug 21;12:230. doi: 10.1186/s12967-014-0230-1 (PMC4142136; doi:10.1186/s12967-014-0230-1)
Supplement: Additional file 2: Table S1. — Genes Differentially Expressed among the Five Liver Areas. [file 12967_2014_230_MOESM2_ESM.docx]

| **Table S1.** Genes Differentially Expressed Among the Five Liver Areas | |
| --- | --- |
| Gene Symbol | Gene Title |
| A1BG | alpha-1-B glycoprotein |
| A1CF | APOBEC1 complementation factor |
| A2M | alpha-2-macroglobulin |
| AADAT | aminoadipate aminotransferase |
| AASS | aminoadipate-semialdehyde synthase |
| ABAT | 4-aminobutyrate aminotransferase |
| ABCA6 | ATP-binding cassette, sub-family A (ABC1), member 6 |
| ABCA8 | ATP-binding cassette, sub-family A (ABC1), member 8 |
| ABCB1 | ATP-binding cassette, sub-family B (MDR/TAP), member 1 |
| ABCB4 | ATP-binding cassette, sub-family B (MDR/TAP), member 4 |
| ABCB6 | ATP-binding cassette, sub-family B (MDR/TAP), member 6 |
| ABCC5 | ATP-binding cassette, sub-family C (CFTR/MRP), member 5 |
| ABCG5 | ATP-binding cassette, sub-family G (WHITE), member 5 |
| ABHD15 | abhydrolase domain containing 15 |
| ABHD2 | abhydrolase domain containing 2 |
| ABI3BP | ABI family, member 3 (NESH) binding protein |
| ABLIM3 | actin binding LIM protein family, member 3 |
| ACAA1 | acetyl-CoA acyltransferase 1 |
| ACAA2 | acetyl-CoA acyltransferase 2 |
| ACACB | acetyl-CoA carboxylase beta |
| ACADL | acyl-CoA dehydrogenase, long chain |
| ACADM | acyl-CoA dehydrogenase, C-4 to C-12 straight chain |
| ACADSB | acyl-CoA dehydrogenase, short/branched chain |
| ACAT1 | acetyl-CoA acetyltransferase 1 |
| ACMSD | aminocarboxymuconate semialdehyde decarboxylase |
| ACOT12 | acyl-CoA thioesterase 12 |
| ACOX1 | acyl-CoA oxidase 1, palmitoyl |
| ACOX2 | acyl-CoA oxidase 2, branched chain |
| ACSL1 | acyl-CoA synthetase long-chain family member 1 |
| ACSL4 | acyl-CoA synthetase long-chain family member 4 |
| ACSL6 | acyl-CoA synthetase long-chain family member 6 |
| ACSM2A | acyl-CoA synthetase medium-chain family member 2A |
| ACSM3 | acyl-CoA synthetase medium-chain family member 3 |
| ACSM5 | acyl-CoA synthetase medium-chain family member 5 |
| ADAM9 | ADAM metallopeptidase domain 9 |
| ADAMDEC1 | ADAM-like, decysin 1 |
| ADAMTS1 | ADAM metallopeptidase with thrombospondin type 1 motif, 1 |
| ADAMTS13 | ADAM metallopeptidase with thrombospondin type 1 motif, 13 |
| ADAMTS17 | ADAM metallopeptidase with thrombospondin type 1 motif, 17 |
| ADAMTSL2 | ADAMTS-like 2 |
| ADAMTSL3 | ADAMTS-like 3 |
| ADCY1 | adenylate cyclase 1 (brain) |
| ADH1A | alcohol dehydrogenase 1A (class I), alpha polypeptide |
| ADH1B | alcohol dehydrogenase 1B (class I), beta polypeptide |
| ADH1C | alcohol dehydrogenase 1C (class I), gamma polypeptide |
| ADH4 | alcohol dehydrogenase 4 (class II), pi polypeptide |
| ADH6 | alcohol dehydrogenase 6 (class V) |
| ADI1 | acireductone dioxygenase 1 |
| ADK | adenosine kinase |
| ADORA3 | adenosine A3 receptor |
| ADRA1A | adrenoceptor alpha 1A |
| AFM | afamin |
| AGBL2 | ATP/GTP binding protein-like 2 |
| AGBL3 | ATP/GTP binding protein-like 3 |
| AGPAT4 | 1-acylglycerol-3-phosphate O-acyltransferase 4 (lysophosphatidic acid acyltransferase, delta) |
| AGPAT9 | 1-acylglycerol-3-phosphate O-acyltransferase 9 |
| AGTR1 | angiotensin II receptor, type 1 |
| AGXT | alanine-glyoxylate aminotransferase |
| AGXT2 | alanine--glyoxylate aminotransferase 2 |
| AGXT2L1 | alanine-glyoxylate aminotransferase 2-like 1 |
| AHSA2 | AHA1, activator of heat shock 90kDa protein ATPase homolog 2 (yeast) |
| AHSG | alpha-2-HS-glycoprotein |
| AIM1L | absent in melanoma 1-like |
| AJUBA | ajuba LIM protein |
| AKAP12 | A kinase (PRKA) anchor protein 12 |
| AKR1B10 | aldo-keto reductase family 1, member B10 (aldose reductase) |
| AKR1C1/AKR1C2 | aldo-keto reductase family 1, member C2 |
| AKR1C3 | aldo-keto reductase family 1, member C3 |
| AKR1D1 | aldo-keto reductase family 1, member D1 |
| AKR7A3 | aldo-keto reductase family 7, member A3 (aflatoxin aldehyde reductase) |
| ALAS1 | aminolevulinate, delta-, synthase 1 |
| ALB | albumin |
| ALDH1A2 | aldehyde dehydrogenase 1 family, member A2 |
| ALDH2 | aldehyde dehydrogenase 2 family (mitochondrial) |
| ALDH3A1 | aldehyde dehydrogenase 3 family, member A1 |
| ALDH5A1 | aldehyde dehydrogenase 5 family, member A1 |
| ALDH6A1 | aldehyde dehydrogenase 6 family, member A1 |
| ALDH7A1 | aldehyde dehydrogenase 7 family, member A1 |
| ALDH8A1 | aldehyde dehydrogenase 8 family, member A1 |
| ALDOA | aldolase A, fructose-bisphosphate |
| ALDOB | aldolase B, fructose-bisphosphate |
| ALLC | allantoicase |
| ALPK3 | alpha-kinase 3 |
| AMDHD1 | amidohydrolase domain containing 1 |
| AMOTL2 | angiomotin like 2 |
| ANG | angiogenin, ribonuclease, RNase A family, 5 |
| ANGPTL1 | angiopoietin-like 1 |
| ANGPTL3 | angiopoietin-like 3 |
| ANGPTL4 | angiopoietin-like 4 |
| ANGPTL6 | angiopoietin-like 6 |
| ANK3 | ankyrin 3, node of Ranvier (ankyrin G) |
| ANKRD27 | ankyrin repeat domain 27 (VPS9 domain) |
| ANKRD36BP2 | ankyrin repeat domain 36B pseudogene 2 |
| ANKRD55 | ankyrin repeat domain 55 |
| ANLN | anillin, actin binding protein |
| ANO1 | anoctamin 1, calcium activated chloride channel |
| ANP32E | acidic (leucine-rich) nuclear phosphoprotein 32 family, member E |
| ANXA10 | annexin A10 |
| ANXA2 | annexin A2 |
| ANXA3 | annexin A3 |
| AOX1 | aldehyde oxidase 1 |
| APBA1 | amyloid beta (A4) precursor protein-binding, family A, member 1 |
| APOA1 | apolipoprotein A-I |
| APOA5 | apolipoprotein A-V |
| APOBEC3B | apolipoprotein B mRNA editing enzyme, catalytic polypeptide-like 3B |
| APOC3 | apolipoprotein C-III |
| APOF | apolipoprotein F |
| APOH | apolipoprotein H (beta-2-glycoprotein I) |
| APOL6 | apolipoprotein L, 6 |
| APOM | apolipoprotein M |
| AQP1 | aquaporin 1 (Colton blood group) |
| AQP3 | aquaporin 3 (Gill blood group) |
| AQP4 | aquaporin 4 |
| AQP9 | aquaporin 9 |
| AR | androgen receptor |
| ARAP2 | ArfGAP with RhoGAP domain, ankyrin repeat and PH domain 2 |
| ARG1 | arginase, liver |
| ARHGAP18 | Rho GTPase activating protein 18 |
| ARHGAP44 | Rho GTPase activating protein 44 |
| ARHGEF26 | Rho guanine nucleotide exchange factor (GEF) 26 |
| ARID4A | AT rich interactive domain 4A (RBP1-like) |
| ARRB1 | arrestin, beta 1 |
| ARRDC4 | arrestin domain containing 4 |
| ART4 | ADP-ribosyltransferase 4 (Dombrock blood group) |
| ASAP1 | ArfGAP with SH3 domain, ankyrin repeat and PH domain 1 |
| ASAP2 | ArfGAP with SH3 domain, ankyrin repeat and PH domain 2 |
| ASCL1 | achaete-scute complex homolog 1 (Drosophila) |
| ASGR1 | asialoglycoprotein receptor 1 |
| ASGR2 | asialoglycoprotein receptor 2 |
| ASL | argininosuccinate lyase |
| ASPA | aspartoacylase |
| ASPH | aspartate beta-hydroxylase |
| ASPM | asp (abnormal spindle) homolog, microcephaly associated (Drosophila) |
| ASPN | asporin |
| ASRGL1 | asparaginase like 1 |
| ASS1 | argininosuccinate synthase 1 |
| ATAD2 | ATPase family, AAA domain containing 2 |
| ATF3 | activating transcription factor 3 |
| ATF5 | activating transcription factor 5 |
| ATOH8 | atonal homolog 8 (Drosophila) |
| ATP11C | ATPase, class VI, type 11C |
| ATP2B2 | ATPase, Ca++ transporting, plasma membrane 2 |
| ATP5G3 | ATP synthase, H+ transporting, mitochondrial Fo complex, subunit C3 (subunit 9) |
| ATP6V1C1 | ATPase, H+ transporting, lysosomal 42kDa, V1 subunit C1 |
| ATP7B | ATPase, Cu++ transporting, beta polypeptide |
| AURKA | aurora kinase A |
| AUTS2 | autism susceptibility candidate 2 |
| AVPI1 | arginine vasopressin-induced 1 |
| AVPR1A | arginine vasopressin receptor 1A |
| AXIN2 | axin 2 |
| AZGP1 | alpha-2-glycoprotein 1, zinc-binding |
| B3GNT5 | UDP-GlcNAc:betaGal beta-1,3-N-acetylglucosaminyltransferase 5 |
| B4GALT6 | UDP-Gal:betaGlcNAc beta 1,4- galactosyltransferase, polypeptide 6 |
| BAAT | bile acid CoA: amino acid N-acyltransferase (glycine N-choloyltransferase) |
| BACH2 | BTB and CNC homology 1, basic leucine zipper transcription factor 2 |
| BAG2 | BCL2-associated athanogene 2 |
| BAI3 | brain-specific angiogenesis inhibitor 3 |
| BAIAP2-AS1 | BAIAP2 antisense RNA 1 (head to head) |
| BARD1 | BRCA1 associated RING domain 1 |
| BBOX1 | butyrobetaine (gamma), 2-oxoglutarate dioxygenase (gamma-butyrobetaine hydroxylase) 1 |
| BCAT1 | branched chain amino-acid transaminase 1, cytosolic |
| BCHE | butyrylcholinesterase |
| BCKDHB | branched chain keto acid dehydrogenase E1, beta polypeptide |
| BCO2 | beta-carotene oxygenase 2 |
| BDH1 | 3-hydroxybutyrate dehydrogenase, type 1 |
| BDH2 | 3-hydroxybutyrate dehydrogenase, type 2 |
| BEX1 | brain expressed, X-linked 1 |
| BEX2 | brain expressed X-linked 2 |
| BGN | biglycan |
| BHLHE22 | basic helix-loop-helix family, member e22 |
| BHLHE40 | basic helix-loop-helix family, member e40 |
| BHMT | betaine--homocysteine S-methyltransferase |
| BHMT2 | betaine--homocysteine S-methyltransferase 2 |
| BICD1 | bicaudal D homolog 1 (Drosophila) |
| BIRC5 | baculoviral IAP repeat containing 5 |
| BLNK | B-cell linker |
| BMPER | BMP binding endothelial regulator |
| BOP1 | block of proliferation 1 |
| BRCA1 | breast cancer 1, early onset |
| BRIP1 | BRCA1 interacting protein C-terminal helicase 1 |
| BTD | biotinidase |
| BTG2 | BTG family, member 2 |
| BUB1B | budding uninhibited by benzimidazoles 1 homolog beta (yeast) |
| C11orf54 | chromosome 11 open reading frame 54 |
| C11orf93 | chromosome 11 open reading frame 93 |
| C11orf96 | chromosome 11 open reading frame 96 |
| C12orf75 | chromosome 12 open reading frame 75 |
| C14orf182 | chromosome 14 open reading frame 182 |
| C19orf77 | chromosome 19 open reading frame 77 |
| C1orf168 | chromosome 1 open reading frame 168 |
| C1orf43 | chromosome 1 open reading frame 43 |
| C1orf85 | chromosome 1 open reading frame 85 |
| C1R | complement component 1, r subcomponent |
| C1RL | complement component 1, r subcomponent-like |
| C1S | complement component 1, s subcomponent |
| C2orf40 | chromosome 2 open reading frame 40 |
| C3P1 | complement component 3 precursor pseudogene |
| C4BPB | complement component 4 binding protein, beta |
| C5 | complement component 5 |
| C5orf4 | chromosome 5 open reading frame 4 |
| C5orf54 | chromosome 5 open reading frame 54 |
| C6 | complement component 6 |
| C6orf123 | chromosome 6 open reading frame 123 |
| C7 | complement component 7 |
| C7orf29 | chromosome 7 open reading frame 29 |
| C8A | complement component 8, alpha polypeptide |
| C8B | complement component 8, beta polypeptide |
| C8orf4 | chromosome 8 open reading frame 4 |
| C8orf40 | chromosome 8 open reading frame 40 |
| C9 | complement component 9 |
| C9orf152 | chromosome 9 open reading frame 152 |
| CA12 | carbonic anhydrase XII |
| CA2 | carbonic anhydrase II |
| CA5A | carbonic anhydrase VA, mitochondrial |
| CAND2 | cullin-associated and neddylation-dissociated 2 (putative) |
| CAP2 | CAP, adenylate cyclase-associated protein, 2 (yeast) |
| CAPG | capping protein (actin filament), gelsolin-like |
| CAT | catalase |
| CBR4 | carbonyl reductase 4 |
| CBS | cystathionine-beta-synthase |
| CCBE1 | collagen and calcium binding EGF domains 1 |
| CCDC113 | coiled-coil domain containing 113 |
| CCDC3 | coiled-coil domain containing 3 |
| CCDC34 | coiled-coil domain containing 34 |
| CCDC71L | coiled-coil domain containing 71-like |
| CCDC88A | coiled-coil domain containing 88A |
| CCL14 | chemokine (C-C motif) ligand 14 |
| CCL19 | chemokine (C-C motif) ligand 19 |
| CCL2 | chemokine (C-C motif) ligand 2 |
| CCL20 | chemokine (C-C motif) ligand 20 |
| CCL3 | chemokine (C-C motif) ligand 3 |
| CCL4 | chemokine (C-C motif) ligand 4 |
| CCL5 | chemokine (C-C motif) ligand 5 |
| CCNA2 | cyclin A2 |
| CCNB1 | cyclin B1 |
| CCNB2 | cyclin B2 |
| CCNE2 | cyclin E2 |
| CD109 | CD109 molecule |
| CD14 | CD14 molecule |
| CD160 | CD160 molecule |
| CD1D | CD1d molecule |
| CD302 | CD302 molecule |
| CD5L | CD5 molecule-like |
| CD69 | CD69 molecule |
| CD8A | CD8a molecule |
| CDA | cytidine deaminase |
| CDC14B | CDC14 cell division cycle 14 homolog B (S. cerevisiae) |
| CDC20 | cell division cycle 20 homolog (S. cerevisiae) |
| CDC37L1 | cell division cycle 37 homolog (S. cerevisiae)-like 1 |
| CDC7 | cell division cycle 7 homolog (S. cerevisiae) |
| CDCA3 | cell division cycle associated 3 |
| CDH1 | cadherin 1, type 1, E-cadherin (epithelial) |
| CDH19 | cadherin 19, type 2 |
| CDHR2 | cadherin-related family member 2 |
| CDK1 | cyclin-dependent kinase 1 |
| CDK5RAP2 | CDK5 regulatory subunit associated protein 2 |
| CDK6 | cyclin-dependent kinase 6 |
| CDKN1C | cyclin-dependent kinase inhibitor 1C (p57, Kip2) |
| CDKN2A | cyclin-dependent kinase inhibitor 2A |
| CDKN2B | cyclin-dependent kinase inhibitor 2B (p15, inhibits CDK4) |
| CDKN2C | cyclin-dependent kinase inhibitor 2C (p18, inhibits CDK4) |
| CDKN3 | cyclin-dependent kinase inhibitor 3 |
| CDO1 | cysteine dioxygenase, type I |
| CEBPD | CCAAT/enhancer binding protein (C/EBP), delta |
| CENPF | centromere protein F, 350/400kDa (mitosin) |
| CENPJ | centromere protein J |
| CENPK | centromere protein K |
| CENPW | centromere protein W |
| CEP152 | centrosomal protein 152kDa |
| CEP41 | centrosomal protein 41kDa |
| CES1P1 | carboxylesterase 1 pseudogene 1 |
| CETP | cholesteryl ester transfer protein, plasma |
| CFHR2 | complement factor H-related 2 |
| CFHR3 | complement factor H-related 3 |
| CFHR4 | complement factor H-related 4 |
| CFL2 | cofilin 2 (muscle) |
| CFP | complement factor properdin |
| CFTR | cystic fibrosis transmembrane conductance regulator (ATP-binding cassette sub-family C, member 7) |
| CGNL1 | cingulin-like 1 |
| CH25H | cholesterol 25-hydroxylase |
| CHI3L1 | chitinase 3-like 1 (cartilage glycoprotein-39) |
| CHML | choroideremia-like (Rab escort protein 2) |
| CHRD | chordin |
| CHRDL1 | chordin-like 1 |
| CHRM3 | cholinergic receptor, muscarinic 3 |
| CHST4 | carbohydrate (N-acetylglucosamine 6-O) sulfotransferase 4 |
| CHST9 | carbohydrate (N-acetylgalactosamine 4-0) sulfotransferase 9 |
| CIDEB | cell death-inducing DFFA-like effector b |
| CISH | cytokine inducible SH2-containing protein |
| CKAP2 | cytoskeleton associated protein 2 |
| CKAP4 | cytoskeleton-associated protein 4 |
| CLDN10 | claudin 10 |
| CLEC1B | C-type lectin domain family 1, member B |
| CLEC4G | C-type lectin domain family 4, member G |
| CLEC4M | C-type lectin domain family 4, member M |
| CLGN | calmegin |
| CLIC6 | chloride intracellular channel 6 |
| CLN8 | ceroid-lipofuscinosis, neuronal 8 (epilepsy, progressive with mental retardation) |
| CLRN3 | clarin 3 |
| CLU | clusterin |
| CLYBL | citrate lyase beta like |
| CMBL | carboxymethylenebutenolidase homolog (Pseudomonas) |
| CMC4 | C-x(9)-C motif containing 4 homolog (S. cerevisiae) |
| CNDP1 | carnosine dipeptidase 1 (metallopeptidase M20 family) |
| CNGA1 | cyclic nucleotide gated channel alpha 1 |
| CNKSR2 | connector enhancer of kinase suppressor of Ras 2 |
| CNTLN | centlein, centrosomal protein |
| CNTN3 | contactin 3 (plasmacytoma associated) |
| CNTN4 | contactin 4 |
| COBLL1 | cordon-bleu WH2 repeat protein-like 1 |
| COCH | coagulation factor C homolog, cochlin (Limulus polyphemus) |
| COG2 | component of oligomeric golgi complex 2 |
| COL15A1 | collagen, type XV, alpha 1 |
| COL1A1 | collagen, type I, alpha 1 |
| COL4A1 | collagen, type IV, alpha 1 |
| COL4A2 | collagen, type IV, alpha 2 |
| COL5A2 | collagen, type V, alpha 2 |
| COL6A6 | collagen, type VI, alpha 6 |
| COLEC10 | collectin sub-family member 10 (C-type lectin) |
| COLEC11 | collectin sub-family member 11 |
| COX7B | cytochrome c oxidase subunit VIIb |
| CP | ceruloplasmin (ferroxidase) |
| CPD | carboxypeptidase D |
| CPEB3 | cytoplasmic polyadenylation element binding protein 3 |
| CPED1 | cadherin-like and PC-esterase domain containing 1 |
| CPN1 | carboxypeptidase N, polypeptide 1 |
| CPS1 | carbamoyl-phosphate synthase 1, mitochondrial |
| CR1 | complement component (3b/4b) receptor 1 (Knops blood group) |
| CREM | cAMP responsive element modulator |
| CRHBP | corticotropin releasing hormone binding protein |
| CRISPLD2 | cysteine-rich secretory protein LCCL domain containing 2 |
| CRNDE | colorectal neoplasia differentially expressed (non-protein coding) |
| CROCCP2 | ciliary rootlet coiled-coil, rootletin pseudogene 2 |
| CROT | carnitine O-octanoyltransferase |
| CRP | C-reactive protein, pentraxin-related |
| CRYL1 | crystallin, lambda 1 |
| CSAD | cysteine sulfinic acid decarboxylase |
| CSRNP1 | cysteine-serine-rich nuclear protein 1 |
| CSRP2 | cysteine and glycine-rich protein 2 |
| CTCF | CCCTC-binding factor (zinc finger protein) |
| CTGF | connective tissue growth factor |
| CTH | cystathionase (cystathionine gamma-lyase) |
| CTHRC1 | collagen triple helix repeat containing 1 |
| CTNNA2 | catenin (cadherin-associated protein), alpha 2 |
| CTSA | cathepsin A |
| CTSC | cathepsin C |
| CUX2 | cut-like homeobox 2 |
| CXCL12 | chemokine (C-X-C motif) ligand 12 |
| CXCL14 | chemokine (C-X-C motif) ligand 14 |
| CXCL2 | chemokine (C-X-C motif) ligand 2 |
| CXCR7 | chemokine (C-X-C motif) receptor 7 |
| CYFIP2 | cytoplasmic FMR1 interacting protein 2 |
| CYP1A2 | cytochrome P450, family 1, subfamily A, polypeptide 2 |
| CYP26A1 | cytochrome P450, family 26, subfamily A, polypeptide 1 |
| CYP2A13/CYP2A6 | cytochrome P450, family 2, subfamily A, polypeptide 6 |
| CYP2A7 | cytochrome P450, family 2, subfamily A, polypeptide 7 |
| CYP2B6 | cytochrome P450, family 2, subfamily B, polypeptide 6 |
| CYP2B7P1 | cytochrome P450, family 2, subfamily B, polypeptide 7 pseudogene 1 |
| CYP2C18 | cytochrome P450, family 2, subfamily C, polypeptide 18 |
| CYP2C19 | cytochrome P450, family 2, subfamily C, polypeptide 19 |
| CYP2C8 | cytochrome P450, family 2, subfamily C, polypeptide 8 |
| CYP2C9 | cytochrome P450, family 2, subfamily C, polypeptide 9 |
| CYP2D6 | cytochrome P450, family 2, subfamily D, polypeptide 6 |
| CYP2E1 | cytochrome P450, family 2, subfamily E, polypeptide 1 |
| CYP2J2 | cytochrome P450, family 2, subfamily J, polypeptide 2 |
| CYP39A1 | cytochrome P450, family 39, subfamily A, polypeptide 1 |
| CYP3A4 | cytochrome P450, family 3, subfamily A, polypeptide 4 |
| CYP3A43 | cytochrome P450, family 3, subfamily A, polypeptide 43 |
| CYP3A5 | cytochrome P450, family 3, subfamily A, polypeptide 5 |
| CYP3A7 | cytochrome P450, family 3, subfamily A, polypeptide 7 |
| CYP3A7-CYP3AP1 | CYP3A7-CYP3AP1 readthrough |
| CYP4A11 | cytochrome P450, family 4, subfamily A, polypeptide 11 |
| CYP4A22 | cytochrome P450, family 4, subfamily A, polypeptide 22 |
| CYP4F12 | cytochrome P450, family 4, subfamily F, polypeptide 12 |
| CYP4F2 | cytochrome P450, family 4, subfamily F, polypeptide 2 |
| CYP4F3 | cytochrome P450, family 4, subfamily F, polypeptide 3 |
| CYP4V2 | cytochrome P450, family 4, subfamily V, polypeptide 2 |
| CYP4X1 | cytochrome P450, family 4, subfamily X, polypeptide 1 |
| CYP8B1 | cytochrome P450, family 8, subfamily B, polypeptide 1 |
| CYR61 | cysteine-rich, angiogenic inducer, 61 |
| DAB1 | disabled homolog 1 (Drosophila) |
| DAB2 | disabled homolog 2, mitogen-responsive phosphoprotein (Drosophila) |
| DACH1 | dachshund homolog 1 (Drosophila) |
| DAK | dihydroxyacetone kinase 2 homolog (S. cerevisiae) |
| DAO | D-amino-acid oxidase |
| DBH | dopamine beta-hydroxylase (dopamine beta-monooxygenase) |
| DBH-AS1 | DBH antisense RNA 1 |
| DCN | decorin |
| DCPS | decapping enzyme, scavenger |
| DCTD | dCMP deaminase |
| DCXR | dicarbonyl/L-xylulose reductase |
| DECR1 | 2,4-dienoyl CoA reductase 1, mitochondrial |
| DEFB1 | defensin, beta 1 |
| DEPDC1B | DEP domain containing 1B |
| DEPDC7 | DEP domain containing 7 |
| DGAT2 | diacylglycerol O-acyltransferase 2 |
| DHODH | dihydroorotate dehydrogenase (quinone) |
| DHRS1 | dehydrogenase/reductase (SDR family) member 1 |
| DHRS2 | dehydrogenase/reductase (SDR family) member 2 |
| DHTKD1 | dehydrogenase E1 and transketolase domain containing 1 |
| DKK3 | dickkopf 3 homolog (Xenopus laevis) |
| DLC1 | deleted in liver cancer 1 |
| DLG5 | discs, large homolog 5 (Drosophila) |
| DLGAP5 | discs, large (Drosophila) homolog-associated protein 5 |
| DLL1 | delta-like 1 (Drosophila) |
| DMD | dystrophin |
| DMGDH | dimethylglycine dehydrogenase |
| DNAH12 | dynein, axonemal, heavy chain 12 |
| DNAJC10 | DnaJ (Hsp40) homolog, subfamily C, member 10 |
| DNAJC25 | DnaJ (Hsp40) homolog, subfamily C , member 25 |
| DNAJC3-AS1 | DNAJC3 antisense RNA 1 (head to head) |
| DNAJC6 | DnaJ (Hsp40) homolog, subfamily C, member 6 |
| DNALI1 | dynein, axonemal, light intermediate chain 1 |
| DNASE1L3 | deoxyribonuclease I-like 3 |
| DNM3 | dynamin 3 |
| DNM3OS | DNM3 opposite strand/antisense RNA |
| DPF3 | D4, zinc and double PHD fingers, family 3 |
| DPT | dermatopontin |
| DPYS | dihydropyrimidinase |
| DSEL | dermatan sulfate epimerase-like |
| DTL | denticleless E3 ubiquitin protein ligase homolog (Drosophila) |
| DTNA | dystrobrevin, alpha |
| DTX1 | deltex homolog 1 (Drosophila) |
| DUSP1 | dual specificity phosphatase 1 |
| DUSP10 | dual specificity phosphatase 10 |
| DUSP2 | dual specificity phosphatase 2 |
| DUSP5 | dual specificity phosphatase 5 |
| DUT | deoxyuridine triphosphatase |
| DYNC1I1 | dynein, cytoplasmic 1, intermediate chain 1 |
| E2F3 | E2F transcription factor 3 |
| E2F7 | E2F transcription factor 7 |
| EBF1 | early B-cell factor 1 |
| ECHDC2 | enoyl CoA hydratase domain containing 2 |
| ECI2 | enoyl-CoA delta isomerase 2 |
| ECM1 | extracellular matrix protein 1 |
| ECT2 | epithelial cell transforming sequence 2 oncogene |
| EDEM1 | ER degradation enhancer, mannosidase alpha-like 1 |
| EDIL3 | EGF-like repeats and discoidin I-like domains 3 |
| EDNRB | endothelin receptor type B |
| EFCAB2 | EF-hand calcium binding domain 2 |
| EGFR | epidermal growth factor receptor |
| EGR1 | early growth response 1 |
| EGR2 | early growth response 2 |
| EHD3 | EH-domain containing 3 |
| EHHADH | enoyl-CoA, hydratase/3-hydroxyacyl CoA dehydrogenase |
| EID3 | EP300 interacting inhibitor of differentiation 3 |
| EIF4E3 | eukaryotic translation initiation factor 4E family member 3 |
| EIF5 | eukaryotic translation initiation factor 5 |
| EIF5A2 | eukaryotic translation initiation factor 5A2 |
| ELF3 | E74-like factor 3 (ets domain transcription factor, epithelial-specific ) |
| EME1 | essential meiotic endonuclease 1 homolog 1 (S. pombe) |
| EMILIN2 | elastin microfibril interfacer 2 |
| EML6 | echinoderm microtubule associated protein like 6 |
| ENAH | enabled homolog (Drosophila) |
| ENDOG | endonuclease G |
| ENO3 | enolase 3 (beta, muscle) |
| ENPEP | glutamyl aminopeptidase (aminopeptidase A) |
| ENTPD5 | ectonucleoside triphosphate diphosphohydrolase 5 |
| EPB41L4A | erythrocyte membrane protein band 4.1 like 4A |
| EPB41L4B | erythrocyte membrane protein band 4.1 like 4B |
| EPB41L5 | erythrocyte membrane protein band 4.1 like 5 |
| EPCAM | epithelial cell adhesion molecule |
| EPDR1 | ependymin related protein 1 (zebrafish) |
| EPHA2 | EPH receptor A2 |
| EPHA3 | EPH receptor A3 |
| EPHX2 | epoxide hydrolase 2, cytoplasmic |
| ERLIN1 | ER lipid raft associated 1 |
| ERRFI1 | ERBB receptor feedback inhibitor 1 |
| ESR1 | estrogen receptor 1 |
| ESRP1 | epithelial splicing regulatory protein 1 |
| ESRP2 | epithelial splicing regulatory protein 2 |
| ETFDH | electron-transferring-flavoprotein dehydrogenase |
| ETS2 | v-ets erythroblastosis virus E26 oncogene homolog 2 (avian) |
| ETV1 | ets variant 1 |
| EVA1A | eva-1 homolog A (C. elegans) |
| EVC | Ellis van Creveld syndrome |
| EXOC3L4 | exocyst complex component 3-like 4 |
| EZH2 | enhancer of zeste homolog 2 (Drosophila) |
| F11 | coagulation factor XI |
| F12 | coagulation factor XII (Hageman factor) |
| F13A1 | coagulation factor XIII, A1 polypeptide |
| F13B | coagulation factor XIII, B polypeptide |
| F2 | coagulation factor II (thrombin) |
| F7 | coagulation factor VII (serum prothrombin conversion accelerator) |
| F8 | coagulation factor VIII, procoagulant component |
| F9 | coagulation factor IX |
| FABP1 | fatty acid binding protein 1, liver |
| FABP5 | fatty acid binding protein 5 (psoriasis-associated) |
| FAH | fumarylacetoacetate hydrolase (fumarylacetoacetase) |
| FAHD2A | fumarylacetoacetate hydrolase domain containing 2A |
| FAM110C | family with sequence similarity 110, member C |
| FAM134B | family with sequence similarity 134, member B |
| FAM13A | family with sequence similarity 13, member A |
| FAM149A | family with sequence similarity 149, member A |
| FAM150B | family with sequence similarity 150, member B |
| FAM169A | family with sequence similarity 169, member A |
| FAM46C | family with sequence similarity 46, member C |
| FAM49B | family with sequence similarity 49, member B |
| FAM50A | family with sequence similarity 50, member A |
| FAM65C | family with sequence similarity 65, member C |
| FAM72D | family with sequence similarity 72, member D |
| FAM82A1 | family with sequence similarity 82, member A1 |
| FAM83D | family with sequence similarity 83, member D |
| FANCD2 | Fanconi anemia, complementation group D2 |
| FANCI | Fanconi anemia, complementation group I |
| FAT4 | FAT tumor suppressor homolog 4 (Drosophila) |
| FBLN5 | fibulin 5 |
| FBP1 | fructose-1,6-bisphosphatase 1 |
| FBXO32 | F-box protein 32 |
| FBXO8 | F-box protein 8 |
| FCGBP | Fc fragment of IgG binding protein |
| FCN2 | ficolin (collagen/fibrinogen domain containing lectin) 2 (hucolin) |
| FCN3 | ficolin (collagen/fibrinogen domain containing) 3 (Hakata antigen) |
| FCRL3 | Fc receptor-like 3 |
| FDX1 | ferredoxin 1 |
| FEN1 | flap structure-specific endonuclease 1 |
| FERMT1 | fermitin family member 1 |
| FERMT2 | fermitin family member 2 |
| FETUB | fetuin B |
| FEZ1 | fasciculation and elongation protein zeta 1 (zygin I) |
| FGA | fibrinogen alpha chain |
| FGB | fibrinogen beta chain |
| FGF13 | fibroblast growth factor 13 |
| FGF14 | fibroblast growth factor 14 |
| FGFR2 | fibroblast growth factor receptor 2 |
| FGL1 | fibrinogen-like 1 |
| FHL1 | four and a half LIM domains 1 |
| FIGN | fidgetin |
| FIGNL1 | fidgetin-like 1 |
| FILIP1L | filamin A interacting protein 1-like |
| FKBP11 | FK506 binding protein 11, 19 kDa |
| FKBP1B | FK506 binding protein 1B, 12.6 kDa |
| FLJ22763 | uncharacterized LOC401081 |
| FLJ38717 | FLJ38717 protein |
| FLJ39632 | uncharacterized LOC642477 |
| FLRT3 | fibronectin leucine rich transmembrane protein 3 |
| FLVCR1 | feline leukemia virus subgroup C cellular receptor 1 |
| FMO2 | flavin containing monooxygenase 2 (non-functional) |
| FMO3 | flavin containing monooxygenase 3 |
| FMO5 | flavin containing monooxygenase 5 |
| FNDC5 | fibronectin type III domain containing 5 |
| FNIP2 | folliculin interacting protein 2 |
| FOLH1 | folate hydrolase (prostate-specific membrane antigen) 1 |
| FOLH1B | folate hydrolase 1B |
| FOS | FBJ murine osteosarcoma viral oncogene homolog |
| FOSB | FBJ murine osteosarcoma viral oncogene homolog B |
| FOXA3 | forkhead box A3 |
| FOXM1 | forkhead box M1 |
| FOXO1 | forkhead box O1 |
| FOXP2 | forkhead box P2 |
| FREM2 | FRAS1 related extracellular matrix protein 2 |
| FRMD3 | FERM domain containing 3 |
| FRMD6 | FERM domain containing 6 |
| FSD1L | fibronectin type III and SPRY domain containing 1-like |
| FST | follistatin |
| FTCD | formiminotransferase cyclodeaminase |
| FUBP1 | far upstream element (FUSE) binding protein 1 |
| FXN | frataxin |
| FXYD1 | FXYD domain containing ion transport regulator 1 |
| FXYD2 | FXYD domain containing ion transport regulator 2 |
| FXYD6-FXYD2 | FXYD6-FXYD2 readthrough |
| FZD7 | frizzled family receptor 7 |
| G6PC | glucose-6-phosphatase, catalytic subunit |
| GABARAPL1 | GABA(A) receptor-associated protein like 1 |
| GABARAPL3 | GABA(A) receptors associated protein like 3, pseudogene |
| GABRB3 | gamma-aminobutyric acid (GABA) A receptor, beta 3 |
| GABRP | gamma-aminobutyric acid (GABA) A receptor, pi |
| GADD45A | growth arrest and DNA-damage-inducible, alpha |
| GADD45B | growth arrest and DNA-damage-inducible, beta |
| GADD45G | growth arrest and DNA-damage-inducible, gamma |
| GALNT10 | UDP-N-acetyl-alpha-D-galactosamine:polypeptide N-acetylgalactosaminyltransferase 10 (GalNAc-T10) |
| GALNT2 | UDP-N-acetyl-alpha-D-galactosamine:polypeptide N-acetylgalactosaminyltransferase 2 (GalNAc-T2) |
| GAMT | guanidinoacetate N-methyltransferase |
| GAREM | GRB2 associated, regulator of MAPK1 |
| GAS1 | growth arrest-specific 1 |
| GAS2L3 | growth arrest-specific 2 like 3 |
| GAS5 | growth arrest-specific 5 (non-protein coding) |
| GATA6 | GATA binding protein 6 |
| GATM | glycine amidinotransferase (L-arginine:glycine amidinotransferase) |
| GBA3 | glucosidase, beta, acid 3 (cytosolic) |
| GBP1 | guanylate binding protein 1, interferon-inducible |
| GCDH | glutaryl-CoA dehydrogenase |
| GCGR | glucagon receptor |
| GCH1 | GTP cyclohydrolase 1 |
| GCNT3 | glucosaminyl (N-acetyl) transferase 3, mucin type |
| GDA | guanine deaminase |
| GDAP1 | ganglioside induced differentiation associated protein 1 |
| GEM | GTP binding protein overexpressed in skeletal muscle |
| GFRA1 | GDNF family receptor alpha 1 |
| GGPS1 | geranylgeranyl diphosphate synthase 1 |
| GHR | growth hormone receptor |
| GINS1 | GINS complex subunit 1 (Psf1 homolog) |
| GIPC2 | GIPC PDZ domain containing family, member 2 |
| GJA1 | gap junction protein, alpha 1, 43kDa |
| GJB2 | gap junction protein, beta 2, 26kDa |
| GJC1 | gap junction protein, gamma 1, 45kDa |
| GK | glycerol kinase |
| GLDC | glycine dehydrogenase (decarboxylating) |
| GLIS3 | GLIS family zinc finger 3 |
| GLS2 | glutaminase 2 (liver, mitochondrial) |
| GLT1D1 | glycosyltransferase 1 domain containing 1 |
| GLUD1 | glutamate dehydrogenase 1 |
| GLUD2 | glutamate dehydrogenase 2 |
| GLUL | glutamate-ammonia ligase |
| GLYAT | glycine-N-acyltransferase |
| GLYATL1 | glycine-N-acyltransferase-like 1 |
| GM2A | GM2 ganglioside activator |
| GMDS | GDP-mannose 4,6-dehydratase |
| GNAI1 | guanine nucleotide binding protein (G protein), alpha inhibiting activity polypeptide 1 |
| GNAL | guanine nucleotide binding protein (G protein), alpha activating activity polypeptide, olfactory type |
| GNAO1 | guanine nucleotide binding protein (G protein), alpha activating activity polypeptide O |
| GNE | glucosamine (UDP-N-acetyl)-2-epimerase/N-acetylmannosamine kinase |
| GNMT | glycine N-methyltransferase |
| GNPNAT1 | glucosamine-phosphate N-acetyltransferase 1 |
| GOLM1 | golgi membrane protein 1 |
| GOLT1B | golgi transport 1B |
| GOT1 | glutamic-oxaloacetic transaminase 1, soluble (aspartate aminotransferase 1) |
| GOT2 | glutamic-oxaloacetic transaminase 2, mitochondrial (aspartate aminotransferase 2) |
| GPATCH2 | G patch domain containing 2 |
| GPC3 | glypican 3 |
| GPC6 | glypican 6 |
| GPD1 | glycerol-3-phosphate dehydrogenase 1 (soluble) |
| GPHN | gephyrin |
| GPLD1 | glycosylphosphatidylinositol specific phospholipase D1 |
| GPM6A | glycoprotein M6A |
| GPR125 | G protein-coupled receptor 125 |
| GPR126 | G protein-coupled receptor 126 |
| GPR128 | G protein-coupled receptor 128 |
| GPR158 | G protein-coupled receptor 158 |
| GPR171 | G protein-coupled receptor 171 |
| GPR180 | G protein-coupled receptor 180 |
| GPR182 | G protein-coupled receptor 182 |
| GPRASP1 | G protein-coupled receptor associated sorting protein 1 |
| GPT2 | glutamic pyruvate transaminase (alanine aminotransferase) 2 |
| GPX2 | glutathione peroxidase 2 (gastrointestinal) |
| GRAMD1C | GRAM domain containing 1C |
| GRAMD4 | GRAM domain containing 4 |
| GREB1 | growth regulation by estrogen in breast cancer 1 |
| GREM2 | gremlin 2 |
| GRHL1 | grainyhead-like 1 (Drosophila) |
| GRHPR | glyoxylate reductase/hydroxypyruvate reductase |
| GSTA1 | glutathione S-transferase alpha 1 |
| GSTA3 | glutathione S-transferase alpha 3 |
| GSTA4 | glutathione S-transferase alpha 4 |
| GSTZ1 | glutathione S-transferase zeta 1 |
| GULP1 | GULP, engulfment adaptor PTB domain containing 1 |
| GXYLT2 | glucoside xylosyltransferase 2 |
| GYS2 | glycogen synthase 2 (liver) |
| GZMK | granzyme K (granzyme 3; tryptase II) |
| H19 | H19, imprinted maternally expressed transcript (non-protein coding) |
| H2AFX | H2A histone family, member X |
| HAAO | 3-hydroxyanthranilate 3,4-dioxygenase |
| HABP2 | hyaluronan binding protein 2 |
| HAL | histidine ammonia-lyase |
| HAMP | hepcidin antimicrobial peptide |
| HAO1 | hydroxyacid oxidase (glycolate oxidase) 1 |
| HAO2 | hydroxyacid oxidase 2 (long chain) |
| HAUS6 | HAUS augmin-like complex, subunit 6 |
| HBA1/HBA2 | hemoglobin, alpha 1 |
| HBB | hemoglobin, beta |
| HDAC6 | histone deacetylase 6 |
| HELLS | helicase, lymphoid-specific |
| HGD | homogentisate 1,2-dioxygenase |
| HGF | hepatocyte growth factor (hepapoietin A; scatter factor) |
| HGFAC | HGF activator |
| HHIP | hedgehog interacting protein |
| HIGD1A | HIG1 hypoxia inducible domain family, member 1A |
| HILPDA | hypoxia inducible lipid droplet-associated |
| HIST1H2AC | histone cluster 1, H2ac |
| HIST1H3A | histone cluster 1, H3a |
| HIST2H2BE | histone cluster 2, H2be |
| HLF | hepatic leukemia factor |
| HMGB2 | high mobility group box 2 |
| HMGCS2 | 3-hydroxy-3-methylglutaryl-CoA synthase 2 (mitochondrial) |
| HMMR | hyaluronan-mediated motility receptor (RHAMM) |
| HMOX1 | heme oxygenase (decycling) 1 |
| HN1 | hematological and neurological expressed 1 |
| HOGA1 | 4-hydroxy-2-oxoglutarate aldolase 1 |
| HOOK1 | hook homolog 1 (Drosophila) |
| HOXA3 | homeobox A3 |
| HOXD8 | homeobox D8 |
| HPD | 4-hydroxyphenylpyruvate dioxygenase |
| HPGD | hydroxyprostaglandin dehydrogenase 15-(NAD) |
| HPN | hepsin |
| HPX | hemopexin |
| HRG | histidine-rich glycoprotein |
| HRSP12 | heat-responsive protein 12 |
| HS2ST1 | heparan sulfate 2-O-sulfotransferase 1 |
| HS3ST3B1 | heparan sulfate (glucosamine) 3-O-sulfotransferase 3B1 |
| HSD17B2 | hydroxysteroid (17-beta) dehydrogenase 2 |
| HSD17B6 | hydroxysteroid (17-beta) dehydrogenase 6 homolog (mouse) |
| HSPB1 | heat shock 27kDa protein 1 |
| HTATIP2 | HIV-1 Tat interactive protein 2, 30kDa |
| HYAL1 | hyaluronoglucosaminidase 1 |
| IAPP | islet amyloid polypeptide |
| ID1 | inhibitor of DNA binding 1, dominant negative helix-loop-helix protein |
| ID2 | inhibitor of DNA binding 2, dominant negative helix-loop-helix protein |
| ID4 | inhibitor of DNA binding 4, dominant negative helix-loop-helix protein |
| IDNK | idnK, gluconokinase homolog (E. coli) |
| IDO2 | indoleamine 2,3-dioxygenase 2 |
| IER2 | immediate early response 2 |
| IFI44 | interferon-induced protein 44 |
| IFITM10 | interferon induced transmembrane protein 10 |
| IFNLR1 | interferon, lambda receptor 1 |
| IFT80 | intraflagellar transport 80 homolog (Chlamydomonas) |
| IFT81 | intraflagellar transport 81 homolog (Chlamydomonas) |
| IGF1 | insulin-like growth factor 1 (somatomedin C) |
| IGF2 | insulin-like growth factor 2 (somatomedin A) |
| IGF2BP3 | insulin-like growth factor 2 mRNA binding protein 3 |
| IGFALS | insulin-like growth factor binding protein, acid labile subunit |
| IGFBP1 | insulin-like growth factor binding protein 1 |
| IGFBP3 | insulin-like growth factor binding protein 3 |
| IGHA1 | immunoglobulin heavy constant alpha 1 |
| IGHG1 | immunoglobulin heavy constant gamma 1 (G1m marker) |
| IGHM | immunoglobulin heavy constant mu |
| IGJ | immunoglobulin J polypeptide, linker protein for immunoglobulin alpha and mu polypeptides |
| IGKC | immunoglobulin kappa constant |
| IGLC1 | immunoglobulin lambda constant 1 (Mcg marker) |
| IGLL1/IGLL5 | immunoglobulin lambda-like polypeptide 1 |
| IGLL3P | immunoglobulin lambda-like polypeptide 3, pseudogene |
| IGSF3 | immunoglobulin superfamily, member 3 |
| IL13RA2 | interleukin 13 receptor, alpha 2 |
| IL18R1 | interleukin 18 receptor 1 |
| IL18RAP | interleukin 18 receptor accessory protein |
| IL1RAP | interleukin 1 receptor accessory protein |
| IL1RL1 | interleukin 1 receptor-like 1 |
| IL1RN | interleukin 1 receptor antagonist |
| IL33 | interleukin 33 |
| IL7R | interleukin 7 receptor |
| ILDR2 | immunoglobulin-like domain containing receptor 2 |
| INHBE | inhibin, beta E |
| INMT | indolethylamine N-methyltransferase |
| INSIG1 | insulin induced gene 1 |
| INTU | inturned planar cell polarity effector homolog (Drosophila) |
| IQGAP3 | IQ motif containing GTPase activating protein 3 |
| IRX3 | iroquois homeobox 3 |
| ISOC1 | isochorismatase domain containing 1 |
| ITCH | itchy E3 ubiquitin protein ligase |
| ITGA2 | integrin, alpha 2 (CD49B, alpha 2 subunit of VLA-2 receptor) |
| ITGA6 | integrin, alpha 6 |
| ITGA9 | integrin, alpha 9 |
| ITGB8 | integrin, beta 8 |
| ITIH1 | inter-alpha-trypsin inhibitor heavy chain 1 |
| ITIH2 | inter-alpha-trypsin inhibitor heavy chain 2 |
| ITIH3 | inter-alpha-trypsin inhibitor heavy chain 3 |
| ITIH4 | inter-alpha-trypsin inhibitor heavy chain family, member 4 |
| ITK | IL2-inducible T-cell kinase |
| ITSN1 | intersectin 1 (SH3 domain protein) |
| IVD | isovaleryl-CoA dehydrogenase |
| IYD | iodotyrosine deiodinase |
| JUN | jun proto-oncogene |
| JUNB | jun B proto-oncogene |
| KANK4 | KN motif and ankyrin repeat domains 4 |
| KAZN | kazrin, periplakin interacting protein |
| KBTBD11 | kelch repeat and BTB (POZ) domain containing 11 |
| KCND3 | potassium voltage-gated channel, Shal-related subfamily, member 3 |
| KCNE3 | potassium voltage-gated channel, Isk-related family, member 3 |
| KCNJ16 | potassium inwardly-rectifying channel, subfamily J, member 16 |
| KCNJ3 | potassium inwardly-rectifying channel, subfamily J, member 3 |
| KCNJ8 | potassium inwardly-rectifying channel, subfamily J, member 8 |
| KCNMA1 | potassium large conductance calcium-activated channel, subfamily M, alpha member 1 |
| KCNN2 | potassium intermediate/small conductance calcium-activated channel, subfamily N, member 2 |
| KDM8 | lysine (K)-specific demethylase 8 |
| KHK | ketohexokinase (fructokinase) |
| KIAA0101 | KIAA0101 |
| KIAA0146 | KIAA0146 |
| KIAA1199 | KIAA1199 |
| KIAA1244 | KIAA1244 |
| KIAA1462 | KIAA1462 |
| KIAA1919 | KIAA1919 |
| KIF11 | kinesin family member 11 |
| KIF14 | kinesin family member 14 |
| KIF20A | kinesin family member 20A |
| KIF26B | kinesin family member 26B |
| KIF3A | kinesin family member 3A |
| KIF4A | kinesin family member 4A |
| KITLG | KIT ligand |
| KLF10 | Kruppel-like factor 10 |
| KLF11 | Kruppel-like factor 11 |
| KLF4 | Kruppel-like factor 4 (gut) |
| KLF6 | Kruppel-like factor 6 |
| KLF9 | Kruppel-like factor 9 |
| KLHL15 | kelch-like 15 (Drosophila) |
| KLKB1 | kallikrein B, plasma (Fletcher factor) 1 |
| KLRB1 | killer cell lectin-like receptor subfamily B, member 1 |
| KLRC4-KLRK1/KLRK1 | killer cell lectin-like receptor subfamily K, member 1 |
| KLRF1 | killer cell lectin-like receptor subfamily F, member 1 |
| KMO | kynurenine 3-monooxygenase (kynurenine 3-hydroxylase) |
| KNG1 | kininogen 1 |
| KPNA2 | karyopherin alpha 2 (RAG cohort 1, importin alpha 1) |
| KRTCAP3 | keratinocyte associated protein 3 |
| L3MBTL1 | l(3)mbt-like 1 (Drosophila) |
| LAMA3 | laminin, alpha 3 |
| LAMA4 | laminin, alpha 4 |
| LAMC1 | laminin, gamma 1 (formerly LAMB2) |
| LARP1B | La ribonucleoprotein domain family, member 1B |
| LCAT | lecithin-cholesterol acyltransferase |
| LCN2 | lipocalin 2 |
| LDHD | lactate dehydrogenase D |
| LDLR | low density lipoprotein receptor |
| LEAP2 | liver expressed antimicrobial peptide 2 |
| LECT2 | leukocyte cell-derived chemotaxin 2 |
| LEF1 | lymphoid enhancer-binding factor 1 |
| LEPR | leptin receptor |
| LGR5 | leucine-rich repeat containing G protein-coupled receptor 5 |
| LGSN | lengsin, lens protein with glutamine synthetase domain |
| LHX2 | LIM homeobox 2 |
| LIFR | leukemia inhibitory factor receptor alpha |
| LIMK2 | LIM domain kinase 2 |
| LINC00094 | long intergenic non-protein coding RNA 94 |
| LINC00115 | long intergenic non-protein coding RNA 115 |
| LIPC | lipase, hepatic |
| LIPG | lipase, endothelial |
| LMOD1 | leiomodin 1 (smooth muscle) |
| LOC100128252 | uncharacterized LOC100128252 |
| LOC100129447 | uncharacterized LOC100129447 |
| LOC100132832/PMS2P5 | postmeiotic segregation increased 2 pseudogene 5 |
| LOC100132891 | uncharacterized LOC100132891 |
| LOC100134822 | uncharacterized LOC100134822 |
| LOC100216546 | uncharacterized LOC100216546 |
| LOC100289058 | uncharacterized LOC100289058 |
| LOC100505570 | uncharacterized LOC100505570 |
| LOC100505985 | uncharacterized LOC100505985 |
| LOC100506229 | uncharacterized LOC100506229 |
| LOC100507316 | uncharacterized LOC100507316 |
| LOC100507389 | uncharacterized LOC100507389 |
| LOC100507472 | uncharacterized LOC100507472 |
| LOC100507577 | uncharacterized LOC100507577 |
| LOC153682 | uncharacterized LOC153682 |
| LOC158402 | uncharacterized LOC158402 |
| LOC200772 | uncharacterized LOC200772 |
| LOC255167 | uncharacterized LOC255167 |
| LOC283587 | uncharacterized LOC283587 |
| LOC284801 | uncharacterized LOC284801 |
| LOC286087 | uncharacterized LOC286087 |
| LOC286114 | uncharacterized LOC286114 |
| LOC344887 | NmrA-like family domain containing 1 pseudogene |
| LOC389834 | ankyrin repeat domain 57 pseudogene |
| LOC400879 | uncharacterized LOC400879 |
| LOC401068 | uncharacterized LOC401068 |
| LOC401074 | uncharacterized LOC401074 |
| LOC441242 | uncharacterized LOC441242 |
| LOC541471 | uncharacterized LOC541471 |
| LOC642852 | uncharacterized LOC642852 |
| LOC692247 | uncharacterized LOC692247 |
| LONP2 | lon peptidase 2, peroxisomal |
| LONRF1 | LON peptidase N-terminal domain and ring finger 1 |
| LONRF2 | LON peptidase N-terminal domain and ring finger 2 |
| LONRF3 | LON peptidase N-terminal domain and ring finger 3 |
| LOX | lysyl oxidase |
| LOXL2 | lysyl oxidase-like 2 |
| LPA | lipoprotein, Lp(a) |
| LPCAT1 | lysophosphatidylcholine acyltransferase 1 |
| LPCAT2 | lysophosphatidylcholine acyltransferase 2 |
| LPIN2 | lipin 2 |
| LPL | lipoprotein lipase |
| LPPR1 | lipid phosphate phosphatase-related protein type 1 |
| LRG1 | leucine-rich alpha-2-glycoprotein 1 |
| LRIG2 | leucine-rich repeats and immunoglobulin-like domains 2 |
| LRP12 | low density lipoprotein receptor-related protein 12 |
| LRRC31 | leucine rich repeat containing 31 |
| LRRN3 | leucine rich repeat neuronal 3 |
| LSR | lipolysis stimulated lipoprotein receptor |
| LUM | lumican |
| LURAP1L | leucine rich adaptor protein 1-like |
| LY6E | lymphocyte antigen 6 complex, locus E |
| LY96 | lymphocyte antigen 96 |
| LYPD1 | LY6/PLAUR domain containing 1 |
| LYVE1 | lymphatic vessel endothelial hyaluronan receptor 1 |
| LYZ | lysozyme |
| MAD2L1 | MAD2 mitotic arrest deficient-like 1 (yeast) |
| MAGED4/MAGED4B | melanoma antigen family D, 4B |
| MALAT1 | metastasis associated lung adenocarcinoma transcript 1 (non-protein coding) |
| MAN1C1 | mannosidase, alpha, class 1C, member 1 |
| MAOA | monoamine oxidase A |
| MAP2 | microtubule-associated protein 2 |
| MAP2K1 | mitogen-activated protein kinase kinase 1 |
| MAP4K4 | mitogen-activated protein kinase kinase kinase kinase 4 |
| MARC1 | mitochondrial amidoxime reducing component 1 |
| MARC2 | mitochondrial amidoxime reducing component 2 |
| MARCKS | myristoylated alanine-rich protein kinase C substrate |
| MARCO | macrophage receptor with collagenous structure |
| MASP1 | mannan-binding lectin serine peptidase 1 (C4/C2 activating component of Ra-reactive factor) |
| MASP2 | mannan-binding lectin serine peptidase 2 |
| MAT1A | methionine adenosyltransferase I, alpha |
| MATR3 | matrin 3 |
| MBL2 | mannose-binding lectin (protein C) 2, soluble |
| MBNL2 | muscleblind-like splicing regulator 2 |
| MCC | mutated in colorectal cancers |
| MCL1 | myeloid cell leukemia sequence 1 (BCL2-related) |
| MCM2 | minichromosome maintenance complex component 2 |
| MCM6 | minichromosome maintenance complex component 6 |
| MCTP2 | multiple C2 domains, transmembrane 2 |
| MECOM | MDS1 and EVI1 complex locus |
| MELK | maternal embryonic leucine zipper kinase |
| MFAP3L | microfibrillar-associated protein 3-like |
| MFAP4 | microfibrillar-associated protein 4 |
| MFSD2A | major facilitator superfamily domain containing 2A |
| MGLL | monoglyceride lipase |
| MIF | macrophage migration inhibitory factor (glycosylation-inhibiting factor) |
| MIR17HG | miR-17-92 cluster host gene (non-protein coding) |
| MIR22HG | MIR22 host gene (non-protein coding) |
| MKI67 | antigen identified by monoclonal antibody Ki-67 |
| MLEC | malectin |
| MLF1IP | MLF1 interacting protein |
| MLIP | muscular LMNA-interacting protein |
| MLYCD | malonyl-CoA decarboxylase |
| MMAA | methylmalonic aciduria (cobalamin deficiency) cblA type |
| MMP12 | matrix metallopeptidase 12 (macrophage elastase) |
| MMP9 | matrix metallopeptidase 9 (gelatinase B, 92kDa gelatinase, 92kDa type IV collagenase) |
| MMRN1 | multimerin 1 |
| MNS1 | meiosis-specific nuclear structural 1 |
| MOGAT2 | monoacylglycerol O-acyltransferase 2 |
| MPC1 | mitochondrial pyruvate carrier 1 |
| MPDZ | multiple PDZ domain protein |
| MPEG1 | macrophage expressed 1 |
| MRAP2 | melanocortin 2 receptor accessory protein 2 |
| MRC1 | mannose receptor, C type 1 |
| MRGPRF | MAS-related GPR, member F |
| MRO | maestro |
| MRPS23 | mitochondrial ribosomal protein S23 |
| MS4A1 | membrane-spanning 4-domains, subfamily A, member 1 |
| MS4A7 | membrane-spanning 4-domains, subfamily A, member 7 |
| MSH2 | mutS homolog 2, colon cancer, nonpolyposis type 1 (E. coli) |
| MSH5 | mutS homolog 5 (E. coli) |
| MSMO1 | methylsterol monooxygenase 1 |
| MSRA | methionine sulfoxide reductase A |
| MST1 | macrophage stimulating 1 (hepatocyte growth factor-like) |
| MST1L | macrophage stimulating 1-like |
| MT1E | metallothionein 1E |
| MT1F | metallothionein 1F |
| MT1G | metallothionein 1G |
| MT1H | metallothionein 1H |
| MT1M | metallothionein 1M |
| MT1X | metallothionein 1X |
| MT2A | metallothionein 2A |
| MTHFD1 | methylenetetrahydrofolate dehydrogenase (NADP+ dependent) 1, methenyltetrahydrofolate cyclohydrolase, formyltetrahydrofolate synthetase |
| MTHFD1L | methylenetetrahydrofolate dehydrogenase (NADP+ dependent) 1-like |
| MTMR11 | myotubularin related protein 11 |
| MUC13 | mucin 13, cell surface associated |
| MUM1L1 | melanoma associated antigen (mutated) 1-like 1 |
| MUT | methylmalonyl CoA mutase |
| MXRA5 | matrix-remodelling associated 5 |
| MYC | v-myc myelocytomatosis viral oncogene homolog (avian) |
| MYEF2 | myelin expression factor 2 |
| MYH4 | myosin, heavy chain 4, skeletal muscle |
| MYO10 | myosin X |
| MYOM2 | myomesin 2 |
| N4BP2L1 | NEDD4 binding protein 2-like 1 |
| NAAA | N-acylethanolamine acid amidase |
| NADKD1 | NAD kinase domain containing 1 |
| NAMPT | nicotinamide phosphoribosyltransferase |
| NAPSB | napsin B aspartic peptidase, pseudogene |
| NAT1 | N-acetyltransferase 1 (arylamine N-acetyltransferase) |
| NAT2 | N-acetyltransferase 2 (arylamine N-acetyltransferase) |
| NAT8 | N-acetyltransferase 8 (GCN5-related, putative) |
| NAT8B | N-acetyltransferase 8B (GCN5-related, putative, gene/pseudogene) |
| NAV1 | neuron navigator 1 |
| NAV2 | neuron navigator 2 |
| NBLA00301 | Nbla00301 |
| NCAPD2 | non-SMC condensin I complex, subunit D2 |
| NCAPG | non-SMC condensin I complex, subunit G |
| NCOR1 | nuclear receptor corepressor 1 |
| NDC80 | NDC80 kinetochore complex component homolog (S. cerevisiae) |
| NDE1 | nudE nuclear distribution E homolog 1 (A. nidulans) |
| NDRG2 | NDRG family member 2 |
| NEB | nebulin |
| NEK2 | NIMA-related kinase 2 |
| NEU1 | sialidase 1 (lysosomal sialidase) |
| NFIA | nuclear factor I/A |
| NFIL3 | nuclear factor, interleukin 3 regulated |
| NIPAL1 | NIPA-like domain containing 1 |
| NLRP1 | NLR family, pyrin domain containing 1 |
| NME1 | NME/NM23 nucleoside diphosphate kinase 1 |
| NNMT | nicotinamide N-methyltransferase |
| NOV | nephroblastoma overexpressed |
| NPHP3 | nephronophthisis 3 (adolescent) |
| NPL | N-acetylneuraminate pyruvate lyase (dihydrodipicolinate synthase) |
| NPY1R | neuropeptide Y receptor Y1 |
| NQO1 | NAD(P)H dehydrogenase, quinone 1 |
| NR0B2 | nuclear receptor subfamily 0, group B, member 2 |
| NR1I2 | nuclear receptor subfamily 1, group I, member 2 |
| NR1I3 | nuclear receptor subfamily 1, group I, member 3 |
| NR3C2 | nuclear receptor subfamily 3, group C, member 2 |
| NR4A1 | nuclear receptor subfamily 4, group A, member 1 |
| NR4A2 | nuclear receptor subfamily 4, group A, member 2 |
| NR4A3 | nuclear receptor subfamily 4, group A, member 3 |
| NR5A2 | nuclear receptor subfamily 5, group A, member 2 |
| NRCAM | neuronal cell adhesion molecule |
| NRG1 | neuregulin 1 |
| NT5DC2 | 5'-nucleotidase domain containing 2 |
| NTM | neurotrimin |
| NTS | neurotensin |
| NUSAP1 | nucleolar and spindle associated protein 1 |
| NXF3 | nuclear RNA export factor 3 |
| OAT | ornithine aminotransferase |
| OGDHL | oxoglutarate dehydrogenase-like |
| OIT3 | oncoprotein induced transcript 3 |
| OLFML3 | olfactomedin-like 3 |
| ORM1 | orosomucoid 1 |
| OSBPL3 | oxysterol binding protein-like 3 |
| OTC | ornithine carbamoyltransferase |
| OVOS/OVOS2 | ovostatin 2 |
| P4HA1 | prolyl 4-hydroxylase, alpha polypeptide I |
| P4HA2 | prolyl 4-hydroxylase, alpha polypeptide II |
| PACRGL | PARK2 co-regulated-like |
| PAGE4 | P antigen family, member 4 (prostate associated) |
| PAH | phenylalanine hydroxylase |
| PAIP2B | poly(A) binding protein interacting protein 2B |
| PALLD | palladin, cytoskeletal associated protein |
| PALM2 | paralemmin 2 |
| PALM3 | paralemmin 3 |
| PAMR1 | peptidase domain containing associated with muscle regeneration 1 |
| PANK1 | pantothenate kinase 1 |
| PAPSS2 | 3'-phosphoadenosine 5'-phosphosulfate synthase 2 |
| PBK | PDZ binding kinase |
| PBLD | phenazine biosynthesis-like protein domain containing |
| PCDH17 | protocadherin 17 |
| PCDH9 | protocadherin 9 |
| PCK1 | phosphoenolpyruvate carboxykinase 1 (soluble) |
| PCK2 | phosphoenolpyruvate carboxykinase 2 (mitochondrial) |
| PCOLCE | procollagen C-endopeptidase enhancer |
| PCSK5 | proprotein convertase subtilisin/kexin type 5 |
| PCSK6 | proprotein convertase subtilisin/kexin type 6 |
| PDE3B | phosphodiesterase 3B, cGMP-inhibited |
| PDE7B | phosphodiesterase 7B |
| PDGFRA | platelet-derived growth factor receptor, alpha polypeptide |
| PDK4 | pyruvate dehydrogenase kinase, isozyme 4 |
| PDLIM5 | PDZ and LIM domain 5 |
| PEG10 | paternally expressed 10 |
| PEG3 | paternally expressed 3 |
| PEMT | phosphatidylethanolamine N-methyltransferase |
| PER1 | period circadian clock 1 |
| PFKFB1 | 6-phosphofructo-2-kinase/fructose-2,6-biphosphatase 1 |
| PFKFB2 | 6-phosphofructo-2-kinase/fructose-2,6-biphosphatase 2 |
| PFKFB3 | 6-phosphofructo-2-kinase/fructose-2,6-biphosphatase 3 |
| PGLYRP2 | peptidoglycan recognition protein 2 |
| PHGDH | phosphoglycerate dehydrogenase |
| PHLDA1 | pleckstrin homology-like domain, family A, member 1 |
| PHYH | phytanoyl-CoA 2-hydroxylase |
| PI15 | peptidase inhibitor 15 |
| PIEZO2 | piezo-type mechanosensitive ion channel component 2 |
| PIK3C2G | phosphatidylinositol-4-phosphate 3-kinase, catalytic subunit type 2 gamma |
| PIK3R1 | phosphoinositide-3-kinase, regulatory subunit 1 (alpha) |
| PIPOX | pipecolic acid oxidase |
| PIR | pirin (iron-binding nuclear protein) |
| PITPNM3 | PITPNM family member 3 |
| PKHD1 | polycystic kidney and hepatic disease 1 (autosomal recessive) |
| PKM | pyruvate kinase, muscle |
| PLA2G12B | phospholipase A2, group XIIB |
| PLAC8 | placenta-specific 8 |
| PLAG1 | pleiomorphic adenoma gene 1 |
| PLAU | plasminogen activator, urokinase |
| PLCE1 | phospholipase C, epsilon 1 |
| PLCXD3 | phosphatidylinositol-specific phospholipase C, X domain containing 3 |
| PLD1 | phospholipase D1, phosphatidylcholine-specific |
| PLG | plasminogen |
| PLGLB1/PLGLB2 | plasminogen-like B2 |
| PLIN1 | perilipin 1 |
| PLIN2 | perilipin 2 |
| PLSCR4 | phospholipid scramblase 4 |
| PMEPA1 | prostate transmembrane protein, androgen induced 1 |
| PODXL | podocalyxin-like |
| POLR2J2/POLR2J3 | polymerase (RNA) II (DNA directed) polypeptide J3 |
| PON1 | paraoxonase 1 |
| PON3 | paraoxonase 3 |
| POR | P450 (cytochrome) oxidoreductase |
| POU2AF1 | POU class 2 associating factor 1 |
| PPAP2B | phosphatidic acid phosphatase type 2B |
| PPARGC-1α/PGC-1α | peroxisome proliferator-activated receptor gamma, coactivator 1 alpha |
| PPID | peptidylprolyl isomerase D |
| PPP1R1A | protein phosphatase 1, regulatory (inhibitor) subunit 1A |
| PPP1R3B | protein phosphatase 1, regulatory subunit 3B |
| PPP2R1B | protein phosphatase 2, regulatory subunit A, beta |
| PRC1 | protein regulator of cytokinesis 1 |
| PRELP | proline/arginine-rich end leucine-rich repeat protein |
| PRG4 | proteoglycan 4 |
| PRIM1 | primase, DNA, polypeptide 1 (49kDa) |
| PRKAA2 | protein kinase, AMP-activated, alpha 2 catalytic subunit |
| PRKAG2 | protein kinase, AMP-activated, gamma 2 non-catalytic subunit |
| PRKAR2B | protein kinase, cAMP-dependent, regulatory, type II, beta |
| PRKD1 | protein kinase D1 |
| PRKDC | protein kinase, DNA-activated, catalytic polypeptide |
| PROC | protein C (inactivator of coagulation factors Va and VIIIa) |
| PRODH2 | proline dehydrogenase (oxidase) 2 |
| PROSER2 | proline and serine-rich protein 2 |
| PROZ | protein Z, vitamin K-dependent plasma glycoprotein |
| PRR11 | proline rich 11 |
| PRR18 | proline rich 18 |
| PRR26 | proline rich 26 |
| PRR5L | proline rich 5 like |
| PRRG4 | proline rich Gla (G-carboxyglutamic acid) 4 (transmembrane) |
| PSAT1 | phosphoserine aminotransferase 1 |
| PSD3 | pleckstrin and Sec7 domain containing 3 |
| PSMA5 | proteasome (prosome, macropain) subunit, alpha type, 5 |
| PSPH | phosphoserine phosphatase |
| PTBP3 | polypyrimidine tract binding protein 3 |
| PTGFRN | prostaglandin F2 receptor negative regulator |
| PTGIS | prostaglandin I2 (prostacyclin) synthase |
| PTGR1 | prostaglandin reductase 1 |
| PTGS2 | prostaglandin-endoperoxide synthase 2 (prostaglandin G/H synthase and cyclooxygenase) |
| PTH1R | parathyroid hormone 1 receptor |
| PTK2 | PTK2 protein tyrosine kinase 2 |
| PTN | pleiotrophin |
| PTP4A1 | protein tyrosine phosphatase type IVA, member 1 |
| PTP4A3 | protein tyrosine phosphatase type IVA, member 3 |
| PTPDC1 | protein tyrosine phosphatase domain containing 1 |
| PTPRB | protein tyrosine phosphatase, receptor type, B |
| PTPRG | protein tyrosine phosphatase, receptor type, G |
| PTPRS | protein tyrosine phosphatase, receptor type, S |
| PTTG1 | pituitary tumor-transforming 1 |
| PUS10 | pseudouridylate synthase 10 |
| PUS7 | pseudouridylate synthase 7 homolog (S. cerevisiae) |
| PXDC1 | PX domain containing 1 |
| PXMP2 | peroxisomal membrane protein 2, 22kDa |
| PYGL | phosphorylase, glycogen, liver |
| PZP | pregnancy-zone protein |
| QDPR | quinoid dihydropteridine reductase |
| RAB17 | RAB17, member RAS oncogene family |
| RAB23 | RAB23, member RAS oncogene family |
| RAB25 | RAB25, member RAS oncogene family |
| RAB26 | RAB26, member RAS oncogene family |
| RAB27A | RAB27A, member RAS oncogene family |
| RAB3B | RAB3B, member RAS oncogene family |
| RACGAP1 | Rac GTPase activating protein 1 |
| RAD21 | RAD21 homolog (S. pombe) |
| RAD51AP1 | RAD51 associated protein 1 |
| RAD54B | RAD54 homolog B (S. cerevisiae) |
| RAPH1 | Ras association (RalGDS/AF-6) and pleckstrin homology domains 1 |
| RARRES1 | retinoic acid receptor responder (tazarotene induced) 1 |
| RASAL2 | RAS protein activator like 2 |
| RASGEF1B | RasGEF domain family, member 1B |
| RASSF4 | Ras association (RalGDS/AF-6) domain family member 4 |
| RBM15 | RNA binding motif protein 15 |
| RBM24 | RNA binding motif protein 24 |
| RBMS3 | RNA binding motif, single stranded interacting protein 3 |
| RBP5 | retinol binding protein 5, cellular |
| RCAN1 | regulator of calcineurin 1 |
| RCL1 | RNA terminal phosphate cyclase-like 1 |
| RDH16 | retinol dehydrogenase 16 (all-trans) |
| RDH5 | retinol dehydrogenase 5 (11-cis/9-cis) |
| REEP6 | receptor accessory protein 6 |
| REG3A | regenerating islet-derived 3 alpha |
| REPS2 | RALBP1 associated Eps domain containing 2 |
| RFX5 | regulatory factor X, 5 (influences HLA class II expression) |
| RGN | regucalcin (senescence marker protein-30) |
| RGS4 | regulator of G-protein signaling 4 |
| RGS5 | regulator of G-protein signaling 5 |
| RHBG | Rh family, B glycoprotein (gene/pseudogene) |
| RHEB | Ras homolog enriched in brain |
| RHOB | ras homolog family member B |
| RHOBTB1 | Rho-related BTB domain containing 1 |
| RHOU | ras homolog family member U |
| RIPK4 | receptor-interacting serine-threonine kinase 4 |
| RMST | rhabdomyosarcoma 2 associated transcript (non-protein coding) |
| RNASE4 | ribonuclease, RNase A family, 4 |
| RND1 | Rho family GTPase 1 |
| RND3 | Rho family GTPase 3 |
| RNF125 | ring finger protein 125, E3 ubiquitin protein ligase |
| RNF165 | ring finger protein 165 |
| RNF180 | ring finger protein 180 |
| RNF213 | ring finger protein 213 |
| RNF43 | ring finger protein 43 |
| ROBO1 | roundabout, axon guidance receptor, homolog 1 (Drosophila) |
| RORA | RAR-related orphan receptor A |
| RPS27 | ribosomal protein S27 |
| RRAGD | Ras-related GTP binding D |
| RRM2 | ribonucleotide reductase M2 |
| RSPO3 | R-spondin 3 |
| RUNX1 | runt-related transcription factor 1 |
| S100A10 | S100 calcium binding protein A10 |
| S100P | S100 calcium binding protein P |
| S1PR3 | sphingosine-1-phosphate receptor 3 |
| SAA2-SAA4/SAA4 | serum amyloid A4, constitutive |
| SAC3D1 | SAC3 domain containing 1 |
| SACS | spastic ataxia of Charlevoix-Saguenay (sacsin) |
| SALL1 | sal-like 1 (Drosophila) |
| SAMD4A | sterile alpha motif domain containing 4A |
| SAMD5 | sterile alpha motif domain containing 5 |
| SARDH | sarcosine dehydrogenase |
| SATB1 | SATB homeobox 1 |
| SBSPON | somatomedin B and thrombospondin, type 1 domain containing |
| SC5DL | sterol-C5-desaturase (ERG3 delta-5-desaturase homolog, S. cerevisiae)-like |
| SCARA5 | scavenger receptor class A, member 5 (putative) |
| SCARNA17 | small Cajal body-specific RNA 17 |
| SCD5 | stearoyl-CoA desaturase 5 |
| SCN7A | sodium channel, voltage-gated, type VII, alpha subunit |
| SCNN1A | sodium channel, non-voltage-gated 1 alpha subunit |
| SCPEP1 | serine carboxypeptidase 1 |
| SCRN1 | secernin 1 |
| SDCBP2-AS1 | SDCBP2 antisense RNA 1 |
| SDPR | serum deprivation response |
| SDS | serine dehydratase |
| SDSL | serine dehydratase-like |
| SEC14L2 | SEC14-like 2 (S. cerevisiae) |
| SEC14L4 | SEC14-like 4 (S. cerevisiae) |
| SELP | selectin P (granule membrane protein 140kDa, antigen CD62) |
| SEMA6D | sema domain, transmembrane domain (TM), and cytoplasmic domain, (semaphorin) 6D |
| SEPP1 | selenoprotein P, plasma, 1 |
| SERPINA10 | serpin peptidase inhibitor, clade A (alpha-1 antiproteinase, antitrypsin), member 10 |
| SERPINA3 | serpin peptidase inhibitor, clade A (alpha-1 antiproteinase, antitrypsin), member 3 |
| SERPINA4 | serpin peptidase inhibitor, clade A (alpha-1 antiproteinase, antitrypsin), member 4 |
| SERPINA5 | serpin peptidase inhibitor, clade A (alpha-1 antiproteinase, antitrypsin), member 5 |
| SERPINA6 | serpin peptidase inhibitor, clade A (alpha-1 antiproteinase, antitrypsin), member 6 |
| SERPINA7 | serpin peptidase inhibitor, clade A (alpha-1 antiproteinase, antitrypsin), member 7 |
| SERPINB9 | serpin peptidase inhibitor, clade B (ovalbumin), member 9 |
| SERPINE1 | serpin peptidase inhibitor, clade E (nexin, plasminogen activator inhibitor type 1), member 1 |
| SERPINE2 | serpin peptidase inhibitor, clade E (nexin, plasminogen activator inhibitor type 1), member 2 |
| SERPINF1 | serpin peptidase inhibitor, clade F (alpha-2 antiplasmin, pigment epithelium derived factor), member 1 |
| SERPINF2 | serpin peptidase inhibitor, clade F (alpha-2 antiplasmin, pigment epithelium derived factor), member 2 |
| SERPINI1 | serpin peptidase inhibitor, clade I (neuroserpin), member 1 |
| SFN | stratifin |
| SGK1 | serum/glucocorticoid regulated kinase 1 |
| SGOL2 | shugoshin-like 2 (S. pombe) |
| SH3RF2 | SH3 domain containing ring finger 2 |
| SH3YL1 | SH3 domain containing, Ysc84-like 1 (S. cerevisiae) |
| SHBG | sex hormone-binding globulin |
| SHMT1 | serine hydroxymethyltransferase 1 (soluble) |
| SHROOM2 | shroom family member 2 |
| SIK1 | salt-inducible kinase 1 |
| SKAP1 | src kinase associated phosphoprotein 1 |
| SKAP2 | src kinase associated phosphoprotein 2 |
| SLC10A1 | solute carrier family 10 (sodium/bile acid cotransporter family), member 1 |
| SLC12A1 | solute carrier family 12 (sodium/potassium/chloride transporters), member 1 |
| SLC13A5 | solute carrier family 13 (sodium-dependent citrate transporter), member 5 |
| SLC15A1 | solute carrier family 15 (oligopeptide transporter), member 1 |
| SLC16A2 | solute carrier family 16, member 2 (thyroid hormone transporter) |
| SLC16A3 | solute carrier family 16, member 3 (monocarboxylic acid transporter 4) |
| SLC17A1 | solute carrier family 17 (sodium phosphate), member 1 |
| SLC17A2 | solute carrier family 17 (sodium phosphate), member 2 |
| SLC19A3 | solute carrier family 19, member 3 |
| SLC1A1 | solute carrier family 1 (neuronal/epithelial high affinity glutamate transporter, system Xag), member 1 |
| SLC1A3 | solute carrier family 1 (glial high affinity glutamate transporter), member 3 |
| SLC20A1 | solute carrier family 20 (phosphate transporter), member 1 |
| SLC22A1 | solute carrier family 22 (organic cation transporter), member 1 |
| SLC22A11 | solute carrier family 22 (organic anion/urate transporter), member 11 |
| SLC22A25 | solute carrier family 22, member 25 |
| SLC22A4 | solute carrier family 22 (organic cation/ergothioneine transporter), member 4 |
| SLC22A7 | solute carrier family 22 (organic anion transporter), member 7 |
| SLC23A2 | solute carrier family 23 (nucleobase transporters), member 2 |
| SLC25A15 | solute carrier family 25 (mitochondrial carrier; ornithine transporter) member 15 |
| SLC25A16 | solute carrier family 25 (mitochondrial carrier; Graves disease autoantigen), member 16 |
| SLC25A18 | solute carrier family 25 (glutamate carrier), member 18 |
| SLC25A20 | solute carrier family 25 (carnitine/acylcarnitine translocase), member 20 |
| SLC25A25 | solute carrier family 25 (mitochondrial carrier; phosphate carrier), member 25 |
| SLC25A27 | solute carrier family 25, member 27 |
| SLC25A47 | solute carrier family 25, member 47 |
| SLC26A2 | solute carrier family 26 (sulfate transporter), member 2 |
| SLC27A2 | solute carrier family 27 (fatty acid transporter), member 2 |
| SLC27A5 | solute carrier family 27 (fatty acid transporter), member 5 |
| SLC28A1 | solute carrier family 28 (sodium-coupled nucleoside transporter), member 1 |
| SLC28A3 | solute carrier family 28 (sodium-coupled nucleoside transporter), member 3 |
| SLC2A2 | solute carrier family 2 (facilitated glucose transporter), member 2 |
| SLC2A5 | solute carrier family 2 (facilitated glucose/fructose transporter), member 5 |
| SLC2A9 | solute carrier family 2 (facilitated glucose transporter), member 9 |
| SLC31A1 | solute carrier family 31 (copper transporters), member 1 |
| SLC35D1 | solute carrier family 35 (UDP-glucuronic acid/UDP-N-acetylgalactosamine dual transporter), member D1 |
| SLC37A4 | solute carrier family 37 (glucose-6-phosphate transporter), member 4 |
| SLC38A2 | solute carrier family 38, member 2 |
| SLC38A3 | solute carrier family 38, member 3 |
| SLC38A4 | solute carrier family 38, member 4 |
| SLC38A6 | solute carrier family 38, member 6 |
| SLC39A10 | solute carrier family 39 (zinc transporter), member 10 |
| SLC39A5 | solute carrier family 39 (metal ion transporter), member 5 |
| SLC3A1 | solute carrier family 3 (cystine, dibasic and neutral amino acid transporters, activator of cystine, dibasic and neutral amino acid transport), member 1 |
| SLC41A2 | solute carrier family 41, member 2 |
| SLC44A1 | solute carrier family 44, member 1 |
| SLC44A5 | solute carrier family 44, member 5 |
| SLC4A4 | solute carrier family 4, sodium bicarbonate cotransporter, member 4 |
| SLC51A | solute carrier family 51, alpha subunit |
| SLC52A2 | solute carrier family 52, riboflavin transporter, member 2 |
| SLC6A1 | solute carrier family 6 (neurotransmitter transporter, GABA), member 1 |
| SLC6A12 | solute carrier family 6 (neurotransmitter transporter, betaine/GABA), member 12 |
| SLC6A8 | solute carrier family 6 (neurotransmitter transporter, creatine), member 8 |
| SLC7A11 | solute carrier family 7 (anionic amino acid transporter light chain, xc- system), member 11 |
| SLC7A2 | solute carrier family 7 (cationic amino acid transporter, y+ system), member 2 |
| SLC7A6 | solute carrier family 7 (amino acid transporter light chain, y+L system), member 6 |
| SLC9B2 | solute carrier family 9, subfamily B (NHA2, cation proton antiporter 2), member 2 |
| SLCO1B1 | solute carrier organic anion transporter family, member 1B1 |
| SLCO1B3 | solute carrier organic anion transporter family, member 1B3 |
| SLCO4C1 | solute carrier organic anion transporter family, member 4C1 |
| SLPI | secretory leukocyte peptidase inhibitor |
| SMAD9 | SMAD family member 9 |
| SMC2 | structural maintenance of chromosomes 2 |
| SMOC1 | SPARC related modular calcium binding 1 |
| SMPX | small muscle protein, X-linked |
| SOCS2 | suppressor of cytokine signaling 2 |
| SOCS3 | suppressor of cytokine signaling 3 |
| SORBS1 | sorbin and SH3 domain containing 1 |
| SORBS2 | sorbin and SH3 domain containing 2 |
| SORD | sorbitol dehydrogenase |
| SORL1 | sortilin-related receptor, L(DLR class) A repeats containing |
| SOX7 | SRY (sex determining region Y)-box 7 |
| SP5 | Sp5 transcription factor |
| SPA17 | sperm autoantigenic protein 17 |
| SPATA18 | spermatogenesis associated 18 |
| SPATA5 | spermatogenesis associated 5 |
| SPATA6L | spermatogenesis associated 6-like |
| SPATS2 | spermatogenesis associated, serine-rich 2 |
| SPDL1 | spindle apparatus coiled-coil protein 1 |
| SPIN3 | spindlin family, member 3 |
| SPINK1 | serine peptidase inhibitor, Kazal type 1 |
| SPINT2 | serine peptidase inhibitor, Kunitz type, 2 |
| SPON2 | spondin 2, extracellular matrix protein |
| SPP1 | secreted phosphoprotein 1 |
| SPP2 | secreted phosphoprotein 2, 24kDa |
| SPRYD4 | SPRY domain containing 4 |
| SQLE | squalene epoxidase |
| SQSTM1 | sequestosome 1 |
| SRD5A1 | steroid-5-alpha-reductase, alpha polypeptide 1 (3-oxo-5 alpha-steroid delta 4-dehydrogenase alpha 1) |
| SRD5A2 | steroid-5-alpha-reductase, alpha polypeptide 2 (3-oxo-5 alpha-steroid delta 4-dehydrogenase alpha 2) |
| SRGAP2 | SLIT-ROBO Rho GTPase activating protein 2 |
| SRGAP2B | SLIT-ROBO Rho GTPase activating protein 2B (pseudogene) |
| SRGAP2C | SLIT-ROBO Rho GTPase activating protein 2C |
| SRPX | sushi-repeat containing protein, X-linked |
| SRXN1 | sulfiredoxin 1 |
| ST20 | suppressor of tumorigenicity 20 |
| ST3GAL6 | ST3 beta-galactoside alpha-2,3-sialyltransferase 6 |
| ST6GAL1 | ST6 beta-galactosamide alpha-2,6-sialyltranferase 1 |
| ST6GAL2 | ST6 beta-galactosamide alpha-2,6-sialyltranferase 2 |
| STAB2 | stabilin 2 |
| STARD5 | StAR-related lipid transfer (START) domain containing 5 |
| STAT4 | signal transducer and activator of transcription 4 |
| STEAP1 | six transmembrane epithelial antigen of the prostate 1 |
| STEAP2 | STEAP family member 2, metalloreductase |
| STEAP3 | STEAP family member 3, metalloreductase |
| STEAP4 | STEAP family member 4 |
| STK39 | serine threonine kinase 39 |
| STMN1 | stathmin 1 |
| STXBP6 | syntaxin binding protein 6 (amisyn) |
| SUCLG2 | succinate-CoA ligase, GDP-forming, beta subunit |
| SUCO | SUN domain containing ossification factor |
| SULT1A1 | sulfotransferase family, cytosolic, 1A, phenol-preferring, member 1 |
| SULT1A2 | sulfotransferase family, cytosolic, 1A, phenol-preferring, member 2 |
| SULT1C2 | sulfotransferase family, cytosolic, 1C, member 2 |
| SULT2A1 | sulfotransferase family, cytosolic, 2A, dehydroepiandrosterone (DHEA)-preferring, member 1 |
| SUZ12 | suppressor of zeste 12 homolog (Drosophila) |
| SYNE1 | spectrin repeat containing, nuclear envelope 1 |
| SYNPO2 | synaptopodin 2 |
| SYT17 | synaptotagmin XVII |
| SYT9 | synaptotagmin IX |
| SYTL3 | synaptotagmin-like 3 |
| TACSTD2 | tumor-associated calcium signal transducer 2 |
| TAF1A | TATA box binding protein (TBP)-associated factor, RNA polymerase I, A, 48kDa |
| TAGLN2 | transgelin 2 |
| TAPT1-AS1 | TAPT1 antisense RNA 1 (head to head) |
| TARP | TCR gamma alternate reading frame protein |
| TAT | tyrosine aminotransferase |
| TAX1BP3 | Tax1 (human T-cell leukemia virus type I) binding protein 3 |
| TBC1D16 | TBC1 domain family, member 16 |
| TBL1XR1 | transducin (beta)-like 1 X-linked receptor 1 |
| TBX15 | T-box 15 |
| TBXA2R | thromboxane A2 receptor |
| TCEA2 | transcription elongation factor A (SII), 2 |
| TCF21 | transcription factor 21 |
| TCTEX1D1 | Tctex1 domain containing 1 |
| TDO2 | tryptophan 2,3-dioxygenase |
| TDRD6 | tudor domain containing 6 |
| TEK | TEK tyrosine kinase, endothelial |
| TENM1 | teneurin transmembrane protein 1 |
| TF | transferrin |
| TFPI | tissue factor pathway inhibitor (lipoprotein-associated coagulation inhibitor) |
| TFPI2 | tissue factor pathway inhibitor 2 |
| TFR2 | transferrin receptor 2 |
| TGFA | transforming growth factor, alpha |
| TGFB2 | transforming growth factor, beta 2 |
| THBD | thrombomodulin |
| THBS1 | thrombospondin 1 |
| THRSP | thyroid hormone responsive |
| THSD7A | thrombospondin, type I, domain containing 7A |
| THY1 | Thy-1 cell surface antigen |
| TIAM1 | T-cell lymphoma invasion and metastasis 1 |
| TIGD1 | tigger transposable element derived 1 |
| TIGD2 | tigger transposable element derived 2 |
| TIGD7 | tigger transposable element derived 7 |
| TIMD4 | T-cell immunoglobulin and mucin domain containing 4 |
| TIPARP | TCDD-inducible poly(ADP-ribose) polymerase |
| TJP2 | tight junction protein 2 |
| TK1 | thymidine kinase 1, soluble |
| TKT | transketolase |
| TMC5 | transmembrane channel-like 5 |
| TMEM164 | transmembrane protein 164 |
| TMEM178A | transmembrane protein 178A |
| TMEM178B | transmembrane protein 178B |
| TMEM200C | transmembrane protein 200C |
| TMEM220 | transmembrane protein 220 |
| TMEM27 | transmembrane protein 27 |
| TMEM30B | transmembrane protein 30B |
| TMEM45A | transmembrane protein 45A |
| TMEM45B | transmembrane protein 45B |
| TMEM56 | transmembrane protein 56 |
| TMEM64 | transmembrane protein 64 |
| TMPRSS2 | transmembrane protease, serine 2 |
| TMPRSS6 | transmembrane protease, serine 6 |
| TNFRSF10D | tumor necrosis factor receptor superfamily, member 10d, decoy with truncated death domain |
| TNFRSF17 | tumor necrosis factor receptor superfamily, member 17 |
| TNFRSF19 | tumor necrosis factor receptor superfamily, member 19 |
| TNFSF15 | tumor necrosis factor (ligand) superfamily, member 15 |
| TNFSF4 | tumor necrosis factor (ligand) superfamily, member 4 |
| TOMM40L | translocase of outer mitochondrial membrane 40 homolog (yeast)-like |
| TOP2A | topoisomerase (DNA) II alpha 170kDa |
| TOX3 | TOX high mobility group box family member 3 |
| TP53I3 | tumor protein p53 inducible protein 3 |
| TPM2 | tropomyosin 2 (beta) |
| TPR | translocated promoter region, nuclear basket protein |
| TPX2 | TPX2, microtubule-associated, homolog (Xenopus laevis) |
| TRAF5 | TNF receptor-associated factor 5 |
| TRIB1 | tribbles homolog 1 (Drosophila) |
| TRIM16 | tripartite motif containing 16 |
| TRIM22 | tripartite motif containing 22 |
| TRIM45 | tripartite motif containing 45 |
| TRIM55 | tripartite motif containing 55 |
| TRIM59 | tripartite motif containing 59 |
| TRIM6 | tripartite motif containing 6 |
| TRPM8 | transient receptor potential cation channel, subfamily M, member 8 |
| TRPS1 | trichorhinophalangeal syndrome I |
| TSLP | thymic stromal lymphopoietin |
| TSPAN5 | tetraspanin 5 |
| TSPYL5 | TSPY-like 5 |
| TTC36 | tetratricopeptide repeat domain 36 |
| TTC39A | tetratricopeptide repeat domain 39A |
| TTC39C | tetratricopeptide repeat domain 39C |
| TTLL7 | tubulin tyrosine ligase-like family, member 7 |
| TTR | transthyretin |
| TUBA1B | tubulin, alpha 1b |
| TUBA4A | tubulin, alpha 4a |
| TUBE1 | tubulin, epsilon 1 |
| TUBG1 | tubulin, gamma 1 |
| TULP3 | tubby like protein 3 |
| TXN | thioredoxin |
| TXNL1 | thioredoxin-like 1 |
| TXNRD1 | thioredoxin reductase 1 |
| TYMS | thymidylate synthetase |
| UAP1 | UDP-N-acteylglucosamine pyrophosphorylase 1 |
| UGDH | UDP-glucose 6-dehydrogenase |
| UGGT1 | UDP-glucose glycoprotein glucosyltransferase 1 |
| UGP2 | UDP-glucose pyrophosphorylase 2 |
| UGT1A6 | UDP glucuronosyltransferase 1 family, polypeptide A6 |
| UGT2B15 | UDP glucuronosyltransferase 2 family, polypeptide B15 |
| UGT3A1 | UDP glycosyltransferase 3 family, polypeptide A1 |
| UHRF1 | ubiquitin-like with PHD and ring finger domains 1 |
| UNC93A | unc-93 homolog A (C. elegans) |
| UNKL | unkempt homolog (Drosophila)-like |
| UPB1 | ureidopropionase, beta |
| UPP2 | uridine phosphorylase 2 |
| USH1C | Usher syndrome 1C (autosomal recessive, severe) |
| USO1 | USO1 vesicle transport factor |
| USP2 | ubiquitin specific peptidase 2 |
| USP6NL | USP6 N-terminal like |
| VASH2 | vasohibin 2 |
| VCAN | versican |
| VIPR1 | vasoactive intestinal peptide receptor 1 |
| VLDLR | very low density lipoprotein receptor |
| VNN1 | vanin 1 |
| VNN2 | vanin 2 |
| VNN3 | vanin 3 |
| VTN | vitronectin |
| WASF3 | WAS protein family, member 3 |
| WDR72 | WD repeat domain 72 |
| WNT5A | wingless-type MMTV integration site family, member 5A |
| WWC1 | WW and C2 domain containing 1 |
| XDH | xanthine dehydrogenase |
| XK | X-linked Kx blood group (McLeod syndrome) |
| YPEL2 | yippee-like 2 (Drosophila) |
| ZBTB16 | zinc finger and BTB domain containing 16 |
| ZBTB26 | zinc finger and BTB domain containing 26 |
| ZBTB41 | zinc finger and BTB domain containing 41 |
| ZC2HC1C | zinc finger, C2HC-type containing 1C |
| ZDHHC11 | zinc finger, DHHC-type containing 11 |
| ZFP1 | ZFP1 zinc finger protein |
| ZFP3 | ZFP3 zinc finger protein |
| ZFP36 | ZFP36 ring finger protein |
| ZFPM2 | zinc finger protein, FOG family member 2 |
| ZG16 | zymogen granule protein 16 |
| ZGPAT | zinc finger, CCCH-type with G patch domain |
| ZIC1 | Zic family member 1 |
| ZIC2 | Zic family member 2 |
| ZKSCAN3 | zinc finger with KRAB and SCAN domains 3 |
| ZNF107 | zinc finger protein 107 |
| ZNF280C | zinc finger protein 280C |
| ZNF295 | zinc finger protein 295 |
| ZNF623 | zinc finger protein 623 |
| ZNF652 | zinc finger protein 652 |
| ZNF703 | zinc finger protein 703 |
| ZNF738 | zinc finger protein 738 |
| ZWINT | ZW10 interactor |
