# Supplementary material for: Viral expression and molecular profiling in liver tissue versus microdissected hepatocytes in hepatitis B virus - associated hepatocellular carcinoma
Source: J Transl Med. 2014 Aug 21;12:230. doi: 10.1186/s12967-014-0230-1 (PMC4142136; doi:10.1186/s12967-014-0230-1)
Supplement: Additional file 3: Table S2. — Genes Differentially Expressed between Microdissected Malignant Hepatocytes and Non-Malignant Hepatocytes. [file 12967_2014_230_MOESM3_ESM.docx]

| **Table S2.**  Genes Differentially Expressed Between Microdissected Malignant Hepatocytes and Non-Malignant Hepatocytes | | |
| --- | --- | --- |
| Gene Symbol | Gene Title | Fold Change |
| AKR1B10 | aldo-keto reductase family 1, member B10 (aldose reductase) | 56.2 |
| SPINK1 | serine peptidase inhibitor, Kazal type 1 | 29.7 |
| SULT1C2 | sulfotransferase family, cytosolic, 1C, member 2 | 25.0 |
| CRNDE | colorectal neoplasia differentially expressed (non-protein coding) | 24.7 |
| REG3A | regenerating islet-derived 3 alpha | 24.1 |
| SPP1 | secreted phosphoprotein 1 | 22.6 |
| GPC3 | glypican 3 | 22.0 |
| LOC344887 | NmrA-like family domain containing 1 pseudogene | 20.1 |
| ZIC2 | Zic family member 2 | 16.8 |
| ROBO1 | roundabout, axon guidance receptor, homolog 1 (Drosophila) | 15.3 |
| RBM24 | RNA binding motif protein 24 | 13.5 |
| ANLN | anillin, actin binding protein | 12.5 |
| FGF13 | fibroblast growth factor 13 | 12.4 |
| MAGEA3/MAGEA6 | melanoma antigen family A, 6 | 12.1 |
| SLC7A11 | solute carrier family 7 (anionic amino acid transporter light chain, xc- system), member 11 | 11.2 |
| NRCAM | neuronal cell adhesion molecule | 11.0 |
| TOP2A | topoisomerase (DNA) II alpha 170kDa | 10.8 |
| ACSL4 | acyl-CoA synthetase long-chain family member 4 | 10.7 |
| IGF2BP3 | insulin-like growth factor 2 mRNA binding protein 3 | 9.6 |
| CAP2 | CAP, adenylate cyclase-associated protein, 2 (yeast) | 9.4 |
| PEG10 | paternally expressed 10 | 9.2 |
| CD109 | CD109 molecule | 9.1 |
| TRIM16 | tripartite motif containing 16 | 8.6 |
| LEF1 | lymphoid enhancer-binding factor 1 | 8.6 |
| ASPM | asp (abnormal spindle) homolog, microcephaly associated (Drosophila) | 8.3 |
| HMMR | hyaluronan-mediated motility receptor (RHAMM) | 8.2 |
| DNAJC6 | DnaJ (Hsp40) homolog, subfamily C, member 6 | 8.1 |
| CDK1 | cyclin-dependent kinase 1 | 8.1 |
| GPR158 | G protein-coupled receptor 158 | 8.0 |
| ECT2 | epithelial cell transforming sequence 2 oncogene | 7.8 |
| LCN2 | lipocalin 2 | 7.7 |
| CTHRC1 | collagen triple helix repeat containing 1 | 7.7 |
| MAP2 | microtubule-associated protein 2 | 7.6 |
| CCNB1 | cyclin B1 | 7.6 |
| DYNC1I1 | dynein, cytoplasmic 1, intermediate chain 1 | 7.4 |
| SPARCL1 | SPARC-like 1 (hevin) | 7.4 |
| ZIC1 | Zic family member 1 | 7.4 |
| PRKAA2 | protein kinase, AMP-activated, alpha 2 catalytic subunit | 7.2 |
| NDC80 | NDC80 kinetochore complex component homolog (S. cerevisiae) | 7.1 |
| PRC1 | protein regulator of cytokinesis 1 | 7.1 |
| RACGAP1 | Rac GTPase activating protein 1 | 7.0 |
| NEK2 | NIMA (never in mitosis gene a)-related kinase 2 | 6.9 |
| CENPF | centromere protein F, 350/400kDa (mitosin) | 6.7 |
| TKT | transketolase | 6.6 |
| E2F7 | E2F transcription factor 7 | 6.5 |
| LINC00348 | long intergenic non-protein coding RNA 348 | 6.4 |
| FAM72D | family with sequence similarity 72, member D | 6.4 |
| BUB1B | budding uninhibited by benzimidazoles 1 homolog beta (yeast) | 6.3 |
| B3GNT5 | UDP-GlcNAc:betaGal beta-1,3-N-acetylglucosaminyltransferase 5 | 6.3 |
| FAM83D | family with sequence similarity 83, member D | 6.2 |
| RRAGD | Ras-related GTP binding D | 6.1 |
| CHRM3 | cholinergic receptor, muscarinic 3 | 6.1 |
| KIF20A | kinesin family member 20A | 6.1 |
| C12orf75 | chromosome 12 open reading frame 75 | 6.1 |
| PBK | PDZ binding kinase | 6.1 |
| MELK | maternal embryonic leucine zipper kinase | 6.0 |
| SMPX | small muscle protein, X-linked | 6.0 |
| SLC44A5 | solute carrier family 44, member 5 | 6.0 |
| EBF1 | early B-cell factor 1 | 5.9 |
| FABP5 | fatty acid binding protein 5 (psoriasis-associated) | 5.9 |
| MRAP2 | melanocortin 2 receptor accessory protein 2 | 5.9 |
| NUF2 | NUF2, NDC80 kinetochore complex component, homolog (S. cerevisiae) | 5.8 |
| GLUL | glutamate-ammonia ligase | 5.7 |
| AKR1C3 | aldo-keto reductase family 1, member C3 (3-alpha hydroxysteroid dehydrogenase, type II) | 5.7 |
| VASH2 | vasohibin 2 | 5.7 |
| BCAT1 | branched chain amino-acid transaminase 1, cytosolic | 5.6 |
| KIF14 | kinesin family member 14 | 5.5 |
| HSPB8 | heat shock 22kDa protein 8 | 5.5 |
| PI15 | peptidase inhibitor 15 | 5.5 |
| HIST1H3A | histone cluster 1, H3a | 5.5 |
| DKK3 | dickkopf 3 homolog (Xenopus laevis) | 5.5 |
| CDC20 | cell division cycle 20 homolog (S. cerevisiae) | 5.4 |
| LY96 | lymphocyte antigen 96 | 5.4 |
| COL15A1 | collagen, type XV, alpha 1 | 5.3 |
| HOXA3 | homeobox A3 | 5.2 |
| RRM2 | ribonucleotide reductase M2 | 5.1 |
| LPCAT2 | lysophosphatidylcholine acyltransferase 2 | 5.1 |
| ITGA2 | integrin, alpha 2 (CD49B, alpha 2 subunit of VLA-2 receptor) | 5.1 |
| CENPA | centromere protein A | 5.0 |
| EDIL3 | EGF-like repeats and discoidin I-like domains 3 | 5.0 |
| DLGAP5 | discs, large (Drosophila) homolog-associated protein 5 | 4.9 |
| ILDR2 | immunoglobulin-like domain containing receptor 2 | 4.9 |
| CENPW | centromere protein W | 4.9 |
| CEP55 | centrosomal protein 55kDa | 4.9 |
| TXNRD1 | thioredoxin reductase 1 | 4.9 |
| PLCB1 | phospholipase C, beta 1 (phosphoinositide-specific) | 4.8 |
| TSPAN5 | tetraspanin 5 | 4.8 |
| KIF11 | kinesin family member 11 | 4.8 |
| CTSC | cathepsin C | 4.7 |
| PRR11 | proline rich 11 | 4.7 |
| MAD2L1 | MAD2 mitotic arrest deficient-like 1 (yeast) | 4.7 |
| LMOD1 | leiomodin 1 (smooth muscle) | 4.7 |
| HOXA13 | homeobox A13 | 4.6 |
| MECOM | MDS1 and EVI1 complex locus | 4.6 |
| MYEF2 | myelin expression factor 2 | 4.6 |
| CCNB2 | cyclin B2 | 4.6 |
| SLC2A5 | solute carrier family 2 (facilitated glucose/fructose transporter), member 5 | 4.6 |
| PCDH17 | protocadherin 17 | 4.5 |
| MCTP1 | multiple C2 domains, transmembrane 1 | 4.5 |
| TTK | TTK protein kinase | 4.5 |
| CCDC34 | coiled-coil domain containing 34 | 4.4 |
| CRTAP | cartilage associated protein | 4.4 |
| CDKN2C | cyclin-dependent kinase inhibitor 2C (p18, inhibits CDK4) | 4.4 |
| EML6 | echinoderm microtubule associated protein like 6 | 4.3 |
| IRX3 | iroquois homeobox 3 | 4.3 |
| GINS1 | GINS complex subunit 1 (Psf1 homolog) | 4.3 |
| KIF4A | kinesin family member 4A | 4.3 |
| SRXN1 | sulfiredoxin 1 | 4.3 |
| FLJ39632 | uncharacterized LOC642477 | 4.3 |
| TRIP13 | thyroid hormone receptor interactor 13 | 4.2 |
| LPL | lipoprotein lipase | 4.2 |
| SGOL2 | shugoshin-like 2 (S. pombe) | 4.2 |
| PODXL | podocalyxin-like | 4.2 |
| PIR | pirin (iron-binding nuclear protein) | 4.2 |
| EFCAB2 | EF-hand calcium binding domain 2 | 4.2 |
| GOLM1 | golgi membrane protein 1 | 4.1 |
| UGT1A6 | UDP glucuronosyltransferase 1 family, polypeptide A6 | 4.1 |
| BIRC5 | baculoviral IAP repeat containing 5 | 4.1 |
| ITGA6 | integrin, alpha 6 | 4.1 |
| LOC100132891 | uncharacterized LOC100132891 | 4.1 |
| NUSAP1 | nucleolar and spindle associated protein 1 | 4.0 |
| APOBEC3B | apolipoprotein B mRNA editing enzyme, catalytic polypeptide-like 3B | 3.9 |
| SMYD3 | SET and MYND domain containing 3 | 3.9 |
| SACS | spastic ataxia of Charlevoix-Saguenay (sacsin) | 3.9 |
| SLC38A6 | solute carrier family 38, member 6 | 3.9 |
| GBAP1 | glucosidase, beta, acid pseudogene 1 | 3.9 |
| HS2ST1 | heparan sulfate 2-O-sulfotransferase 1 | 3.9 |
| DNAH12 | dynein, axonemal, heavy chain 12 | 3.9 |
| VNN2 | vanin 2 | 3.8 |
| SLC26A2 | solute carrier family 26 (sulfate transporter), member 2 | 3.8 |
| STXBP6 | syntaxin binding protein 6 (amisyn) | 3.8 |
| HSPB1 | heat shock 27kDa protein 1 | 3.8 |
| TMTC1 | transmembrane and tetratricopeptide repeat containing 1 | 3.8 |
| FRMD3 | FERM domain containing 3 | 3.8 |
| CEP41 | centrosomal protein 41kDa | 3.8 |
| FOXM1 | forkhead box M1 | 3.8 |
| SMOC2 | SPARC related modular calcium binding 2 | 3.8 |
| TP53I3 | tumor protein p53 inducible protein 3 | 3.8 |
| ACSL6 | acyl-CoA synthetase long-chain family member 6 | 3.7 |
| DEPDC1B | DEP domain containing 1B | 3.7 |
| AKR1C1/AKR1C2 | aldo-keto reductase family 1, member C2 (dihydrodiol dehydrogenase 2; bile acid binding protein; 3-alpha hydroxysteroid dehydrogenase, type III) | 3.7 |
| RAD51AP1 | RAD51 associated protein 1 | 3.7 |
| FAM169A | family with sequence similarity 169, member A | 3.7 |
| CDKN2B | cyclin-dependent kinase inhibitor 2B (p15, inhibits CDK4) | 3.7 |
| LGALS8 | lectin, galactoside-binding, soluble, 8 | 3.7 |
| CENPK | centromere protein K | 3.7 |
| ENAH | enabled homolog (Drosophila) | 3.6 |
| BAG2 | BCL2-associated athanogene 2 | 3.6 |
| CNKSR2 | connector enhancer of kinase suppressor of Ras 2 | 3.6 |
| C1orf85 | chromosome 1 open reading frame 85 | 3.6 |
| GCNT3 | glucosaminyl (N-acetyl) transferase 3, mucin type | 3.6 |
| KIAA1199 | KIAA1199 | 3.6 |
| AURKA | aurora kinase A | 3.6 |
| STAU2 | staufen, RNA binding protein, homolog 2 (Drosophila) | 3.6 |
| CCNE2 | cyclin E2 | 3.6 |
| PTTG1 | pituitary tumor-transforming 1 | 3.6 |
| CDC7 | cell division cycle 7 homolog (S. cerevisiae) | 3.6 |
| EID3 | EP300 interacting inhibitor of differentiation 3 | 3.6 |
| ASF1A | ASF1 anti-silencing function 1 homolog A (S. cerevisiae) | 3.5 |
| FIGNL1 | fidgetin-like 1 | 3.5 |
| MSH2 | mutS homolog 2, colon cancer, nonpolyposis type 1 (E. coli) | 3.5 |
| MCAM | melanoma cell adhesion molecule | 3.5 |
| E2F8 | E2F transcription factor 8 | 3.5 |
| NCAPG | non-SMC condensin I complex, subunit G | 3.5 |
| ANGPT1 | angiopoietin 1 | 3.5 |
| DLG5 | discs, large homolog 5 (Drosophila) | 3.5 |
| FANCD2 | Fanconi anemia, complementation group D2 | 3.5 |
| ZWINT | ZW10 interactor | 3.5 |
| SERPINI1 | serpin peptidase inhibitor, clade I (neuroserpin), member 1 | 3.5 |
| LAMC1 | laminin, gamma 1 (formerly LAMB2) | 3.5 |
| ZSWIM5 | zinc finger, SWIM-type containing 5 | 3.5 |
| CCNA2 | cyclin A2 | 3.4 |
| HSP90AB1 | heat shock protein 90kDa alpha (cytosolic), class B member 1 | 3.4 |
| ASAP2 | ArfGAP with SH3 domain, ankyrin repeat and PH domain 2 | 3.4 |
| MARCKS | myristoylated alanine-rich protein kinase C substrate | 3.4 |
| CD200 | CD200 molecule | 3.4 |
| CLIC5 | chloride intracellular channel 5 | 3.4 |
| LOC389834 | ankyrin repeat domain 57 pseudogene | 3.4 |
| C6orf62 | chromosome 6 open reading frame 62 | 3.4 |
| STK39 | serine threonine kinase 39 | 3.4 |
| ANKRD29 | ankyrin repeat domain 29 | 3.4 |
| CKAP2 | cytoskeleton associated protein 2 | 3.4 |
| GNAI1 | guanine nucleotide binding protein (G protein), alpha inhibiting activity polypeptide 1 | 3.4 |
| KITLG | KIT ligand | 3.4 |
| SWAP70 | SWAP switching B-cell complex 70kDa subunit | 3.4 |
| LOC100288911 | uncharacterized LOC100288911 | 3.4 |
| TBCE | tubulin folding cofactor E | 3.3 |
| LOC541471 | uncharacterized LOC541471 | 3.3 |
| ANXA2 | annexin A2 | 3.3 |
| SPON2 | spondin 2, extracellular matrix protein | 3.3 |
| TMEM64 | transmembrane protein 64 | 3.3 |
| FLVCR1 | feline leukemia virus subgroup C cellular receptor 1 | 3.3 |
| MLF1IP | MLF1 interacting protein | 3.3 |
| MPPED2 | metallophosphoesterase domain containing 2 | 3.3 |
| GJC1 | gap junction protein, gamma 1, 45kDa | 3.3 |
| SCRN1 | secernin 1 | 3.3 |
| RBP7 | retinol binding protein 7, cellular | 3.2 |
| MPP7 | membrane protein, palmitoylated 7 (MAGUK p55 subfamily member 7) | 3.2 |
| B3GALNT1 | beta-1,3-N-acetylgalactosaminyltransferase 1 (globoside blood group) | 3.2 |
| LINC00094 | long intergenic non-protein coding RNA 94 | 3.2 |
| ATP1B3 | ATPase, Na+/K+ transporting, beta 3 polypeptide | 3.2 |
| EIF5A2 | eukaryotic translation initiation factor 5A2 | 3.2 |
| ZNF738 | zinc finger protein 738 | 3.2 |
| TPX2 | TPX2, microtubule-associated, homolog (Xenopus laevis) | 3.2 |
| CDKN2A | cyclin-dependent kinase inhibitor 2A | 3.2 |
| P4HA2 | prolyl 4-hydroxylase, alpha polypeptide II | 3.2 |
| S100A10 | S100 calcium binding protein A10 | 3.2 |
| CDKN3 | cyclin-dependent kinase inhibitor 3 | 3.2 |
| ANKRD27 | ankyrin repeat domain 27 (VPS9 domain) | 3.2 |
| HTATIP2 | HIV-1 Tat interactive protein 2, 30kDa | 3.1 |
| KPNA2 | karyopherin alpha 2 (RAG cohort 1, importin alpha 1) | 3.1 |
| EPDR1 | ependymin related protein 1 (zebrafish) | 3.1 |
| STMN1 | stathmin 1 | 3.1 |
| ADAM9 | ADAM metallopeptidase domain 9 | 3.1 |
| GNAL | guanine nucleotide binding protein (G protein), alpha activating activity polypeptide, olfactory type | 3.1 |
| SLC35G2 | solute carrier family 35, member G2 | 3.1 |
| TUBG1 | tubulin, gamma 1 | 3.1 |
| ASPH | aspartate beta-hydroxylase | 3.1 |
| VWF | von Willebrand factor | 3.1 |
| STIL | SCL/TAL1 interrupting locus | 3.1 |
| SCPEP1 | serine carboxypeptidase 1 | 3.1 |
| LMNA | lamin A/C | 3.1 |
| MRPS23 | mitochondrial ribosomal protein S23 | 3.1 |
| KIAA1462 | KIAA1462 | 3.1 |
| ZC2HC1A | zinc finger, C2HC-type containing 1A | 3.1 |
| DTNA | dystrobrevin, alpha | 3.1 |
| MYO5A | myosin VA (heavy chain 12, myoxin) | 3.1 |
| SPATS2 | spermatogenesis associated, serine-rich 2 | 3.1 |
| IGSF3 | immunoglobulin superfamily, member 3 | 3.0 |
| CNIH4 | cornichon homolog 4 (Drosophila) | 3.0 |
| AIM1L | absent in melanoma 1-like | 3.0 |
| THSD7A | thrombospondin, type I, domain containing 7A | 3.0 |
| CDCA3 | cell division cycle associated 3 | 3.0 |
| KIAA0101 | KIAA0101 | 3.0 |
| NEU1 | sialidase 1 (lysosomal sialidase) | 3.0 |
| OSBPL3 | oxysterol binding protein-like 3 | 3.0 |
| LOC401068 | uncharacterized LOC401068 | 3.0 |
| SLC39A10 | solute carrier family 39 (zinc transporter), member 10 | 3.0 |
| GJA1 | gap junction protein, alpha 1, 43kDa | 3.0 |
| RCN2 | reticulocalbin 2, EF-hand calcium binding domain | 3.0 |
| NPL | N-acetylneuraminate pyruvate lyase (dihydrodipicolinate synthase) | 3.0 |
| CLIC1 | chloride intracellular channel 1 | 3.0 |
| ATP6V1C1 | ATPase, H+ transporting, lysosomal 42kDa, V1 subunit C1 | 3.0 |
| ARHGAP44 | Rho GTPase activating protein 44 | 3.0 |
| S100A11 | S100 calcium binding protein A11 | 3.0 |
| DTL | denticleless E3 ubiquitin protein ligase homolog (Drosophila) | 3.0 |
| NEB | nebulin | 3.0 |
| TMEM164 | transmembrane protein 164 | 3.0 |
| TMEM106C | transmembrane protein 106C | 2.9 |
| ZNF323 | zinc finger protein 323 | 2.9 |
| TRIM59 | tripartite motif containing 59 | 2.9 |
| TOMM40L | translocase of outer mitochondrial membrane 40 homolog (yeast)-like | 2.9 |
| PTPN14 | protein tyrosine phosphatase, non-receptor type 14 | 2.9 |
| DCK | deoxycytidine kinase | 2.9 |
| AIDA | axin interactor, dorsalization associated | 2.9 |
| GNS | glucosamine (N-acetyl)-6-sulfatase | 2.9 |
| TAF1A | TATA box binding protein (TBP)-associated factor, RNA polymerase I, A, 48kDa | 2.9 |
| FBXO32 | F-box protein 32 | 2.9 |
| MANEAL | mannosidase, endo-alpha-like | 2.9 |
| STEAP2 | STEAP family member 2, metalloreductase | 2.9 |
| CTSA | cathepsin A | 2.9 |
| COL4A1 | collagen, type IV, alpha 1 | 2.9 |
| IQGAP3 | IQ motif containing GTPase activating protein 3 | 2.9 |
| TRPS1 | trichorhinophalangeal syndrome I | 2.9 |
| RNF157 | ring finger protein 157 | 2.9 |
| TRAF5 | TNF receptor-associated factor 5 | 2.9 |
| SLC7A6 | solute carrier family 7 (amino acid transporter light chain, y+L system), member 6 | 2.9 |
| PALLD | palladin, cytoskeletal associated protein | 2.9 |
| ITGAM | integrin, alpha M (complement component 3 receptor 3 subunit) | 2.8 |
| LOC100507316 | uncharacterized LOC100507316 | 2.8 |
| MTHFD1L | methylenetetrahydrofolate dehydrogenase (NADP+ dependent) 1-like | 2.8 |
| SPAG4 | sperm associated antigen 4 | 2.8 |
| FSD1L | fibronectin type III and SPRY domain containing 1-like | 2.8 |
| RFC4 | replication factor C (activator 1) 4, 37kDa | 2.8 |
| TUBB2A | tubulin, beta 2A class IIa | 2.8 |
| CCDC99 | coiled-coil domain containing 99 | 2.8 |
| PEA15 | phosphoprotein enriched in astrocytes 15 | 2.8 |
| CD58 | CD58 molecule | 2.8 |
| ZNF232 | zinc finger protein 232 | 2.8 |
| ALDOA | aldolase A, fructose-bisphosphate | 2.8 |
| BAMBI | BMP and activin membrane-bound inhibitor homolog (Xenopus laevis) | 2.8 |
| ILF2 | interleukin enhancer binding factor 2, 45kDa | 2.8 |
| FAM54A | family with sequence similarity 54, member A | 2.8 |
| SEMA3G | sema domain, immunoglobulin domain (Ig), short basic domain, secreted, (semaphorin) 3G | 2.8 |
| PFKFB2 | 6-phosphofructo-2-kinase/fructose-2,6-biphosphatase 2 | 2.8 |
| PPP1R2 | protein phosphatase 1, regulatory (inhibitor) subunit 2 | 2.8 |
| C11orf93 | chromosome 11 open reading frame 93 | 2.8 |
| FRZB | frizzled-related protein | 2.8 |
| GGPS1 | geranylgeranyl diphosphate synthase 1 | 2.8 |
| SYT1 | synaptotagmin I | 2.8 |
| PRIM1 | primase, DNA, polypeptide 1 (49kDa) | 2.8 |
| C1orf43 | chromosome 1 open reading frame 43 | 2.8 |
| PTGFRN | prostaglandin F2 receptor negative regulator | 2.8 |
| ZEB1-AS1 | ZEB1 antisense RNA 1 | 2.8 |
| SMC2 | structural maintenance of chromosomes 2 | 2.8 |
| COL4A2 | collagen, type IV, alpha 2 | 2.8 |
| FAT1 | FAT tumor suppressor homolog 1 (Drosophila) | 2.7 |
| SKAP2 | src kinase associated phosphoprotein 2 | 2.7 |
| RDBP | RD RNA binding protein | 2.7 |
| TPM2 | tropomyosin 2 (beta) | 2.7 |
| ACTN2 | actinin, alpha 2 | 2.7 |
| MATR3 | matrin 3 | 2.7 |
| CACYBP | calcyclin binding protein | 2.7 |
| GGH | gamma-glutamyl hydrolase (conjugase, folylpolygammaglutamyl hydrolase) | 2.7 |
| LINC00622 | long intergenic non-protein coding RNA 622 | 2.7 |
| CDK5 | cyclin-dependent kinase 5 | 2.7 |
| SOCS5 | suppressor of cytokine signaling 5 | 2.7 |
| FERMT1 | fermitin family member 1 | 2.7 |
| SGCE | sarcoglycan, epsilon | 2.7 |
| NLRP1 | NLR family, pyrin domain containing 1 | 2.7 |
| FAM198B | family with sequence similarity 198, member B | 2.7 |
| PRCC | papillary renal cell carcinoma (translocation-associated) | 2.7 |
| SQSTM1 | sequestosome 1 | 2.7 |
| LOX | lysyl oxidase | 2.6 |
| BTG3 | BTG family, member 3 | 2.6 |
| MFSD6 | major facilitator superfamily domain containing 6 | 2.6 |
| AGPAT4 | 1-acylglycerol-3-phosphate O-acyltransferase 4 (lysophosphatidic acid acyltransferase, delta) | 2.6 |
| ZBTB41 | zinc finger and BTB domain containing 41 | 2.6 |
| ARPC5 | actin related protein 2/3 complex, subunit 5, 16kDa | 2.6 |
| TNFSF4 | tumor necrosis factor (ligand) superfamily, member 4 | 2.6 |
| ASRGL1 | asparaginase like 1 | 2.6 |
| TUBA1B | tubulin, alpha 1b | 2.6 |
| HN1 | hematological and neurological expressed 1 | 2.6 |
| TMEM98 | transmembrane protein 98 | 2.6 |
| NCAPD2 | non-SMC condensin I complex, subunit D2 | 2.6 |
| WDR67 | WD repeat domain 67 | 2.6 |
| EPS8L3 | EPS8-like 3 | 2.6 |
| EZH2 | enhancer of zeste homolog 2 (Drosophila) | 2.6 |
| ENO1 | enolase 1, (alpha) | 2.6 |
| TMEM48 | transmembrane protein 48 | 2.6 |
| MPZL1 | myelin protein zero-like 1 | 2.6 |
| DUT | deoxyuridine triphosphatase | 2.6 |
| PANX2 | pannexin 2 | 2.6 |
| IMPAD1 | inositol monophosphatase domain containing 1 | 2.6 |
| LRP12 | low density lipoprotein receptor-related protein 12 | 2.6 |
| PHB | prohibitin | 2.6 |
| ZNF107 | zinc finger protein 107 | 2.6 |
| MEOX2 | mesenchyme homeobox 2 | 2.6 |
| RAP2A | RAP2A, member of RAS oncogene family | 2.6 |
| AIF1L | allograft inflammatory factor 1-like | 2.6 |
| DNM3 | dynamin 3 | 2.6 |
| IER5 | immediate early response 5 | 2.6 |
| PRKDC | protein kinase, DNA-activated, catalytic polypeptide | 2.6 |
| SUCO | SUN domain containing ossification factor | 2.6 |
| FEN1 | flap structure-specific endonuclease 1 | 2.6 |
| RIT1 | Ras-like without CAAX 1 | 2.6 |
| RAB3B | RAB3B, member RAS oncogene family | 2.6 |
| MTMR11 | myotubularin related protein 11 | 2.6 |
| ANXA2P2 | annexin A2 pseudogene 2 | 2.6 |
| NME1 | NME/NM23 nucleoside diphosphate kinase 1 | 2.5 |
| MIF | macrophage migration inhibitory factor (glycosylation-inhibiting factor) | 2.5 |
| FAM129A | family with sequence similarity 129, member A | 2.5 |
| KIF3A | kinesin family member 3A | 2.5 |
| CCT3 | chaperonin containing TCP1, subunit 3 (gamma) | 2.5 |
| NETO2 | neuropilin (NRP) and tolloid (TLL)-like 2 | 2.5 |
| SPATA5 | spermatogenesis associated 5 | 2.5 |
| PTPRG | protein tyrosine phosphatase, receptor type, G | 2.5 |
| TCEB1 | transcription elongation factor B (SIII), polypeptide 1 (15kDa, elongin C) | 2.5 |
| B4GALT6 | UDP-Gal:betaGlcNAc beta 1,4- galactosyltransferase, polypeptide 6 | 2.5 |
| RRP15 | ribosomal RNA processing 15 homolog (S. cerevisiae) | 2.5 |
| PIEZO2 | piezo-type mechanosensitive ion channel component 2 | 2.5 |
| HMGB2 | high mobility group box 2 | 2.5 |
| C7orf23 | chromosome 7 open reading frame 23 | 2.5 |
| RFX5 | regulatory factor X, 5 (influences HLA class II expression) | 2.5 |
| ABCC1 | ATP-binding cassette, sub-family C (CFTR/MRP), member 1 | 2.5 |
| DDOST | dolichyl-diphosphooligosaccharide--protein glycosyltransferase | 2.5 |
| STC1 | stanniocalcin 1 | 2.5 |
| H2AFX | H2A histone family, member X | 2.5 |
| RGS5 | regulator of G-protein signaling 5 | 2.5 |
| UBE2C | ubiquitin-conjugating enzyme E2C | 2.5 |
| SNN | stannin | 2.5 |
| CKLF | chemokine-like factor | 2.5 |
| TTLL7 | tubulin tyrosine ligase-like family, member 7 | 2.5 |
| GALNT10 | UDP-N-acetyl-alpha-D-galactosamine:polypeptide N-acetylgalactosaminyltransferase 10 (GalNAc-T10) | 2.5 |
| DPCD | deleted in primary ciliary dyskinesia homolog (mouse) | 2.5 |
| RNF43 | ring finger protein 43 | 2.5 |
| SLC1A3 | solute carrier family 1 (glial high affinity glutamate transporter), member 3 | 2.5 |
| ASAP1 | ArfGAP with SH3 domain, ankyrin repeat and PH domain 1 | 2.5 |
| HIST1H2AG | histone cluster 1, H2ag | 2.5 |
| CSTB | cystatin B (stefin B) | 2.5 |
| CAMSAP2 | calmodulin regulated spectrin-associated protein family, member 2 | 2.5 |
| TUBA1C | tubulin, alpha 1c | 2.5 |
| PPT1 | palmitoyl-protein thioesterase 1 | 2.5 |
| FKBP11 | FK506 binding protein 11, 19 kDa | 2.5 |
| LOC284513 | uncharacterized LOC284513 | 2.5 |
| SORT1 | sortilin 1 | 2.4 |
| TAGLN2 | transgelin 2 | 2.4 |
| VWA5A | von Willebrand factor A domain containing 5A | 2.4 |
| TMOD2 | tropomodulin 2 (neuronal) | 2.4 |
| SMC4 | structural maintenance of chromosomes 4 | 2.4 |
| FAM217B | family with sequence similarity 217, member B | 2.4 |
| MCM7 | minichromosome maintenance complex component 7 | 2.4 |
| SPTBN1 | spectrin, beta, non-erythrocytic 1 | 2.4 |
| ELK3 | ELK3, ETS-domain protein (SRF accessory protein 2) | 2.4 |
| PSPH | phosphoserine phosphatase | 2.4 |
| HIST1H2AC | histone cluster 1, H2ac | 2.4 |
| HOXD8 | homeobox D8 | 2.4 |
| CEP68 | centrosomal protein 68kDa | 2.4 |
| PECAM1 | platelet/endothelial cell adhesion molecule 1 | 2.4 |
| TRIM45 | tripartite motif containing 45 | 2.4 |
| PIGF | phosphatidylinositol glycan anchor biosynthesis, class F | 2.4 |
| TACC1 | transforming, acidic coiled-coil containing protein 1 | 2.4 |
| BOP1 | block of proliferation 1 | 2.4 |
| IFT81 | intraflagellar transport 81 homolog (Chlamydomonas) | 2.4 |
| MYBL1 | v-myb myeloblastosis viral oncogene homolog (avian)-like 1 | 2.4 |
| PRTFDC1 | phosphoribosyl transferase domain containing 1 | 2.4 |
| FAM13C | family with sequence similarity 13, member C | 2.4 |
| C1orf198 | chromosome 1 open reading frame 198 | 2.4 |
| TEAD2 | TEA domain family member 2 | 2.4 |
| ZKSCAN3 | zinc finger with KRAB and SCAN domains 3 | 2.4 |
| SERF1A/SERF1B | small EDRK-rich factor 1A (telomeric) | 2.4 |
| UTP23 | UTP23, small subunit (SSU) processome component, homolog (yeast) | 2.4 |
| CD93 | CD93 molecule | 2.4 |
| RAN | RAN, member RAS oncogene family | 2.4 |
| LPGAT1 | lysophosphatidylglycerol acyltransferase 1 | 2.4 |
| PCSK5 | proprotein convertase subtilisin/kexin type 5 | 2.4 |
| ANP32E | acidic (leucine-rich) nuclear phosphoprotein 32 family, member E | 2.4 |
| TRIM6 | tripartite motif containing 6 | 2.4 |
| CANX | calnexin | 2.4 |
| GAPDH | glyceraldehyde-3-phosphate dehydrogenase | 2.4 |
| AFAP1L1 | actin filament associated protein 1-like 1 | 2.4 |
| CCDC77 | coiled-coil domain containing 77 | 2.4 |
| DISC1 | disrupted in schizophrenia 1 | 2.4 |
| SAP30 | Sin3A-associated protein, 30kDa | 2.3 |
| TMEM144 | transmembrane protein 144 | 2.3 |
| ZBTB26 | zinc finger and BTB domain containing 26 | 2.3 |
| CFL1 | cofilin 1 (non-muscle) | 2.3 |
| PLCE1 | phospholipase C, epsilon 1 | 2.3 |
| PDK1 | pyruvate dehydrogenase kinase, isozyme 1 | 2.3 |
| PPP1R16A | protein phosphatase 1, regulatory subunit 16A | 2.3 |
| C3orf32 | chromosome 3 open reading frame 32 | 2.3 |
| RASAL2 | RAS protein activator like 2 | 2.3 |
| KCNE3 | potassium voltage-gated channel, Isk-related family, member 3 | 2.3 |
| ADA | adenosine deaminase | 2.3 |
| IGDCC4 | immunoglobulin superfamily, DCC subclass, member 4 | 2.3 |
| RHOA | ras homolog family member A | 2.3 |
| C2orf76 | chromosome 2 open reading frame 76 | 2.3 |
| SLC25A43 | solute carrier family 25, member 43 | 2.3 |
| TXN | thioredoxin | 2.3 |
| SAC3D1 | SAC3 domain containing 1 | 2.3 |
| LPCAT1 | lysophosphatidylcholine acyltransferase 1 | 2.3 |
| SH3RF2 | SH3 domain containing ring finger 2 | 2.3 |
| DYNLL1 | dynein, light chain, LC8-type 1 | 2.3 |
| CBX4 | chromobox homolog 4 | 2.3 |
| IPO11 | importin 11 | 2.3 |
| DR1 | down-regulator of transcription 1, TBP-binding (negative cofactor 2) | 2.3 |
| MLEC | malectin | 2.3 |
| ZNF703 | zinc finger protein 703 | 2.3 |
| BBS12 | Bardet-Biedl syndrome 12 | 2.3 |
| TBC1D16 | TBC1 domain family, member 16 | 2.3 |
| ARF1 | ADP-ribosylation factor 1 | 2.3 |
| UGGT1 | UDP-glucose glycoprotein glucosyltransferase 1 | 2.3 |
| YWHAB | tyrosine 3-monooxygenase/tryptophan 5-monooxygenase activation protein, beta polypeptide | 2.3 |
| BROX | BRO1 domain and CAAX motif containing | 2.3 |
| GOLPH3L | golgi phosphoprotein 3-like | 2.2 |
| SRGAP2 | SLIT-ROBO Rho GTPase activating protein 2 | 2.2 |
| GLA | galactosidase, alpha | 2.2 |
| CPD | carboxypeptidase D | 2.2 |
| LOC100505519 | uncharacterized LOC100505519 | 2.2 |
| PRLR | prolactin receptor | 2.2 |
| METTL18 | methyltransferase like 18 | 2.2 |
| C11orf73 | chromosome 11 open reading frame 73 | 2.2 |
| MTMR2 | myotubularin related protein 2 | 2.2 |
| TIPIN | TIMELESS interacting protein | 2.2 |
| OVOS/OVOS2 | ovostatin | 2.2 |
| ADAMTS9 | ADAM metallopeptidase with thrombospondin type 1 motif, 9 | 2.2 |
| MRPS12 | mitochondrial ribosomal protein S12 | 2.2 |
| PTBP3 | polypyrimidine tract binding protein 3 | 2.2 |
| PSME3 | proteasome (prosome, macropain) activator subunit 3 (PA28 gamma; Ki) | 2.2 |
| CCDC90A | coiled-coil domain containing 90A | 2.2 |
| SEPT8 | septin 8 | 2.2 |
| AP3M2 | adaptor-related protein complex 3, mu 2 subunit | 2.2 |
| SLC52A2 | solute carrier family 52, riboflavin transporter, member 2 | 2.2 |
| STRA13 | stimulated by retinoic acid 13 homolog (mouse) | 2.2 |
| COMMD8 | COMM domain containing 8 | 2.2 |
| CLN3 | ceroid-lipofuscinosis, neuronal 3 | 2.2 |
| USP46 | ubiquitin specific peptidase 46 | 2.2 |
| DLAT | dihydrolipoamide S-acetyltransferase | 2.2 |
| ABCC5 | ATP-binding cassette, sub-family C (CFTR/MRP), member 5 | 2.2 |
| SPA17 | sperm autoantigenic protein 17 | 2.2 |
| C8orf59 | chromosome 8 open reading frame 59 | 2.2 |
| ACLY | ATP citrate lyase | 2.2 |
| NAP1L1 | nucleosome assembly protein 1-like 1 | 2.2 |
| TSEN15 | tRNA splicing endonuclease 15 homolog (S. cerevisiae) | 2.2 |
| TOMM20 | translocase of outer mitochondrial membrane 20 homolog (yeast) | 2.2 |
| DSTYK | dual serine/threonine and tyrosine protein kinase | 2.2 |
| TGFB2 | transforming growth factor, beta 2 | 2.2 |
| MAP4K4 | mitogen-activated protein kinase kinase kinase kinase 4 | 2.2 |
| POLR3K | polymerase (RNA) III (DNA directed) polypeptide K, 12.3 kDa | 2.2 |
| LOC642852 | uncharacterized LOC642852 | 2.2 |
| SMAD2 | SMAD family member 2 | 2.2 |
| UXS1 | UDP-glucuronate decarboxylase 1 | 2.2 |
| UNKL | unkempt homolog (Drosophila)-like | 2.2 |
| KBTBD2 | kelch repeat and BTB (POZ) domain containing 2 | 2.2 |
| TIGD1 | tigger transposable element derived 1 | 2.2 |
| KDM5B | lysine (K)-specific demethylase 5B | 2.2 |
| CASK | calcium/calmodulin-dependent serine protein kinase (MAGUK family) | 2.2 |
| CCDC113 | coiled-coil domain containing 113 | 2.2 |
| RHOC | ras homolog family member C | 2.2 |
| CPPED1 | calcineurin-like phosphoesterase domain containing 1 | 2.2 |
| ARPC1B | actin related protein 2/3 complex, subunit 1B, 41kDa | 2.2 |
| NOV | nephroblastoma overexpressed | 2.2 |
| LAMB3 | laminin, beta 3 | 2.2 |
| LYPLAL1 | lysophospholipase-like 1 | 2.2 |
| ATP8B2 | ATPase, aminophospholipid transporter, class I, type 8B, member 2 | 2.2 |
| AACS | acetoacetyl-CoA synthetase | 2.1 |
| FLT1 | fms-related tyrosine kinase 1 (vascular endothelial growth factor/vascular permeability factor receptor) | 2.1 |
| AGBL3 | ATP/GTP binding protein-like 3 | 2.1 |
| GPR176 | G protein-coupled receptor 176 | 2.1 |
| WASF1 | WAS protein family, member 1 | 2.1 |
| HNRNPU | heterogeneous nuclear ribonucleoprotein U (scaffold attachment factor A) | 2.1 |
| AIFM2 | apoptosis-inducing factor, mitochondrion-associated, 2 | 2.1 |
| SMARCC1 | SWI/SNF related, matrix associated, actin dependent regulator of chromatin, subfamily c, member 1 | 2.1 |
| ARHGAP18 | Rho GTPase activating protein 18 | 2.1 |
| LOC100127983 | uncharacterized LOC100127983 | 2.1 |
| ITGA7 | integrin, alpha 7 | 2.1 |
| BBS7 | Bardet-Biedl syndrome 7 | 2.1 |
| ZNF174 | zinc finger protein 174 | 2.1 |
| HIST2H2BE | histone cluster 2, H2be | 2.1 |
| ARPC1A | actin related protein 2/3 complex, subunit 1A, 41kDa | 2.1 |
| ZNF238 | zinc finger protein 238 | 2.1 |
| VARS | valyl-tRNA synthetase | 2.1 |
| DIEXF | digestive organ expansion factor homolog (zebrafish) | 2.1 |
| HEY1 | hairy/enhancer-of-split related with YRPW motif 1 | 2.1 |
| GPD2 | glycerol-3-phosphate dehydrogenase 2 (mitochondrial) | 2.1 |
| GM2A | GM2 ganglioside activator | 2.1 |
| GPATCH4 | G patch domain containing 4 | 2.1 |
| TMA16 | translation machinery associated 16 homolog (S. cerevisiae) | 2.1 |
| SFR1 | SWI5-dependent recombination repair 1 | 2.1 |
| RALA | v-ral simian leukemia viral oncogene homolog A (ras related) | 2.1 |
| GRN | granulin | 2.1 |
| IMPDH2 | IMP (inosine 5'-monophosphate) dehydrogenase 2 | 2.1 |
| GOLT1B | golgi transport 1B | 2.1 |
| S100A6 | S100 calcium binding protein A6 | 2.1 |
| ZNF704 | zinc finger protein 704 | 2.1 |
| LIX1L | Lix1 homolog (mouse)-like | 2.1 |
| HMGN4 | high mobility group nucleosomal binding domain 4 | 2.1 |
| PAPSS1 | 3'-phosphoadenosine 5'-phosphosulfate synthase 1 | 2.1 |
| C7orf60 | chromosome 7 open reading frame 60 | 2.1 |
| ATAD2 | ATPase family, AAA domain containing 2 | 2.1 |
| YEATS4 | YEATS domain containing 4 | 2.1 |
| PDIA6 | protein disulfide isomerase family A, member 6 | 2.1 |
| HEXA | hexosaminidase A (alpha polypeptide) | 2.1 |
| IRF6 | interferon regulatory factor 6 | 2.1 |
| C5orf54 | chromosome 5 open reading frame 54 | 2.1 |
| PSMB4 | proteasome (prosome, macropain) subunit, beta type, 4 | 2.1 |
| LOC100996419 | uncharacterized LOC100996419 | 2.1 |
| ZNF521 | zinc finger protein 521 | 2.1 |
| NDE1 | nudE nuclear distribution E homolog 1 (A. nidulans) | 2.1 |
| DCLK1 | doublecortin-like kinase 1 | 2.1 |
| BCAP31 | B-cell receptor-associated protein 31 | 2.1 |
| DNAJC10 | DnaJ (Hsp40) homolog, subfamily C, member 10 | 2.1 |
| MRAS | muscle RAS oncogene homolog | 2.1 |
| SSR2 | signal sequence receptor, beta (translocon-associated protein beta) | 2.1 |
| CDC42SE2 | CDC42 small effector 2 | 2.1 |
| PIK3R3 | phosphoinositide-3-kinase, regulatory subunit 3 (gamma) | 2.1 |
| MAD2L2 | MAD2 mitotic arrest deficient-like 2 (yeast) | 2.1 |
| CYTH3 | cytohesin 3 | 2.1 |
| RAB7L1 | RAB7, member RAS oncogene family-like 1 | 2.1 |
| PRUNE | prune homolog (Drosophila) | 2.1 |
| MIB1 | mindbomb E3 ubiquitin protein ligase 1 | 2.1 |
| PDZD2 | PDZ domain containing 2 | 2.1 |
| GPX1 | glutathione peroxidase 1 | 2.1 |
| RBM15 | RNA binding motif protein 15 | 2.1 |
| FADS1 | fatty acid desaturase 1 | 2.1 |
| LSM4 | LSM4 homolog, U6 small nuclear RNA associated (S. cerevisiae) | 2.1 |
| MITD1 | MIT, microtubule interacting and transport, domain containing 1 | 2.1 |
| RFC3 | replication factor C (activator 1) 3, 38kDa | 2.1 |
| DUS4L | dihydrouridine synthase 4-like (S. cerevisiae) | 2.1 |
| TAF10 | TAF10 RNA polymerase II, TATA box binding protein (TBP)-associated factor, 30kDa | 2.1 |
| DTNBP1 | dystrobrevin binding protein 1 | 2.0 |
| TULP3 | tubby like protein 3 | 2.0 |
| MSH5 | mutS homolog 5 (E. coli) | 2.0 |
| TET1 | tet methylcytosine dioxygenase 1 | 2.0 |
| CKAP4 | cytoskeleton-associated protein 4 | 2.0 |
| PRDX1 | peroxiredoxin 1 | 2.0 |
| C9orf16 | chromosome 9 open reading frame 16 | 2.0 |
| PLK1S1 | polo-like kinase 1 substrate 1 | 2.0 |
| C7orf29 | chromosome 7 open reading frame 29 | 2.0 |
| BBX | bobby sox homolog (Drosophila) | 2.0 |
| EFCAB7 | EF-hand calcium binding domain 7 | 2.0 |
| TUBB3 | tubulin, beta 3 class III | 2.0 |
| AZIN1 | antizyme inhibitor 1 | 2.0 |
| OTUD6B | OTU domain containing 6B | 2.0 |
| MPV17 | MpV17 mitochondrial inner membrane protein | 2.0 |
| HSPA13 | heat shock protein 70kDa family, member 13 | 2.0 |
| RAD21 | RAD21 homolog (S. pombe) | 2.0 |
| ZNF280C | zinc finger protein 280C | 2.0 |
| ORMDL2 | ORM1-like 2 (S. cerevisiae) | 2.0 |
| GTF2H2 | general transcription factor IIH, polypeptide 2, 44kDa | 2.0 |
| BAIAP2-AS1 | BAIAP2 antisense RNA 1 | 2.0 |
| GCKR | glucokinase (hexokinase 4) regulator | -2.0 |
| UBE2D3 | ubiquitin-conjugating enzyme E2D 3 | -2.0 |
| SPRYD4 | SPRY domain containing 4 | -2.0 |
| TXNL1 | thioredoxin-like 1 | -2.0 |
| RAB6A | RAB6A, member RAS oncogene family | -2.0 |
| JMJD1C | jumonji domain containing 1C | -2.0 |
| FGA | fibrinogen alpha chain | -2.0 |
| FLNB | filamin B, beta | -2.0 |
| SLC16A1 | solute carrier family 16, member 1 (monocarboxylic acid transporter 1) | -2.0 |
| ZNF542 | zinc finger protein 542 | -2.0 |
| GSDMB | gasdermin B | -2.0 |
| EHBP1 | EH domain binding protein 1 | -2.0 |
| CAMK2B | calcium/calmodulin-dependent protein kinase II beta | -2.0 |
| FXN | frataxin | -2.0 |
| C21orf91 | chromosome 21 open reading frame 91 | -2.0 |
| NUMB | numb homolog (Drosophila) | -2.1 |
| CROT | carnitine O-octanoyltransferase | -2.1 |
| APOA1 | apolipoprotein A-I | -2.1 |
| TBXA2R | thromboxane A2 receptor | -2.1 |
| AK3 | adenylate kinase 3 | -2.1 |
| CYP2J2 | cytochrome P450, family 2, subfamily J, polypeptide 2 | -2.1 |
| PALM3 | paralemmin 3 | -2.1 |
| IL1RL1 | interleukin 1 receptor-like 1 | -2.1 |
| C10orf116 | chromosome 10 open reading frame 116 | -2.1 |
| PHYH | phytanoyl-CoA 2-hydroxylase | -2.1 |
| LONRF3 | LON peptidase N-terminal domain and ring finger 3 | -2.1 |
| SPRY4-IT1 | SPRY4 intronic transcript 1 (non-protein coding) | -2.1 |
| KIAA1456 | KIAA1456 | -2.1 |
| SMAD1 | SMAD family member 1 | -2.1 |
| SPATS2L | spermatogenesis associated, serine-rich 2-like | -2.1 |
| STAT4 | signal transducer and activator of transcription 4 | -2.1 |
| DTX1 | deltex homolog 1 (Drosophila) | -2.1 |
| PTH1R | parathyroid hormone 1 receptor | -2.1 |
| SAMD4A | sterile alpha motif domain containing 4A | -2.1 |
| SCAF4 | SR-related CTD-associated factor 4 | -2.1 |
| APOC3 | apolipoprotein C-III | -2.1 |
| OSBP | oxysterol binding protein | -2.1 |
| ZC2HC1C | zinc finger, C2HC-type containing 1C | -2.1 |
| FRMD4A | FERM domain containing 4A | -2.1 |
| THSD4 | thrombospondin, type I, domain containing 4 | -2.1 |
| NMRK1 | nicotinamide riboside kinase 1 | -2.1 |
| CYP4F12 | cytochrome P450, family 4, subfamily F, polypeptide 12 | -2.1 |
| CDA | cytidine deaminase | -2.1 |
| GOT2 | glutamic-oxaloacetic transaminase 2, mitochondrial (aspartate aminotransferase 2) | -2.1 |
| GCGR | glucagon receptor | -2.1 |
| PRODH2 | proline dehydrogenase (oxidase) 2 | -2.1 |
| NME5 | NME/NM23 family member 5 | -2.1 |
| MTUS1 | microtubule associated tumor suppressor 1 | -2.1 |
| PDE3B | phosphodiesterase 3B, cGMP-inhibited | -2.1 |
| DNAJC25 | DnaJ (Hsp40) homolog, subfamily C , member 25 | -2.1 |
| ZFP1 | zinc finger protein 1 homolog (mouse) | -2.2 |
| NANOGNB | NANOG neighbor homeobox | -2.2 |
| NAT1 | N-acetyltransferase 1 (arylamine N-acetyltransferase) | -2.2 |
| IAPP | islet amyloid polypeptide | -2.2 |
| ALLC | allantoicase | -2.2 |
| SIK3 | SIK family kinase 3 | -2.2 |
| DACH1 | dachshund homolog 1 (Drosophila) | -2.2 |
| PROX1 | prospero homeobox 1 | -2.2 |
| CBR4 | carbonyl reductase 4 | -2.2 |
| LPIN2 | lipin 2 | -2.2 |
| ANKRD18A | ankyrin repeat domain 18A | -2.2 |
| MAP2K1 | mitogen-activated protein kinase kinase 1 | -2.2 |
| FERMT2 | fermitin family member 2 | -2.2 |
| LOC100289058 | uncharacterized LOC100289058 | -2.2 |
| TMED5 | transmembrane emp24 protein transport domain containing 5 | -2.2 |
| TRIB1 | tribbles homolog 1 (Drosophila) | -2.2 |
| SGK2 | serum/glucocorticoid regulated kinase 2 | -2.2 |
| FITM1 | fat storage-inducing transmembrane protein 1 | -2.2 |
| C11orf54 | chromosome 11 open reading frame 54 | -2.2 |
| SEC24D | SEC24 family, member D (S. cerevisiae) | -2.2 |
| SLC38A2 | solute carrier family 38, member 2 | -2.2 |
| B2M | beta-2-microglobulin | -2.2 |
| CMYA5 | cardiomyopathy associated 5 | -2.2 |
| RDH5 | retinol dehydrogenase 5 (11-cis/9-cis) | -2.2 |
| KIAA1217 | KIAA1217 | -2.2 |
| ZCCHC2 | zinc finger, CCHC domain containing 2 | -2.2 |
| SORBS1 | sorbin and SH3 domain containing 1 | -2.2 |
| MBD4 | methyl-CpG binding domain protein 4 | -2.2 |
| IER2 | immediate early response 2 | -2.2 |
| PRO2852 | uncharacterized protein PRO2852 | -2.2 |
| LINC00261 | long intergenic non-protein coding RNA 261 | -2.2 |
| IVD | isovaleryl-CoA dehydrogenase | -2.2 |
| BCL2L11 | BCL2-like 11 (apoptosis facilitator) | -2.2 |
| SLC17A9 | solute carrier family 17, member 9 | -2.2 |
| SPATA13 | spermatogenesis associated 13 | -2.2 |
| EPB41L5 | erythrocyte membrane protein band 4.1 like 5 | -2.2 |
| SLC25A16 | solute carrier family 25 (mitochondrial carrier; Graves disease autoantigen), member 16 | -2.2 |
| PCK2 | phosphoenolpyruvate carboxykinase 2 (mitochondrial) | -2.2 |
| GABARAPL3 | GABA(A) receptors associated protein like 3, pseudogene | -2.2 |
| LPP | LIM domain containing preferred translocation partner in lipoma | -2.2 |
| SLC25A20 | solute carrier family 25 (carnitine/acylcarnitine translocase), member 20 | -2.2 |
| GPR126 | G protein-coupled receptor 126 | -2.2 |
| SLC38A4 | solute carrier family 38, member 4 | -2.2 |
| MORF4L2 | mortality factor 4 like 2 | -2.2 |
| WDR1 | WD repeat domain 1 | -2.2 |
| STAT3 | signal transducer and activator of transcription 3 (acute-phase response factor) | -2.2 |
| CFLAR | CASP8 and FADD-like apoptosis regulator | -2.2 |
| KCNJ3 | potassium inwardly-rectifying channel, subfamily J, member 3 | -2.2 |
| ASGR2 | asialoglycoprotein receptor 2 | -2.2 |
| PELO | pelota homolog (Drosophila) | -2.3 |
| NFKBIZ | nuclear factor of kappa light polypeptide gene enhancer in B-cells inhibitor, zeta | -2.3 |
| CLN8 | ceroid-lipofuscinosis, neuronal 8 (epilepsy, progressive with mental retardation) | -2.3 |
| HOOK1 | hook homolog 1 (Drosophila) | -2.3 |
| GFRA1 | GDNF family receptor alpha 1 | -2.3 |
| PAPSS2 | 3'-phosphoadenosine 5'-phosphosulfate synthase 2 | -2.3 |
| INTS6 | integrator complex subunit 6 | -2.3 |
| ZNF295 | zinc finger protein 295 | -2.3 |
| CAND2 | cullin-associated and neddylation-dissociated 2 (putative) | -2.3 |
| NBPF15 | neuroblastoma breakpoint family, member 15 | -2.3 |
| BMPER | BMP binding endothelial regulator | -2.3 |
| PIK3R1 | phosphoinositide-3-kinase, regulatory subunit 1 (alpha) | -2.3 |
| NBLA00301 | Nbla00301 | -2.3 |
| LPAL2 | lipoprotein, Lp(a)-like 2, pseudogene | -2.3 |
| KLRF1 | killer cell lectin-like receptor subfamily F, member 1 | -2.3 |
| LOC100128508 | PP12100 | -2.3 |
| PELI1 | pellino E3 ubiquitin protein ligase 1 | -2.3 |
| PLSCR4 | phospholipid scramblase 4 | -2.3 |
| LOC100505570 | uncharacterized LOC100505570 | -2.3 |
| LRRN3 | leucine rich repeat neuronal 3 | -2.3 |
| PXDC1 | PX domain containing 1 | -2.3 |
| HAO1 | hydroxyacid oxidase (glycolate oxidase) 1 | -2.3 |
| FBXO21 | F-box protein 21 | -2.3 |
| SLC8A1 | solute carrier family 8 (sodium/calcium exchanger), member 1 | -2.3 |
| ACAA1 | acetyl-CoA acyltransferase 1 | -2.3 |
| SCNN1A | sodium channel, non-voltage-gated 1 alpha subunit | -2.3 |
| CFP | complement factor properdin | -2.3 |
| CHST4 | carbohydrate (N-acetylglucosamine 6-O) sulfotransferase 4 | -2.3 |
| RAB25 | RAB25, member RAS oncogene family | -2.3 |
| PPFIBP1 | PTPRF interacting protein, binding protein 1 (liprin beta 1) | -2.3 |
| WNT11 | wingless-type MMTV integration site family, member 11 | -2.3 |
| SEMA6D | sema domain, transmembrane domain (TM), and cytoplasmic domain, (semaphorin) 6D | -2.3 |
| MAT1A | methionine adenosyltransferase I, alpha | -2.3 |
| ANGPTL3 | angiopoietin-like 3 | -2.3 |
| SLC26A6 | solute carrier family 26, member 6 | -2.3 |
| PLCG2 | phospholipase C, gamma 2 (phosphatidylinositol-specific) | -2.3 |
| C4orf29 | chromosome 4 open reading frame 29 | -2.4 |
| PRRG4 | proline rich Gla (G-carboxyglutamic acid) 4 (transmembrane) | -2.4 |
| NXF3 | nuclear RNA export factor 3 | -2.4 |
| ARRB1 | arrestin, beta 1 | -2.4 |
| SHBG | sex hormone-binding globulin | -2.4 |
| JUNB | jun B proto-oncogene | -2.4 |
| GNE | glucosamine (UDP-N-acetyl)-2-epimerase/N-acetylmannosamine kinase | -2.4 |
| LOC100506974 | uncharacterized LOC100506974 | -2.4 |
| FCGR2B | Fc fragment of IgG, low affinity IIb, receptor (CD32) | -2.4 |
| PSD3 | pleckstrin and Sec7 domain containing 3 | -2.4 |
| DAPK1 | death-associated protein kinase 1 | -2.4 |
| PDE7B | phosphodiesterase 7B | -2.4 |
| IRF8 | interferon regulatory factor 8 | -2.4 |
| COX7B | cytochrome c oxidase subunit VIIb | -2.4 |
| ATOH7 | atonal homolog 7 (Drosophila) | -2.4 |
| SLC25A25 | solute carrier family 25 (mitochondrial carrier; phosphate carrier), member 25 | -2.4 |
| VIPR1 | vasoactive intestinal peptide receptor 1 | -2.4 |
| TNFRSF10D | tumor necrosis factor receptor superfamily, member 10d, decoy with truncated death domain | -2.4 |
| SLC23A2 | solute carrier family 23 (nucleobase transporters), member 2 | -2.4 |
| GPHN | gephyrin | -2.4 |
| BLNK | B-cell linker | -2.4 |
| NPHP3 | nephronophthisis 3 (adolescent) | -2.4 |
| RORA | RAR-related orphan receptor A | -2.4 |
| PLD1 | phospholipase D1, phosphatidylcholine-specific | -2.4 |
| ST3GAL6 | ST3 beta-galactoside alpha-2,3-sialyltransferase 6 | -2.4 |
| EXOC3L4 | exocyst complex component 3-like 4 | -2.4 |
| RASGEF1B | RasGEF domain family, member 1B | -2.5 |
| ACAA2 | acetyl-CoA acyltransferase 2 | -2.5 |
| DAK | dihydroxyacetone kinase 2 homolog (S. cerevisiae) | -2.5 |
| RIPK4 | receptor-interacting serine-threonine kinase 4 | -2.5 |
| ADK | adenosine kinase | -2.5 |
| GNPNAT1 | glucosamine-phosphate N-acetyltransferase 1 | -2.5 |
| KLF11 | Kruppel-like factor 11 | -2.5 |
| ABCA9 | ATP-binding cassette, sub-family A (ABC1), member 9 | -2.5 |
| CNTN4 | contactin 4 | -2.5 |
| MUT | methylmalonyl CoA mutase | -2.5 |
| SERPINA5 | serpin peptidase inhibitor, clade A (alpha-1 antiproteinase, antitrypsin), member 5 | -2.5 |
| FAM150B | family with sequence similarity 150, member B | -2.5 |
| PNPLA3 | patatin-like phospholipase domain containing 3 | -2.5 |
| PRKRA | protein kinase, interferon-inducible double stranded RNA dependent activator | -2.5 |
| DST | dystonin | -2.5 |
| RBMS3 | RNA binding motif, single stranded interacting protein 3 | -2.5 |
| ABHD2 | abhydrolase domain containing 2 | -2.5 |
| MST1 | macrophage stimulating 1 (hepatocyte growth factor-like) | -2.5 |
| RHEB | Ras homolog enriched in brain | -2.5 |
| MMAA | methylmalonic aciduria (cobalamin deficiency) cblA type | -2.5 |
| PAIP2B | poly(A) binding protein interacting protein 2B | -2.5 |
| SLC9B2 | solute carrier family 9, subfamily B (NHA2, cation proton antiporter 2), member 2 | -2.5 |
| GCDH | glutaryl-CoA dehydrogenase | -2.5 |
| PSMA5 | proteasome (prosome, macropain) subunit, alpha type, 5 | -2.5 |
| NDRG2 | NDRG family member 2 | -2.5 |
| CPT1A | carnitine palmitoyltransferase 1A (liver) | -2.5 |
| SLC31A1 | solute carrier family 31 (copper transporters), member 1 | -2.5 |
| ANKRD55 | ankyrin repeat domain 55 | -2.5 |
| TPPP2 | tubulin polymerization-promoting protein family member 2 | -2.5 |
| ECM1 | extracellular matrix protein 1 | -2.5 |
| MAN1C1 | mannosidase, alpha, class 1C, member 1 | -2.5 |
| ARMCX3 | armadillo repeat containing, X-linked 3 | -2.6 |
| SRD5A1 | steroid-5-alpha-reductase, alpha polypeptide 1 (3-oxo-5 alpha-steroid delta 4-dehydrogenase alpha 1) | -2.6 |
| GPT2 | glutamic pyruvate transaminase (alanine aminotransferase) 2 | -2.6 |
| NSUN6 | NOP2/Sun domain family, member 6 | -2.6 |
| DUSP10 | dual specificity phosphatase 10 | -2.6 |
| EVC | Ellis van Creveld syndrome | -2.6 |
| PAMR1 | peptidase domain containing associated with muscle regeneration 1 | -2.6 |
| LOC286114 | uncharacterized LOC286114 | -2.6 |
| IDNK | idnK, gluconokinase homolog (E. coli) | -2.6 |
| CPN1 | carboxypeptidase N, polypeptide 1 | -2.6 |
| HRSP12 | heat-responsive protein 12 | -2.6 |
| ACSM3 | acyl-CoA synthetase medium-chain family member 3 | -2.6 |
| SLC39A5 | solute carrier family 39 (metal ion transporter), member 5 | -2.6 |
| HES1 | hairy and enhancer of split 1, (Drosophila) | -2.6 |
| SDCBP2 | syndecan binding protein (syntenin) 2 | -2.6 |
| RAPH1 | Ras association (RalGDS/AF-6) and pleckstrin homology domains 1 | -2.6 |
| SKAP1 | src kinase associated phosphoprotein 1 | -2.6 |
| ABCG5 | ATP-binding cassette, sub-family G (WHITE), member 5 | -2.6 |
| GADD45A | growth arrest and DNA-damage-inducible, alpha | -2.6 |
| ACOX1 | acyl-CoA oxidase 1, palmitoyl | -2.6 |
| CCDC150 | coiled-coil domain containing 150 | -2.6 |
| C10orf108 | chromosome 10 open reading frame 108 | -2.6 |
| IGFALS | insulin-like growth factor binding protein, acid labile subunit | -2.6 |
| C1RL | complement component 1, r subcomponent-like | -2.6 |
| EHD3 | EH-domain containing 3 | -2.6 |
| ERRFI1 | ERBB receptor feedback inhibitor 1 | -2.7 |
| ANGPTL6 | angiopoietin-like 6 | -2.7 |
| SYNE1 | spectrin repeat containing, nuclear envelope 1 | -2.7 |
| RALGAPA2 | Ral GTPase activating protein, alpha subunit 2 (catalytic) | -2.7 |
| LOC255167 | uncharacterized LOC255167 | -2.7 |
| CDC37L1 | cell division cycle 37 homolog (S. cerevisiae)-like 1 | -2.7 |
| TCTEX1D1 | Tctex1 domain containing 1 | -2.7 |
| C8orf42 | chromosome 8 open reading frame 42 | -2.7 |
| PBLD | phenazine biosynthesis-like protein domain containing | -2.7 |
| AGPAT9 | 1-acylglycerol-3-phosphate O-acyltransferase 9 | -2.7 |
| FAM59A | family with sequence similarity 59, member A | -2.7 |
| SLC4A4 | solute carrier family 4, sodium bicarbonate cotransporter, member 4 | -2.7 |
| ST6GAL2 | ST6 beta-galactosamide alpha-2,6-sialyltranferase 2 | -2.7 |
| PPAP2B | phosphatidic acid phosphatase type 2B | -2.7 |
| PUS10 | pseudouridylate synthase 10 | -2.7 |
| LIPC | lipase, hepatic | -2.7 |
| PRPF18 | PRP18 pre-mRNA processing factor 18 homolog (S. cerevisiae) | -2.7 |
| ZNF498 | zinc finger protein 498 | -2.7 |
| PZP | pregnancy-zone protein | -2.7 |
| GIPC2 | GIPC PDZ domain containing family, member 2 | -2.7 |
| ETFDH | electron-transferring-flavoprotein dehydrogenase | -2.7 |
| ACADSB | acyl-CoA dehydrogenase, short/branched chain | -2.7 |
| MAGI1 | membrane associated guanylate kinase, WW and PDZ domain containing 1 | -2.8 |
| ESRP1 | epithelial splicing regulatory protein 1 | -2.8 |
| RPS27 | ribosomal protein S27 | -2.8 |
| COL6A6 | collagen, type VI, alpha 6 | -2.8 |
| CSAD | cysteine sulfinic acid decarboxylase | -2.8 |
| DNM3OS | DNM3 opposite strand/antisense RNA | -2.8 |
| TIAM1 | T-cell lymphoma invasion and metastasis 1 | -2.8 |
| PHGDH | phosphoglycerate dehydrogenase | -2.8 |
| MRGPRF | MAS-related GPR, member F | -2.8 |
| NR1I2 | nuclear receptor subfamily 1, group I, member 2 | -2.8 |
| C8A | complement component 8, alpha polypeptide | -2.8 |
| IL4R | interleukin 4 receptor | -2.8 |
| CISH | cytokine inducible SH2-containing protein | -2.8 |
| ABLIM3 | actin binding LIM protein family, member 3 | -2.8 |
| FXYD1 | FXYD domain containing ion transport regulator 1 | -2.8 |
| RAB27A | RAB27A, member RAS oncogene family | -2.8 |
| GNAO1 | guanine nucleotide binding protein (G protein), alpha activating activity polypeptide O | -2.8 |
| IYD | iodotyrosine deiodinase | -2.8 |
| NIPAL1 | NIPA-like domain containing 1 | -2.8 |
| TTR | transthyretin | -2.8 |
| MYO10 | myosin X | -2.8 |
| KRTCAP3 | keratinocyte associated protein 3 | -2.8 |
| FNIP2 | folliculin interacting protein 2 | -2.8 |
| MCTP2 | multiple C2 domains, transmembrane 2 | -2.8 |
| MPC1 | mitochondrial pyruvate carrier 1 | -2.8 |
| GALNT2 | UDP-N-acetyl-alpha-D-galactosamine:polypeptide N-acetylgalactosaminyltransferase 2 (GalNAc-T2) | -2.8 |
| ADAMTS13 | ADAM metallopeptidase with thrombospondin type 1 motif, 13 | -2.8 |
| SLCO1B1 | solute carrier organic anion transporter family, member 1B1 | -2.8 |
| SLC25A15 | solute carrier family 25 (mitochondrial carrier; ornithine transporter) member 15 | -2.9 |
| IL18R1 | interleukin 18 receptor 1 | -2.9 |
| GSTZ1 | glutathione S-transferase zeta 1 | -2.9 |
| PRSS8 | protease, serine, 8 | -2.9 |
| GPD1 | glycerol-3-phosphate dehydrogenase 1 (soluble) | -2.9 |
| TMPRSS2 | transmembrane protease, serine 2 | -2.9 |
| SCG5 | secretogranin V (7B2 protein) | -2.9 |
| AZGP1 | alpha-2-glycoprotein 1, zinc-binding | -2.9 |
| ACMSD | aminocarboxymuconate semialdehyde decarboxylase | -2.9 |
| GPR125 | G protein-coupled receptor 125 | -2.9 |
| DHODH | dihydroorotate dehydrogenase (quinone) | -2.9 |
| PON3 | paraoxonase 3 | -2.9 |
| TMEM56 | transmembrane protein 56 | -2.9 |
| C1R | complement component 1, r subcomponent | -2.9 |
| NAPSB | napsin B aspartic peptidase, pseudogene | -2.9 |
| KIAA1671 | KIAA1671 | -2.9 |
| NAV2 | neuron navigator 2 | -3.0 |
| ITIH4 | inter-alpha-trypsin inhibitor heavy chain family, member 4 | -3.0 |
| GOT1 | glutamic-oxaloacetic transaminase 1, soluble (aspartate aminotransferase 1) | -3.0 |
| A2M | alpha-2-macroglobulin | -3.0 |
| C6orf123 | chromosome 6 open reading frame 123 | -3.0 |
| PTGIS | prostaglandin I2 (prostacyclin) synthase | -3.0 |
| GLT1D1 | glycosyltransferase 1 domain containing 1 | -3.0 |
| GPR182 | G protein-coupled receptor 182 | -3.0 |
| ZFP36 | zinc finger protein 36, C3H type, homolog (mouse) | -3.0 |
| MFAP3L | microfibrillar-associated protein 3-like | -3.0 |
| CETP | cholesteryl ester transfer protein, plasma | -3.0 |
| LOC200772 | uncharacterized LOC200772 | -3.0 |
| CP | ceruloplasmin (ferroxidase) | -3.0 |
| CSRNP1 | cysteine-serine-rich nuclear protein 1 | -3.0 |
| DEPDC7 | DEP domain containing 7 | -3.0 |
| PHLDB2 | pleckstrin homology-like domain, family B, member 2 | -3.0 |
| PPP2R1B | protein phosphatase 2, regulatory subunit A, beta | -3.0 |
| CXCR7 | chemokine (C-X-C motif) receptor 7 | -3.0 |
| ITGA9 | integrin, alpha 9 | -3.0 |
| DLC1 | deleted in liver cancer 1 | -3.0 |
| PCSK6 | proprotein convertase subtilisin/kexin type 6 | -3.0 |
| AGBL2 | ATP/GTP binding protein-like 2 | -3.0 |
| STEAP4 | STEAP family member 4 | -3.0 |
| ZFAND5 | zinc finger, AN1-type domain 5 | -3.0 |
| LGSN | lengsin, lens protein with glutamine synthetase domain | -3.0 |
| PRR18 | proline rich 18 | -3.0 |
| AMDHD1 | amidohydrolase domain containing 1 | -3.0 |
| BCHE | butyrylcholinesterase | -3.0 |
| CTH | cystathionase (cystathionine gamma-lyase) | -3.0 |
| PLG | plasminogen | -3.0 |
| DHRS1 | dehydrogenase/reductase (SDR family) member 1 | -3.0 |
| DMGDH | dimethylglycine dehydrogenase | -3.1 |
| SCARA5 | scavenger receptor class A, member 5 (putative) | -3.1 |
| LARP1B | La ribonucleoprotein domain family, member 1B | -3.1 |
| LOC283587 | uncharacterized LOC283587 | -3.1 |
| SLC27A2 | solute carrier family 27 (fatty acid transporter), member 2 | -3.1 |
| MEG3 | maternally expressed 3 (non-protein coding) | -3.1 |
| EPB41L4A | erythrocyte membrane protein band 4.1 like 4A | -3.1 |
| ALDH8A1 | aldehyde dehydrogenase 8 family, member A1 | -3.1 |
| NREP | neuronal regeneration related protein homolog (rat) | -3.1 |
| EIF5 | eukaryotic translation initiation factor 5 | -3.1 |
| OGDHL | oxoglutarate dehydrogenase-like | -3.1 |
| SIK1 | salt-inducible kinase 1 | -3.1 |
| LY6E | lymphocyte antigen 6 complex, locus E | -3.1 |
| MASP1 | mannan-binding lectin serine peptidase 1 (C4/C2 activating component of Ra-reactive factor) | -3.1 |
| B4GALT1 | UDP-Gal:betaGlcNAc beta 1,4- galactosyltransferase, polypeptide 1 | -3.1 |
| BDH2 | 3-hydroxybutyrate dehydrogenase, type 2 | -3.1 |
| FAM13A | family with sequence similarity 13, member A | -3.2 |
| KCNJ16 | potassium inwardly-rectifying channel, subfamily J, member 16 | -3.2 |
| SORBS2 | sorbin and SH3 domain containing 2 | -3.2 |
| HOGA1 | 4-hydroxy-2-oxoglutarate aldolase 1 | -3.2 |
| SLC25A27 | solute carrier family 25, member 27 | -3.2 |
| SLC19A3 | solute carrier family 19, member 3 | -3.2 |
| CYR61 | cysteine-rich, angiogenic inducer, 61 | -3.2 |
| PRKAG2 | protein kinase, AMP-activated, gamma 2 non-catalytic subunit | -3.2 |
| CDH1 | cadherin 1, type 1, E-cadherin (epithelial) | -3.2 |
| ARHGEF26 | Rho guanine nucleotide exchange factor (GEF) 26 | -3.2 |
| ANK3 | ankyrin 3, node of Ranvier (ankyrin G) | -3.2 |
| SERPINA4 | serpin peptidase inhibitor, clade A (alpha-1 antiproteinase, antitrypsin), member 4 | -3.2 |
| RCAN1 | regulator of calcineurin 1 | -3.3 |
| SPIRE1 | spire homolog 1 (Drosophila) | -3.3 |
| ZFP3 | zinc finger protein 3 homolog (mouse) | -3.3 |
| FXYD2 | FXYD domain containing ion transport regulator 2 | -3.3 |
| GPM6A | glycoprotein M6A | -3.3 |
| TMEM30B | transmembrane protein 30B | -3.3 |
| FAM9B | family with sequence similarity 9, member B | -3.3 |
| PDK4 | pyruvate dehydrogenase kinase, isozyme 4 | -3.3 |
| ATOH8 | atonal homolog 8 (Drosophila) | -3.3 |
| SCARNA17 | small Cajal body-specific RNA 17 | -3.3 |
| GPR180 | G protein-coupled receptor 180 | -3.3 |
| TUBE1 | tubulin, epsilon 1 | -3.3 |
| STAB2 | stabilin 2 | -3.3 |
| SLC41A2 | solute carrier family 41, member 2 | -3.3 |
| PIK3C2G | phosphatidylinositol-4-phosphate 3-kinase, catalytic subunit type 2 gamma | -3.3 |
| ESRP2 | epithelial splicing regulatory protein 2 | -3.3 |
| PANK1 | pantothenate kinase 1 | -3.3 |
| C8B | complement component 8, beta polypeptide | -3.3 |
| EPB41L4B | erythrocyte membrane protein band 4.1 like 4B | -3.3 |
| MST1P9 | macrophage stimulating 1 (hepatocyte growth factor-like) pseudogene 9 | -3.4 |
| MOGAT2 | monoacylglycerol O-acyltransferase 2 | -3.4 |
| KIAA0146 | KIAA0146 | -3.4 |
| FOXO1 | forkhead box O1 | -3.4 |
| ETS2 | v-ets erythroblastosis virus E26 oncogene homolog 2 (avian) | -3.4 |
| PLIN2 | perilipin 2 | -3.4 |
| SDS | serine dehydratase | -3.4 |
| TJP2 | tight junction protein 2 | -3.4 |
| GK | glycerol kinase | -3.4 |
| MBL2 | mannose-binding lectin (protein C) 2, soluble | -3.4 |
| FLJ22763 | uncharacterized LOC401081 | -3.4 |
| NAAA | N-acylethanolamine acid amidase | -3.5 |
| PROZ | protein Z, vitamin K-dependent plasma glycoprotein | -3.5 |
| CCBE1 | collagen and calcium binding EGF domains 1 | -3.5 |
| PLIN1 | perilipin 1 | -3.5 |
| GBP1 | guanylate binding protein 1, interferon-inducible | -3.5 |
| DBH-AS1 | DBH antisense RNA 1 | -3.5 |
| GADD45G | growth arrest and DNA-damage-inducible, gamma | -3.6 |
| F11 | coagulation factor XI | -3.6 |
| TMEM154 | transmembrane protein 154 | -3.6 |
| ENO3 | enolase 3 (beta, muscle) | -3.6 |
| PALM2 | paralemmin 2 | -3.6 |
| CIDEB | cell death-inducing DFFA-like effector b | -3.6 |
| TAT | tyrosine aminotransferase | -3.6 |
| AADAT | aminoadipate aminotransferase | -3.6 |
| ACADL | acyl-CoA dehydrogenase, long chain | -3.6 |
| APBA1 | amyloid beta (A4) precursor protein-binding, family A, member 1 | -3.6 |
| SERPINB9 | serpin peptidase inhibitor, clade B (ovalbumin), member 9 | -3.6 |
| MXRA5 | matrix-remodelling associated 5 | -3.6 |
| FOSB | FBJ murine osteosarcoma viral oncogene homolog B | -3.6 |
| LONRF2 | LON peptidase N-terminal domain and ring finger 2 | -3.6 |
| LONP2 | lon peptidase 2, peroxisomal | -3.6 |
| SLC7A2 | solute carrier family 7 (cationic amino acid transporter, y+ system), member 2 | -3.7 |
| BHMT | betaine--homocysteine S-methyltransferase | -3.7 |
| DPF3 | D4, zinc and double PHD fingers, family 3 | -3.7 |
| WDR72 | WD repeat domain 72 | -3.7 |
| GLDC | glycine dehydrogenase (decarboxylating) | -3.7 |
| PITPNM3 | PITPNM family member 3 | -3.7 |
| KAZN | kazrin, periplakin interacting protein | -3.7 |
| SPATA6L | spermatogenesis associated 6-like | -3.7 |
| CPEB3 | cytoplasmic polyadenylation element binding protein 3 | -3.7 |
| ACSL1 | acyl-CoA synthetase long-chain family member 1 | -3.7 |
| CYFIP2 | cytoplasmic FMR1 interacting protein 2 | -3.7 |
| RCL1 | RNA terminal phosphate cyclase-like 1 | -3.7 |
| PKHD1 | polycystic kidney and hepatic disease 1 (autosomal recessive) | -3.7 |
| WWC1 | WW and C2 domain containing 1 | -3.7 |
| IGKC | immunoglobulin kappa constant | -3.8 |
| BACH2 | BTB and CNC homology 1, basic leucine zipper transcription factor 2 | -3.8 |
| SLC20A1 | solute carrier family 20 (phosphate transporter), member 1 | -3.8 |
| EPHB1 | EPH receptor B1 | -3.8 |
| CD14 | CD14 molecule | -3.8 |
| YPEL2 | yippee-like 2 (Drosophila) | -3.9 |
| OLFML3 | olfactomedin-like 3 | -3.9 |
| FAM149A | family with sequence similarity 149, member A | -3.9 |
| CYP4F2 | cytochrome P450, family 4, subfamily F, polypeptide 2 | -3.9 |
| ACACB | acetyl-CoA carboxylase beta | -3.9 |
| FAM134B | family with sequence similarity 134, member B | -3.9 |
| MYOM2 | myomesin (M-protein) 2, 165kDa | -3.9 |
| DUSP1 | dual specificity phosphatase 1 | -3.9 |
| IGFBP3 | insulin-like growth factor binding protein 3 | -3.9 |
| CD1D | CD1d molecule | -3.9 |
| CFHR3 | complement factor H-related 3 | -3.9 |
| KBTBD11 | kelch repeat and BTB (POZ) domain containing 11 | -4.0 |
| ABCA8 | ATP-binding cassette, sub-family A (ABC1), member 8 | -4.0 |
| SULT1E1 | sulfotransferase family 1E, estrogen-preferring, member 1 | -4.0 |
| MRC1 | mannose receptor, C type 1 | -4.0 |
| ALDH6A1 | aldehyde dehydrogenase 6 family, member A1 | -4.0 |
| SLC1A1 | solute carrier family 1 (neuronal/epithelial high affinity glutamate transporter, system Xag), member 1 | -4.0 |
| CHST9 | carbohydrate (N-acetylgalactosamine 4-0) sulfotransferase 9 | -4.0 |
| HMGCS2 | 3-hydroxy-3-methylglutaryl-CoA synthase 2 (mitochondrial) | -4.0 |
| SLC22A7 | solute carrier family 22 (organic anion transporter), member 7 | -4.1 |
| RND3 | Rho family GTPase 3 | -4.1 |
| HGFAC | HGF activator | -4.1 |
| OAT | ornithine aminotransferase | -4.2 |
| MCC | mutated in colorectal cancers | -4.2 |
| EGR1 | early growth response 1 | -4.2 |
| GRHL1 | grainyhead-like 1 (Drosophila) | -4.2 |
| LOC284801 | uncharacterized LOC284801 | -4.2 |
| MRO | maestro | -4.2 |
| SORL1 | sortilin-related receptor, L(DLR class) A repeats containing | -4.2 |
| SLC25A18 | solute carrier family 25 (glutamate carrier), member 18 | -4.2 |
| C3P1 | complement component 3 precursor pseudogene | -4.2 |
| TACSTD2 | tumor-associated calcium signal transducer 2 | -4.2 |
| FTCD | formiminotransferase cyclodeaminase | -4.3 |
| MASP2 | mannan-binding lectin serine peptidase 2 | -4.3 |
| SPATA18 | spermatogenesis associated 18 | -4.3 |
| COLEC11 | collectin sub-family member 11 | -4.3 |
| CNGA1 | cyclic nucleotide gated channel alpha 1 | -4.3 |
| RNF125 | ring finger protein 125, E3 ubiquitin protein ligase | -4.3 |
| ZG16 | zymogen granule protein 16 homolog (rat) | -4.3 |
| PPID | peptidylprolyl isomerase D | -4.3 |
| F9 | coagulation factor IX | -4.3 |
| ASS1 | argininosuccinate synthase 1 | -4.3 |
| CYP4V2 | cytochrome P450, family 4, subfamily V, polypeptide 2 | -4.3 |
| C1orf168 | chromosome 1 open reading frame 168 | -4.3 |
| UGP2 | UDP-glucose pyrophosphorylase 2 | -4.3 |
| ZGPAT | zinc finger, CCCH-type with G patch domain | -4.4 |
| FOLH1 | folate hydrolase (prostate-specific membrane antigen) 1 | -4.4 |
| ID1 | inhibitor of DNA binding 1, dominant negative helix-loop-helix protein | -4.4 |
| MBNL2 | muscleblind-like splicing regulator 2 | -4.4 |
| FABP1 | fatty acid binding protein 1, liver | -4.4 |
| TBX15 | T-box 15 | -4.4 |
| NPY1R | neuropeptide Y receptor Y1 | -4.5 |
| CYP3A5 | cytochrome P450, family 3, subfamily A, polypeptide 5 | -4.5 |
| PLAC8 | placenta-specific 8 | -4.5 |
| SLC13A5 | solute carrier family 13 (sodium-dependent citrate transporter), member 5 | -4.5 |
| TGFA | transforming growth factor, alpha | -4.5 |
| ADRA1A | adrenoceptor alpha 1A | -4.5 |
| CYP2A7 | cytochrome P450, family 2, subfamily A, polypeptide 7 | -4.5 |
| RNF165 | ring finger protein 165 | -4.5 |
| LOC100129447 | uncharacterized LOC100129447 | -4.6 |
| MT2A | metallothionein 2A | -4.6 |
| LIPG | lipase, endothelial | -4.6 |
| ANO1 | anoctamin 1, calcium activated chloride channel | -4.6 |
| BGN | biglycan | -4.6 |
| GCH1 | GTP cyclohydrolase 1 | -4.6 |
| GREM2 | gremlin 2 | -4.6 |
| SH3YL1 | SH3 domain containing, Ysc84-like 1 (S. cerevisiae) | -4.7 |
| MARCO | macrophage receptor with collagenous structure | -4.7 |
| CD5L | CD5 molecule-like | -4.7 |
| TMEM27 | transmembrane protein 27 | -4.7 |
| ATF5 | activating transcription factor 5 | -4.8 |
| LIFR | leukemia inhibitory factor receptor alpha | -4.8 |
| KLKB1 | kallikrein B, plasma (Fletcher factor) 1 | -4.8 |
| SNORA28 | small nucleolar RNA, H/ACA box 28 | -4.9 |
| SLCO4C1 | solute carrier organic anion transporter family, member 4C1 | -4.9 |
| PPP1R1A | protein phosphatase 1, regulatory (inhibitor) subunit 1A | -4.9 |
| GABRB3 | gamma-aminobutyric acid (GABA) A receptor, beta 3 | -4.9 |
| PPARGC-1α/PGC-1α | peroxisome proliferator-activated receptor gamma, coactivator 1 alpha | -4.9 |
| KDM8 | lysine (K)-specific demethylase 8 | -4.9 |
| G6PC | glucose-6-phosphatase, catalytic subunit | -5.0 |
| APOA5 | apolipoprotein A-V | -5.0 |
| SRD5A2 | steroid-5-alpha-reductase, alpha polypeptide 2 (3-oxo-5 alpha-steroid delta 4-dehydrogenase alpha 2) | -5.0 |
| C6 | complement component 6 | -5.0 |
| ANGPTL4 | angiopoietin-like 4 | -5.0 |
| ID2 | inhibitor of DNA binding 2, dominant negative helix-loop-helix protein | -5.0 |
| CYP3A43 | cytochrome P450, family 3, subfamily A, polypeptide 43 | -5.0 |
| GLYATL1 | glycine-N-acyltransferase-like 1 | -5.0 |
| CYP3A4 | cytochrome P450, family 3, subfamily A, polypeptide 4 | -5.1 |
| TDO2 | tryptophan 2,3-dioxygenase | -5.1 |
| PRG4 | proteoglycan 4 | -5.2 |
| FOLH1B | folate hydrolase 1B | -5.2 |
| AQP3 | aquaporin 3 (Gill blood group) | -5.2 |
| CYP2C19 | cytochrome P450, family 2, subfamily C, polypeptide 19 | -5.2 |
| GLYAT | glycine-N-acyltransferase | -5.2 |
| ANXA10 | annexin A10 | -5.3 |
| PLGLB1/PLGLB2 | plasminogen-like B2 | -5.4 |
| CYP3A7 | cytochrome P450, family 3, subfamily A, polypeptide 7 | -5.4 |
| RDH16 | retinol dehydrogenase 16 (all-trans) | -5.5 |
| AFM | afamin | -5.5 |
| NAMPT | nicotinamide phosphoribosyltransferase | -5.5 |
| COLEC10 | collectin sub-family member 10 (C-type lectin) | -5.6 |
| IGJ | immunoglobulin J polypeptide, linker protein for immunoglobulin alpha and mu polypeptides | -5.6 |
| RSPO3 | R-spondin 3 | -5.6 |
| N4BP2L1 | NEDD4 binding protein 2-like 1 | -5.7 |
| LOC286087 | uncharacterized LOC286087 | -5.7 |
| CLRN3 | clarin 3 | -5.8 |
| LEPR | leptin receptor | -5.8 |
| FAM110C | family with sequence similarity 110, member C | -5.9 |
| NAT2 | N-acetyltransferase 2 (arylamine N-acetyltransferase) | -5.9 |
| MT1H | metallothionein 1H | -6.0 |
| MT1E | metallothionein 1E | -6.0 |
| CYP2C18 | cytochrome P450, family 2, subfamily C, polypeptide 18 | -6.1 |
| MT1X | metallothionein 1X | -6.2 |
| HGF | hepatocyte growth factor (hepapoietin A; scatter factor) | -6.2 |
| DNASE1L3 | deoxyribonuclease I-like 3 | -6.3 |
| PDLIM5 | PDZ and LIM domain 5 | -6.3 |
| SLC10A1 | solute carrier family 10 (sodium/bile acid cotransporter family), member 1 | -6.3 |
| GNMT | glycine N-methyltransferase | -6.3 |
| GBA3 | glucosidase, beta, acid 3 (cytosolic) | -6.3 |
| CA2 | carbonic anhydrase II | -6.3 |
| AVPR1A | arginine vasopressin receptor 1A | -6.4 |
| CYP2A13/CYP2A6 | cytochrome P450, family 2, subfamily A, polypeptide 6 | -6.5 |
| LCAT | lecithin-cholesterol acyltransferase | -6.5 |
| FGFR2 | fibroblast growth factor receptor 2 | -6.6 |
| CRHBP | corticotropin releasing hormone binding protein | -6.6 |
| IGHG1 | immunoglobulin heavy constant gamma 1 (G1m marker) | -6.7 |
| SLC25A47 | solute carrier family 25, member 47 | -6.7 |
| NRG1 | neuregulin 1 | -6.7 |
| LPA | lipoprotein, Lp(a) | -6.8 |
| XDH | xanthine dehydrogenase | -6.8 |
| SLC51A | solute carrier family 51, alpha subunit | -6.8 |
| FAM65C | family with sequence similarity 65, member C | -6.9 |
| GRAMD1C | GRAM domain containing 1C | -6.9 |
| GADD45B | growth arrest and DNA-damage-inducible, beta | -6.9 |
| FOS | FBJ murine osteosarcoma viral oncogene homolog | -6.9 |
| CDHR2 | cadherin-related family member 2 | -7.0 |
| MT1G | metallothionein 1G | -7.0 |
| THBS1 | thrombospondin 1 | -7.1 |
| C8orf4 | chromosome 8 open reading frame 4 | -7.1 |
| APOF | apolipoprotein F | -7.2 |
| CYP26A1 | cytochrome P450, family 26, subfamily A, polypeptide 1 | -7.2 |
| CYP2C9 | cytochrome P450, family 2, subfamily C, polypeptide 9 | -7.2 |
| CNTN3 | contactin 3 (plasmacytoma associated) | -7.4 |
| IGHA1 | immunoglobulin heavy constant alpha 1 | -7.5 |
| PGLYRP2 | peptidoglycan recognition protein 2 | -7.6 |
| DCN | decorin | -7.6 |
| CXCL12 | chemokine (C-X-C motif) ligand 12 | -7.6 |
| BCO2 | beta-carotene oxygenase 2 | -7.7 |
| GYS2 | glycogen synthase 2 (liver) | -7.8 |
| UNC93A | unc-93 homolog A (C. elegans) | -7.9 |
| THRSP | thyroid hormone responsive | -8.0 |
| ACOT12 | acyl-CoA thioesterase 12 | -8.1 |
| GHR | growth hormone receptor | -8.1 |
| CYP4A22 | cytochrome P450, family 4, subfamily A, polypeptide 22 | -8.2 |
| KMO | kynurenine 3-monooxygenase (kynurenine 3-hydroxylase) | -8.3 |
| IGF1 | insulin-like growth factor 1 (somatomedin C) | -8.4 |
| CLEC4M | C-type lectin domain family 4, member M | -8.5 |
| MFSD2A | major facilitator superfamily domain containing 2A | -8.5 |
| MT1F | metallothionein 1F | -8.6 |
| CYP4A11 | cytochrome P450, family 4, subfamily A, polypeptide 11 | -8.8 |
| ADH4 | alcohol dehydrogenase 4 (class II), pi polypeptide | -8.9 |
| IDO2 | indoleamine 2,3-dioxygenase 2 | -9.0 |
| CYP2C8 | cytochrome P450, family 2, subfamily C, polypeptide 8 | -9.1 |
| ASPA | aspartoacylase | -9.2 |
| KCNN2 | potassium intermediate/small conductance calcium-activated channel, subfamily N, member 2 | -9.2 |
| TTC36 | tetratricopeptide repeat domain 36 | -9.6 |
| ALDOB | aldolase B, fructose-bisphosphate | -9.7 |
| CXCL2 | chemokine (C-X-C motif) ligand 2 | -9.7 |
| CYP39A1 | cytochrome P450, family 39, subfamily A, polypeptide 1 | -9.7 |
| SOCS2 | suppressor of cytokine signaling 2 | -10.0 |
| CYP2B6 | cytochrome P450, family 2, subfamily B, polypeptide 6 | -10.0 |
| FBP1 | fructose-1,6-bisphosphatase 1 | -10.0 |
| HAL | histidine ammonia-lyase | -10.1 |
| ESR1 | estrogen receptor 1 | -10.2 |
| SLCO1B3 | solute carrier organic anion transporter family, member 1B3 | -10.3 |
| VNN1 | vanin 1 | -10.4 |
| CNDP1 | carnosine dipeptidase 1 (metallopeptidase M20 family) | -10.5 |
| ODZ1 | odz, odd Oz/ten-m homolog 1 (Drosophila) | -10.7 |
| CLEC4G | C-type lectin domain family 4, member G | -11.1 |
| HAO2 | hydroxyacid oxidase 2 (long chain) | -11.4 |
| FCN3 | ficolin (collagen/fibrinogen domain containing) 3 (Hakata antigen) | -11.5 |
| MT1M | metallothionein 1M | -11.6 |
| EPCAM | epithelial cell adhesion molecule | -11.8 |
| GLS2 | glutaminase 2 (liver, mitochondrial) | -12.3 |
| GPR128 | G protein-coupled receptor 128 | -12.6 |
| SLC22A1 | solute carrier family 22 (organic cation transporter), member 1 | -12.7 |
| HHIP | hedgehog interacting protein | -12.9 |
| PCK1 | phosphoenolpyruvate carboxykinase 1 (soluble) | -13.1 |
| IL1RAP | interleukin 1 receptor accessory protein | -13.6 |
| CLEC1B | C-type lectin domain family 1, member B | -14.2 |
| AKR1D1 | aldo-keto reductase family 1, member D1 (delta 4-3-ketosteroid-5-beta-reductase) | -14.4 |
| OIT3 | oncoprotein induced transcript 3 | -14.6 |
| C9 | complement component 9 | -15.3 |
| SPP2 | secreted phosphoprotein 2, 24kDa | -16.2 |
| CYP1A2 | cytochrome P450, family 1, subfamily A, polypeptide 2 | -18.0 |
| FCN2 | ficolin (collagen/fibrinogen domain containing lectin) 2 (hucolin) | -18.7 |
| FREM2 | FRAS1 related extracellular matrix protein 2 | -18.8 |
| CXCL14 | chemokine (C-X-C motif) ligand 14 | -20.9 |
| IGF2 | insulin-like growth factor 2 (somatomedin A) | -21.6 |
| HAMP | hepcidin antimicrobial peptide | -34.3 |
| LOC100506229 | uncharacterized LOC100506229 | -38.3 |
