# Supplementary material for: Viral expression and molecular profiling in liver tissue versus microdissected hepatocytes in hepatitis B virus - associated hepatocellular carcinoma
Source: J Transl Med. 2014 Aug 21;12:230. doi: 10.1186/s12967-014-0230-1 (PMC4142136; doi:10.1186/s12967-014-0230-1)
Supplement: Additional file 4: Table S3. — Genes Differentially Expressed Between Malignant and Non-Malignant Liver Tissue. [file 12967_2014_230_MOESM4_ESM.docx]

| **Table S3.** Genes Differentially Expressed Between Malignant and Non-Malignant Liver Tissue | | |
| --- | --- | --- |
| Gene Symbol | Gene Title | Fold Change |
| AKR1B10 | aldo-keto reductase family 1, member B10 (aldose reductase) | 30.5 |
| SPINK1 | serine peptidase inhibitor, Kazal type 1 | 19.9 |
| LOC344887 | NmrA-like family domain containing 1 pseudogene | 17.6 |
| SULT1C2 | sulfotransferase family, cytosolic, 1C, member 2 | 16.9 |
| IGF2BP3 | insulin-like growth factor 2 mRNA binding protein 3 | 15.6 |
| SLC7A11 | solute carrier family 7 (anionic amino acid transporter light chain, xc- system), member 11 | 15.1 |
| SPP1 | secreted phosphoprotein 1 | 12.8 |
| CRNDE | colorectal neoplasia differentially expressed (non-protein coding) | 12.5 |
| LCN2 | lipocalin 2 | 12.3 |
| TOP2A | topoisomerase (DNA) II alpha 170kDa | 11.8 |
| NRCAM | neuronal cell adhesion molecule | 11.5 |
| ZIC2 | Zic family member 2 | 10.7 |
| TRIM16 | tripartite motif containing 16 | 10.1 |
| ANLN | anillin, actin binding protein | 9.7 |
| SFN | stratifin | 8.7 |
| RBM24 | RNA binding motif protein 24 | 8.4 |
| HMMR | hyaluronan-mediated motility receptor (RHAMM) | 8.4 |
| CCNB1 | cyclin B1 | 8.3 |
| ASPM | asp (abnormal spindle) homolog, microcephaly associated (Drosophila) | 7.6 |
| MELK | maternal embryonic leucine zipper kinase | 7.1 |
| GPC3 | glypican 3 | 6.9 |
| ROBO1 | roundabout, axon guidance receptor, homolog 1 (Drosophila) | 6.8 |
| SLC44A5 | solute carrier family 44, member 5 | 6.7 |
| PEG10 | paternally expressed 10 | 6.5 |
| NDC80 | NDC80 kinetochore complex component homolog (S. cerevisiae) | 6.4 |
| BUB1B | budding uninhibited by benzimidazoles 1 homolog beta (yeast) | 6.4 |
| CENPF | centromere protein F, 350/400kDa (mitosin) | 6.3 |
| RACGAP1 | Rac GTPase activating protein 1 | 6.0 |
| CD109 | CD109 molecule | 5.7 |
| PBK | PDZ binding kinase | 5.7 |
| DLGAP5 | discs, large (Drosophila) homolog-associated protein 5 | 5.7 |
| GCNT3 | glucosaminyl (N-acetyl) transferase 3, mucin type | 5.6 |
| CTHRC1 | collagen triple helix repeat containing 1 | 5.6 |
| CDK1 | cyclin-dependent kinase 1 | 5.6 |
| FAM72D | family with sequence similarity 72, member D | 5.6 |
| CAP2 | CAP, adenylate cyclase-associated protein, 2 (yeast) | 5.6 |
| GPR158 | G protein-coupled receptor 158 | 5.5 |
| ILDR2 | immunoglobulin-like domain containing receptor 2 | 5.5 |
| KIF14 | kinesin family member 14 | 5.5 |
| ECT2 | epithelial cell transforming sequence 2 oncogene | 5.5 |
| PI15 | peptidase inhibitor 15 | 5.2 |
| GLUL | glutamate-ammonia ligase | 5.2 |
| CDC20 | cell division cycle 20 homolog (S. cerevisiae) | 5.2 |
| DNAJC6 | DnaJ (Hsp40) homolog, subfamily C, member 6 | 5.1 |
| CA12 | carbonic anhydrase XII | 5.0 |
| FAM83D | family with sequence similarity 83, member D | 4.9 |
| IRX3 | iroquois homeobox 3 | 4.9 |
| PRC1 | protein regulator of cytokinesis 1 | 4.9 |
| KIF20A | kinesin family member 20A | 4.8 |
| VASH2 | vasohibin 2 | 4.8 |
| MRAP2 | melanocortin 2 receptor accessory protein 2 | 4.8 |
| NEK2 | NIMA (never in mitosis gene a)-related kinase 2 | 4.8 |
| KIF11 | kinesin family member 11 | 4.7 |
| GJC1 | gap junction protein, gamma 1, 45kDa | 4.7 |
| PRR11 | proline rich 11 | 4.6 |
| ACSL4 | acyl-CoA synthetase long-chain family member 4 | 4.6 |
| FGF13 | fibroblast growth factor 13 | 4.6 |
| GINS1 | GINS complex subunit 1 (Psf1 homolog) | 4.6 |
| ZIC1 | Zic family member 1 | 4.5 |
| FLJ39632 | uncharacterized LOC642477 | 4.5 |
| E2F7 | E2F transcription factor 7 | 4.5 |
| DYNC1I1 | dynein, cytoplasmic 1, intermediate chain 1 | 4.5 |
| CCNB2 | cyclin B2 | 4.5 |
| SMPX | small muscle protein, X-linked | 4.5 |
| RRM2 | ribonucleotide reductase M2 | 4.4 |
| MAP2 | microtubule-associated protein 2 | 4.4 |
| TTC39A | tetratricopeptide repeat domain 39A | 4.4 |
| LEF1 | lymphoid enhancer-binding factor 1 | 4.3 |
| BIRC5 | baculoviral IAP repeat containing 5 | 4.2 |
| NEB | nebulin | 4.2 |
| TRIM55 | tripartite motif containing 55 | 4.2 |
| NCAPG | non-SMC condensin I complex, subunit G | 4.2 |
| CCDC34 | coiled-coil domain containing 34 | 4.2 |
| TKT | transketolase | 4.1 |
| CHRM3 | cholinergic receptor, muscarinic 3 | 4.1 |
| DTL | denticleless E3 ubiquitin protein ligase homolog (Drosophila) | 4.1 |
| EML6 | echinoderm microtubule associated protein like 6 | 4.1 |
| LOC100506403 | uncharacterized LOC100506403 | 4.0 |
| EDIL3 | EGF-like repeats and discoidin I-like domains 3 | 4.0 |
| LPL | lipoprotein lipase | 4.0 |
| COL15A1 | collagen, type XV, alpha 1 | 4.0 |
| NUSAP1 | nucleolar and spindle associated protein 1 | 3.9 |
| TXNRD1 | thioredoxin reductase 1 | 3.9 |
| DNAH12 | dynein, axonemal, heavy chain 12 | 3.9 |
| PRKAA2 | protein kinase, AMP-activated, alpha 2 catalytic subunit | 3.9 |
| ITGA2 | integrin, alpha 2 (CD49B, alpha 2 subunit of VLA-2 receptor) | 3.8 |
| MAD2L1 | MAD2 mitotic arrest deficient-like 1 (yeast) | 3.8 |
| SRXN1 | sulfiredoxin 1 | 3.8 |
| BCAT1 | branched chain amino-acid transaminase 1, cytosolic | 3.8 |
| GPX2 | glutathione peroxidase 2 (gastrointestinal) | 3.7 |
| FOXM1 | forkhead box M1 | 3.7 |
| KIAA1244 | KIAA1244 | 3.7 |
| AURKA | aurora kinase A | 3.6 |
| C12orf75 | chromosome 12 open reading frame 75 | 3.6 |
| RAD51AP1 | RAD51 associated protein 1 | 3.6 |
| AKR1C3 | aldo-keto reductase family 1, member C3 (3-alpha hydroxysteroid dehydrogenase, type II) | 3.6 |
| HIST1H3A | histone cluster 1, H3a | 3.5 |
| MLF1IP | MLF1 interacting protein | 3.5 |
| LOC389834 | ankyrin repeat domain 57 pseudogene | 3.5 |
| CDCA3 | cell division cycle associated 3 | 3.5 |
| KIAA0101 | KIAA0101 | 3.5 |
| KIF4A | kinesin family member 4A | 3.5 |
| RRAGD | Ras-related GTP binding D | 3.5 |
| CENPK | centromere protein K | 3.5 |
| FANCD2 | Fanconi anemia, complementation group D2 | 3.4 |
| SGOL2 | shugoshin-like 2 (S. pombe) | 3.4 |
| AKR1C1/AKR1C2 | aldo-keto reductase family 1, member C2 (dihydrodiol dehydrogenase 2; bile acid binding protein; 3-alpha hydroxysteroid dehydrogenase, type III) | 3.4 |
| FLVCR1 | feline leukemia virus subgroup C cellular receptor 1 | 3.4 |
| STEAP2 | STEAP family member 2, metalloreductase | 3.3 |
| EID3 | EP300 interacting inhibitor of differentiation 3 | 3.3 |
| CDKN3 | cyclin-dependent kinase inhibitor 3 | 3.3 |
| TP53I3 | tumor protein p53 inducible protein 3 | 3.3 |
| ASPH | aspartate beta-hydroxylase | 3.3 |
| IGSF3 | immunoglobulin superfamily, member 3 | 3.3 |
| FABP5 | fatty acid binding protein 5 (psoriasis-associated) | 3.2 |
| CCNE2 | cyclin E2 | 3.2 |
| CENPW | centromere protein W | 3.2 |
| ZWINT | ZW10 interactor | 3.2 |
| EFCAB2 | EF-hand calcium binding domain 2 | 3.2 |
| CCNA2 | cyclin A2 | 3.2 |
| C6orf62 | chromosome 6 open reading frame 62 | 3.1 |
| IQGAP3 | IQ motif containing GTPase activating protein 3 | 3.1 |
| SQSTM1 | sequestosome 1 | 3.1 |
| DLG5 | discs, large homolog 5 (Drosophila) | 3.1 |
| MECOM | MDS1 and EVI1 complex locus | 3.1 |
| PIR | pirin (iron-binding nuclear protein) | 3.0 |
| SP5 | Sp5 transcription factor | 3.0 |
| EZH2 | enhancer of zeste homolog 2 (Drosophila) | 3.0 |
| ITGA6 | integrin, alpha 6 | 3.0 |
| LOC401068 | uncharacterized LOC401068 | 3.0 |
| CDKN2C | cyclin-dependent kinase inhibitor 2C (p18, inhibits CDK4) | 3.0 |
| PTTG1 | pituitary tumor-transforming 1 | 3.0 |
| STXBP6 | syntaxin binding protein 6 (amisyn) | 2.9 |
| UHRF1 | ubiquitin-like with PHD and ring finger domains 1 | 2.9 |
| RBM15 | RNA binding motif protein 15 | 2.9 |
| C11orf93 | chromosome 11 open reading frame 93 | 2.9 |
| KIAA1199 | KIAA1199 | 2.9 |
| ENAH | enabled homolog (Drosophila) | 2.9 |
| SPATS2 | spermatogenesis associated, serine-rich 2 | 2.9 |
| CDKN2B | cyclin-dependent kinase inhibitor 2B (p15, inhibits CDK4) | 2.9 |
| TRIM59 | tripartite motif containing 59 | 2.9 |
| PSPH | phosphoserine phosphatase | 2.9 |
| GNAI1 | guanine nucleotide binding protein (G protein), alpha inhibiting activity polypeptide 1 | 2.9 |
| GNAL | guanine nucleotide binding protein (G protein), alpha activating activity polypeptide, olfactory type | 2.8 |
| CDC7 | cell division cycle 7 homolog (S. cerevisiae) | 2.8 |
| SPON2 | spondin 2, extracellular matrix protein | 2.8 |
| TPX2 | TPX2, microtubule-associated, homolog (Xenopus laevis) | 2.8 |
| STEAP1 | six transmembrane epithelial antigen of the prostate 1 | 2.8 |
| CEP41 | centrosomal protein 41kDa | 2.8 |
| MKI67 | antigen identified by monoclonal antibody Ki-67 | 2.8 |
| FIGNL1 | fidgetin-like 1 | 2.8 |
| ARHGAP44 | Rho GTPase activating protein 44 | 2.8 |
| SLC38A6 | solute carrier family 38, member 6 | 2.7 |
| CNKSR2 | connector enhancer of kinase suppressor of Ras 2 | 2.7 |
| TRIM45 | tripartite motif containing 45 | 2.7 |
| SAC3D1 | SAC3 domain containing 1 | 2.7 |
| C1orf85 | chromosome 1 open reading frame 85 | 2.7 |
| C1orf43 | chromosome 1 open reading frame 43 | 2.7 |
| SMC2 | structural maintenance of chromosomes 2 | 2.7 |
| CDKN2A | cyclin-dependent kinase inhibitor 2A | 2.7 |
| LOC100507316 | uncharacterized LOC100507316 | 2.6 |
| RGS5 | regulator of G-protein signaling 5 | 2.6 |
| CCDC99 | coiled-coil domain containing 99 | 2.6 |
| ZBTB41 | zinc finger and BTB domain containing 41 | 2.6 |
| APOBEC3B | apolipoprotein B mRNA editing enzyme, catalytic polypeptide-like 3B | 2.6 |
| MATR3 | matrin 3 | 2.6 |
| SH3RF2 | SH3 domain containing ring finger 2 | 2.6 |
| ASAP2 | ArfGAP with SH3 domain, ankyrin repeat and PH domain 2 | 2.6 |
| RNF43 | ring finger protein 43 | 2.6 |
| ZNF232 | zinc finger protein 232 | 2.6 |
| BOP1 | block of proliferation 1 | 2.6 |
| PFKFB2 | 6-phosphofructo-2-kinase/fructose-2,6-biphosphatase 2 | 2.6 |
| SDCBP2-AS1 | SDCBP2 antisense RNA 1 | 2.5 |
| MTHFD1L | methylenetetrahydrofolate dehydrogenase (NADP+ dependent) 1-like | 2.5 |
| PRKDC | protein kinase, DNA-activated, catalytic polypeptide | 2.5 |
| HS2ST1 | heparan sulfate 2-O-sulfotransferase 1 | 2.5 |
| HTATIP2 | HIV-1 Tat interactive protein 2, 30kDa | 2.5 |
| TBC1D16 | TBC1 domain family, member 16 | 2.5 |
| S100A10 | S100 calcium binding protein A10 | 2.5 |
| TUBG1 | tubulin, gamma 1 | 2.5 |
| EME1 | essential meiotic endonuclease 1 homolog 1 (S. pombe) | 2.5 |
| MSH5 | mutS homolog 5 (E. coli) | 2.5 |
| DNM3 | dynamin 3 | 2.5 |
| STMN1 | stathmin 1 | 2.5 |
| ZNF623 | zinc finger protein 623 | 2.5 |
| RAB3B | RAB3B, member RAS oncogene family | 2.4 |
| AGBL3 | ATP/GTP binding protein-like 3 | 2.4 |
| ZKSCAN3 | zinc finger with KRAB and SCAN domains 3 | 2.4 |
| PTGFRN | prostaglandin F2 receptor negative regulator | 2.4 |
| HIST1H2AC | histone cluster 1, H2ac | 2.4 |
| KITLG | KIT ligand | 2.4 |
| SERPINI1 | serpin peptidase inhibitor, clade I (neuroserpin), member 1 | 2.4 |
| DUT | deoxyuridine triphosphatase | 2.4 |
| PLCB1 | phospholipase C, beta 1 (phosphoinositide-specific) | 2.4 |
| SLC52A2 | solute carrier family 52, riboflavin transporter, member 2 | 2.4 |
| NCAPD2 | non-SMC condensin I complex, subunit D2 | 2.4 |
| CDK5RAP2 | CDK5 regulatory subunit associated protein 2 | 2.4 |
| FEN1 | flap structure-specific endonuclease 1 | 2.4 |
| CKAP4 | cytoskeleton-associated protein 4 | 2.4 |
| KPNA2 | karyopherin alpha 2 (RAG cohort 1, importin alpha 1) | 2.4 |
| DTNA | dystrobrevin, alpha | 2.4 |
| TOMM40L | translocase of outer mitochondrial membrane 40 homolog (yeast)-like | 2.3 |
| PIEZO2 | piezo-type mechanosensitive ion channel component 2 | 2.3 |
| SPA17 | sperm autoantigenic protein 17 | 2.3 |
| UNKL | unkempt homolog (Drosophila)-like | 2.3 |
| CKAP2 | cytoskeleton associated protein 2 | 2.3 |
| MTMR11 | myotubularin related protein 11 | 2.3 |
| CPD | carboxypeptidase D | 2.3 |
| TMEM64 | transmembrane protein 64 | 2.3 |
| P4HA2 | prolyl 4-hydroxylase, alpha polypeptide II | 2.3 |
| PCDH17 | protocadherin 17 | 2.3 |
| TIGD1 | tigger transposable element derived 1 | 2.3 |
| SACS | spastic ataxia of Charlevoix-Saguenay (sacsin) | 2.3 |
| CTSC | cathepsin C | 2.3 |
| PODXL | podocalyxin-like | 2.3 |
| FERMT1 | fermitin family member 1 | 2.3 |
| PCSK5 | proprotein convertase subtilisin/kexin type 5 | 2.3 |
| TUBB2A | tubulin, beta 2A class IIa | 2.3 |
| BAIAP2-AS1 | BAIAP2 antisense RNA 1 | 2.3 |
| TRIM6 | tripartite motif containing 6 | 2.3 |
| ABCB6 | ATP-binding cassette, sub-family B (MDR/TAP), member 6 | 2.3 |
| FBXO32 | F-box protein 32 | 2.2 |
| CCDC88A | coiled-coil domain containing 88A | 2.2 |
| MLEC | malectin | 2.2 |
| ZNF280C | zinc finger protein 280C | 2.2 |
| SLC7A6 | solute carrier family 7 (amino acid transporter light chain, y+L system), member 6 | 2.2 |
| PLCE1 | phospholipase C, epsilon 1 | 2.2 |
| GGPS1 | geranylgeranyl diphosphate synthase 1 | 2.2 |
| SQLE | squalene epoxidase | 2.2 |
| OSBPL3 | oxysterol binding protein-like 3 | 2.2 |
| H2AFX | H2A histone family, member X | 2.2 |
| PTGR1 | prostaglandin reductase 1 | 2.2 |
| SLC26A2 | solute carrier family 26 (sulfate transporter), member 2 | 2.2 |
| KIF3A | kinesin family member 3A | 2.2 |
| HOXD8 | homeobox D8 | 2.2 |
| THY1 | Thy-1 cell surface antigen | 2.2 |
| ATAD2 | ATPase family, AAA domain containing 2 | 2.2 |
| ANXA2 | annexin A2 | 2.2 |
| B4GALT6 | UDP-Gal:betaGlcNAc beta 1,4- galactosyltransferase, polypeptide 6 | 2.2 |
| FSD1L | fibronectin type III and SPRY domain containing 1-like | 2.2 |
| COL5A2 | collagen, type V, alpha 2 | 2.2 |
| CCDC113 | coiled-coil domain containing 113 | 2.2 |
| EBF1 | early B-cell factor 1 | 2.2 |
| LINC00094 | long intergenic non-protein coding RNA 94 | 2.2 |
| TNFSF4 | tumor necrosis factor (ligand) superfamily, member 4 | 2.1 |
| RASAL2 | RAS protein activator like 2 | 2.1 |
| ANKRD27 | ankyrin repeat domain 27 (VPS9 domain) | 2.1 |
| TAF1A | TATA box binding protein (TBP)-associated factor, RNA polymerase I, A, 48kDa | 2.1 |
| ETV1 | ets variant 1 | 2.1 |
| GM2A | GM2 ganglioside activator | 2.1 |
| NOV | nephroblastoma overexpressed | 2.1 |
| PTP4A3 | protein tyrosine phosphatase type IVA, member 3 | 2.1 |
| TXN | thioredoxin | 2.1 |
| AIM1L | absent in melanoma 1-like | 2.1 |
| ARHGAP18 | Rho GTPase activating protein 18 | 2.1 |
| AGPAT4 | 1-acylglycerol-3-phosphate O-acyltransferase 4 (lysophosphatidic acid acyltransferase, delta) | 2.1 |
| ABCC5 | ATP-binding cassette, sub-family C (CFTR/MRP), member 5 | 2.1 |
| C7orf29 | chromosome 7 open reading frame 29 | 2.1 |
| PSRC1 | proline/serine-rich coiled-coil 1 | 2.1 |
| CENPJ | centromere protein J | 2.1 |
| STRA13 | stimulated by retinoic acid 13 homolog (mouse) | 2.1 |
| THSD7A | thrombospondin, type I, domain containing 7A | 2.1 |
| ZNF703 | zinc finger protein 703 | 2.1 |
| SRGAP2 | SLIT-ROBO Rho GTPase activating protein 2 | 2.1 |
| COG2 | component of oligomeric golgi complex 2 | 2.1 |
| CPPED1 | calcineurin-like phosphoesterase domain containing 1 | 2.1 |
| GPATCH2 | G patch domain containing 2 | 2.1 |
| PTK2 | PTK2 protein tyrosine kinase 2 | 2.1 |
| C5orf54 | chromosome 5 open reading frame 54 | 2.1 |
| UGGT1 | UDP-glucose glycoprotein glucosyltransferase 1 | 2.1 |
| UGDH | UDP-glucose 6-dehydrogenase | 2.1 |
| UGT1A6 | UDP glucuronosyltransferase 1 family, polypeptide A6 | 2.1 |
| KIAA1462 | KIAA1462 | 2.1 |
| ITGA7 | integrin, alpha 7 | 2.1 |
| LAMC1 | laminin, gamma 1 (formerly LAMB2) | 2.0 |
| DIEXF | digestive organ expansion factor homolog (zebrafish) | 2.0 |
| HIST2H2BE | histone cluster 2, H2be | 2.0 |
| TMEM164 | transmembrane protein 164 | 2.0 |
| C9orf16 | chromosome 9 open reading frame 16 | 2.0 |
| SUCO | SUN domain containing ossification factor | 2.0 |
| SLC40A1 | solute carrier family 40 (iron-regulated transporter), member 1 | 2.0 |
| USP6NL | USP6 N-terminal like | -2.0 |
| C4orf19 | chromosome 4 open reading frame 19 | -2.0 |
| C10orf108 | chromosome 10 open reading frame 108 | -2.0 |
| HAAO | 3-hydroxyanthranilate 3,4-dioxygenase | -2.0 |
| RORA | RAR-related orphan receptor A | -2.0 |
| IPCEF1 | interaction protein for cytohesin exchange factors 1 | -2.0 |
| C1QTNF7 | C1q and tumor necrosis factor related protein 7 | -2.0 |
| PUS10 | pseudouridylate synthase 10 | -2.0 |
| ACADM | acyl-CoA dehydrogenase, C-4 to C-12 straight chain | -2.0 |
| BTD | biotinidase | -2.0 |
| TREH | trehalase (brush-border membrane glycoprotein) | -2.0 |
| TMEM86B | transmembrane protein 86B | -2.0 |
| NIPAL1 | NIPA-like domain containing 1 | -2.0 |
| NR3C2 | nuclear receptor subfamily 3, group C, member 2 | -2.0 |
| CNTLN | centlein, centrosomal protein | -2.0 |
| FDX1 | ferredoxin 1 | -2.0 |
| RDH5 | retinol dehydrogenase 5 (11-cis/9-cis) | -2.0 |
| SELP | selectin P (granule membrane protein 140kDa, antigen CD62) | -2.0 |
| HDAC6 | histone deacetylase 6 | -2.0 |
| RAPGEF5 | Rap guanine nucleotide exchange factor (GEF) 5 | -2.0 |
| FXN | frataxin | -2.0 |
| RAB17 | RAB17, member RAS oncogene family | -2.0 |
| KIAA1671 | KIAA1671 | -2.0 |
| KLF10 | Kruppel-like factor 10 | -2.0 |
| ABLIM3 | actin binding LIM protein family, member 3 | -2.0 |
| NFKBIZ | nuclear factor of kappa light polypeptide gene enhancer in B-cells inhibitor, zeta | -2.0 |
| SLC25A16 | solute carrier family 25 (mitochondrial carrier; Graves disease autoantigen), member 16 | -2.0 |
| SLC37A4 | solute carrier family 37 (glucose-6-phosphate transporter), member 4 | -2.0 |
| TBXA2R | thromboxane A2 receptor | -2.1 |
| LINC00261 | long intergenic non-protein coding RNA 261 | -2.1 |
| PER1 | period homolog 1 (Drosophila) | -2.1 |
| SMOC1 | SPARC related modular calcium binding 1 | -2.1 |
| PELO | pelota homolog (Drosophila) | -2.1 |
| EPHB1 | EPH receptor B1 | -2.1 |
| LOC286114 | uncharacterized LOC286114 | -2.1 |
| SYT9 | synaptotagmin IX | -2.1 |
| STOM | stomatin | -2.1 |
| EVC | Ellis van Creveld syndrome | -2.1 |
| C1RL | complement component 1, r subcomponent-like | -2.1 |
| LY9 | lymphocyte antigen 9 | -2.1 |
| CROT | carnitine O-octanoyltransferase | -2.1 |
| TXNL1 | thioredoxin-like 1 | -2.1 |
| FAM82A1 | family with sequence similarity 82, member A1 | -2.1 |
| MAP2K1 | mitogen-activated protein kinase kinase 1 | -2.1 |
| PIK3R1 | phosphoinositide-3-kinase, regulatory subunit 1 (alpha) | -2.1 |
| RASGEF1B | RasGEF domain family, member 1B | -2.1 |
| SLC28A3 | solute carrier family 28 (sodium-coupled nucleoside transporter), member 3 | -2.1 |
| LOC100505570 | uncharacterized LOC100505570 | -2.1 |
| CDA | cytidine deaminase | -2.1 |
| DCPS | decapping enzyme, scavenger | -2.1 |
| SHMT1 | serine hydroxymethyltransferase 1 (soluble) | -2.1 |
| PSMA5 | proteasome (prosome, macropain) subunit, alpha type, 5 | -2.1 |
| IVD | isovaleryl-CoA dehydrogenase | -2.1 |
| ENDOG | endonuclease G | -2.1 |
| C14orf182 | chromosome 14 open reading frame 182 | -2.1 |
| GLIS3 | GLIS family zinc finger 3 | -2.1 |
| MMAA | methylmalonic aciduria (cobalamin deficiency) cblA type | -2.1 |
| ARRB1 | arrestin, beta 1 | -2.1 |
| ATP5G3 | ATP synthase, H+ transporting, mitochondrial Fo complex, subunit C3 (subunit 9) | -2.1 |
| PRKAG2 | protein kinase, AMP-activated, gamma 2 non-catalytic subunit | -2.1 |
| LURAP1L | leucine rich adaptor protein 1-like | -2.1 |
| PRODH2 | proline dehydrogenase (oxidase) 2 | -2.1 |
| SPRY2 | sprouty homolog 2 (Drosophila) | -2.1 |
| IGLJ3 | immunoglobulin lambda joining 3 | -2.1 |
| DTX1 | deltex homolog 1 (Drosophila) | -2.1 |
| NR4A1 | nuclear receptor subfamily 4, group A, member 1 | -2.1 |
| CXCR7 | chemokine (C-X-C motif) receptor 7 | -2.1 |
| EMR1 | egf-like module containing, mucin-like, hormone receptor-like 1 | -2.1 |
| NAV2 | neuron navigator 2 | -2.1 |
| NBPF15 | neuroblastoma breakpoint family, member 15 | -2.1 |
| TMEM220 | transmembrane protein 220 | -2.1 |
| HOOK1 | hook homolog 1 (Drosophila) | -2.1 |
| MARC2 | mitochondrial amidoxime reducing component 2 | -2.1 |
| NR2F2-AS1 | NR2F2 antisense RNA 1 | -2.1 |
| F8 | coagulation factor VIII, procoagulant component | -2.1 |
| GABARAPL3 | GABA(A) receptors associated protein like 3, pseudogene | -2.1 |
| MEG3 | maternally expressed 3 (non-protein coding) | -2.2 |
| LOC100289058 | uncharacterized LOC100289058 | -2.2 |
| LONRF3 | LON peptidase N-terminal domain and ring finger 3 | -2.2 |
| GALNT2 | UDP-N-acetyl-alpha-D-galactosamine:polypeptide N-acetylgalactosaminyltransferase 2 (GalNAc-T2) | -2.2 |
| UAP1 | UDP-N-acteylglucosamine pyrophosphorylase 1 | -2.2 |
| SAMD4A | sterile alpha motif domain containing 4A | -2.2 |
| GGT5 | gamma-glutamyltransferase 5 | -2.2 |
| PPAPDC1A | phosphatidic acid phosphatase type 2 domain containing 1A | -2.2 |
| ERLIN1 | ER lipid raft associated 1 | -2.2 |
| ATP11C | ATPase, class VI, type 11C | -2.2 |
| SATB1 | SATB homeobox 1 | -2.2 |
| ADK | adenosine kinase | -2.2 |
| KLRB1 | killer cell lectin-like receptor subfamily B, member 1 | -2.2 |
| NPHP3 | nephronophthisis 3 (adolescent) | -2.2 |
| ABHD15 | abhydrolase domain containing 15 | -2.2 |
| PCOLCE | procollagen C-endopeptidase enhancer | -2.2 |
| AVPI1 | arginine vasopressin-induced 1 | -2.2 |
| IL4R | interleukin 4 receptor | -2.2 |
| PXDC1 | PX domain containing 1 | -2.2 |
| DAPK1 | death-associated protein kinase 1 | -2.2 |
| LOC692247 | uncharacterized LOC692247 | -2.2 |
| PSAT1 | phosphoserine aminotransferase 1 | -2.2 |
| B4GALT1 | UDP-Gal:betaGlcNAc beta 1,4- galactosyltransferase, polypeptide 1 | -2.2 |
| BLNK | B-cell linker | -2.2 |
| MARC1 | mitochondrial amidoxime reducing component 1 | -2.2 |
| ST6GAL1 | ST6 beta-galactosamide alpha-2,6-sialyltranferase 1 | -2.2 |
| VMO1 | vitelline membrane outer layer 1 homolog (chicken) | -2.2 |
| TDRD6 | tudor domain containing 6 | -2.2 |
| GLUD2 | glutamate dehydrogenase 2 | -2.2 |
| STARD5 | StAR-related lipid transfer (START) domain containing 5 | -2.2 |
| GPR180 | G protein-coupled receptor 180 | -2.2 |
| FAT4 | FAT tumor suppressor homolog 4 (Drosophila) | -2.2 |
| EXOC3L4 | exocyst complex component 3-like 4 | -2.2 |
| KCNJ3 | potassium inwardly-rectifying channel, subfamily J, member 3 | -2.2 |
| FAH | fumarylacetoacetate hydrolase (fumarylacetoacetase) | -2.2 |
| C11orf54 | chromosome 11 open reading frame 54 | -2.3 |
| TMC4 | transmembrane channel-like 4 | -2.3 |
| CISH | cytokine inducible SH2-containing protein | -2.3 |
| ADAMTSL2 | ADAMTS-like 2 | -2.3 |
| SKAP1 | src kinase associated phosphoprotein 1 | -2.3 |
| DGAT2 | diacylglycerol O-acyltransferase 2 | -2.3 |
| RNASE4 | ribonuclease, RNase A family, 4 | -2.3 |
| RAPH1 | Ras association (RalGDS/AF-6) and pleckstrin homology domains 1 | -2.3 |
| KLF11 | Kruppel-like factor 11 | -2.3 |
| NR1I2 | nuclear receptor subfamily 1, group I, member 2 | -2.3 |
| GABARAPL1 | GABA(A) receptor-associated protein like 1 | -2.3 |
| IGKV1-5 | immunoglobulin kappa variable 1-5 | -2.3 |
| SLC25A25 | solute carrier family 25 (mitochondrial carrier; phosphate carrier), member 25 | -2.3 |
| ZFP3 | zinc finger protein 3 homolog (mouse) | -2.3 |
| BAI3 | brain-specific angiogenesis inhibitor 3 | -2.3 |
| GLT1D1 | glycosyltransferase 1 domain containing 1 | -2.3 |
| SYNE1 | spectrin repeat containing, nuclear envelope 1 | -2.3 |
| GLUD1 | glutamate dehydrogenase 1 | -2.3 |
| CAT | catalase | -2.3 |
| FAHD2A | fumarylacetoacetate hydrolase domain containing 2A | -2.3 |
| STAT4 | signal transducer and activator of transcription 4 | -2.3 |
| GPHN | gephyrin | -2.3 |
| COX7B | cytochrome c oxidase subunit VIIb | -2.3 |
| LOC100128252 | uncharacterized LOC100128252 | -2.3 |
| PBLD | phenazine biosynthesis-like protein domain containing | -2.3 |
| GNE | glucosamine (UDP-N-acetyl)-2-epimerase/N-acetylmannosamine kinase | -2.3 |
| RPS27 | ribosomal protein S27 | -2.3 |
| GBP1 | guanylate binding protein 1, interferon-inducible | -2.3 |
| QDPR | quinoid dihydropteridine reductase | -2.3 |
| ABHD2 | abhydrolase domain containing 2 | -2.3 |
| SUCLG2 | succinate-CoA ligase, GDP-forming, beta subunit | -2.3 |
| FCRL3 | Fc receptor-like 3 | -2.3 |
| FAM46C | family with sequence similarity 46, member C | -2.3 |
| F3 | coagulation factor III (thromboplastin, tissue factor) | -2.3 |
| NXF3 | nuclear RNA export factor 3 | -2.3 |
| ADRA1A | adrenoceptor alpha 1A | -2.3 |
| LONP2 | lon peptidase 2, peroxisomal | -2.3 |
| SORBS2 | sorbin and SH3 domain containing 2 | -2.3 |
| SPINT2 | serine peptidase inhibitor, Kunitz type, 2 | -2.3 |
| PTP4A1 | protein tyrosine phosphatase type IVA, member 1 | -2.4 |
| TRIB1 | tribbles homolog 1 (Drosophila) | -2.4 |
| PXMP2 | peroxisomal membrane protein 2, 22kDa | -2.4 |
| IYD | iodotyrosine deiodinase | -2.4 |
| FAM176A | family with sequence similarity 176, member A | -2.4 |
| PLSCR4 | phospholipid scramblase 4 | -2.4 |
| SLC23A2 | solute carrier family 23 (nucleobase transporters), member 2 | -2.4 |
| GPRASP1 | G protein-coupled receptor associated sorting protein 1 | -2.4 |
| SLC4A4 | solute carrier family 4, sodium bicarbonate cotransporter, member 4 | -2.4 |
| ARAP2 | ArfGAP with RhoGAP domain, ankyrin repeat and PH domain 2 | -2.4 |
| SLC31A1 | solute carrier family 31 (copper transporters), member 1 | -2.4 |
| TIAM1 | T-cell lymphoma invasion and metastasis 1 | -2.4 |
| CREM | cAMP responsive element modulator | -2.4 |
| mir-214 | microRNA 214 | -2.4 |
| SLC38A2 | solute carrier family 38, member 2 | -2.4 |
| ORM1 | orosomucoid 1 | -2.4 |
| C2orf40 | chromosome 2 open reading frame 40 | -2.4 |
| DMD | dystrophin | -2.4 |
| ALDH2 | aldehyde dehydrogenase 2 family (mitochondrial) | -2.4 |
| IER2 | immediate early response 2 | -2.4 |
| SLC39A5 | solute carrier family 39 (metal ion transporter), member 5 | -2.4 |
| YPEL2 | yippee-like 2 (Drosophila) | -2.4 |
| LRRN3 | leucine rich repeat neuronal 3 | -2.4 |
| UGP2 | UDP-glucose pyrophosphorylase 2 | -2.4 |
| CFD | complement factor D (adipsin) | -2.4 |
| KLF9 | Kruppel-like factor 9 | -2.4 |
| SLC25A27 | solute carrier family 25, member 27 | -2.4 |
| KLRC4-KLRK1/KLRK1 | killer cell lectin-like receptor subfamily K, member 1 | -2.4 |
| MFAP4 | microfibrillar-associated protein 4 | -2.4 |
| PAPSS2 | 3'-phosphoadenosine 5'-phosphosulfate synthase 2 | -2.4 |
| GOT2 | glutamic-oxaloacetic transaminase 2, mitochondrial (aspartate aminotransferase 2) | -2.4 |
| TNFRSF17 | tumor necrosis factor receptor superfamily, member 17 | -2.4 |
| ACAT1 | acetyl-CoA acetyltransferase 1 | -2.4 |
| DHODH | dihydroorotate dehydrogenase (quinone) | -2.5 |
| IL18RAP | interleukin 18 receptor accessory protein | -2.5 |
| BMPER | BMP binding endothelial regulator | -2.5 |
| CBS | cystathionine-beta-synthase | -2.5 |
| MTHFD1 | methylenetetrahydrofolate dehydrogenase (NADP+ dependent) 1, methenyltetrahydrofolate cyclohydrolase, formyltetrahydrofolate synthetase | -2.5 |
| TMEM200C | transmembrane protein 200C | -2.5 |
| CDKN1C | cyclin-dependent kinase inhibitor 1C (p57, Kip2) | -2.5 |
| PLD1 | phospholipase D1, phosphatidylcholine-specific | -2.5 |
| CD302 | CD302 molecule | -2.5 |
| ALLC | allantoicase | -2.5 |
| PAH | phenylalanine hydroxylase | -2.5 |
| ART4 | ADP-ribosyltransferase 4 (Dombrock blood group) | -2.5 |
| KCNJ8 | potassium inwardly-rectifying channel, subfamily J, member 8 | -2.5 |
| PHGDH | phosphoglycerate dehydrogenase | -2.5 |
| SARDH | sarcosine dehydrogenase | -2.5 |
| PRRG4 | proline rich Gla (G-carboxyglutamic acid) 4 (transmembrane) | -2.5 |
| TEK | TEK tyrosine kinase, endothelial | -2.5 |
| SORL1 | sortilin-related receptor, L(DLR class) A repeats containing | -2.5 |
| CR1 | complement component (3b/4b) receptor 1 (Knops blood group) | -2.5 |
| C1R | complement component 1, r subcomponent | -2.5 |
| IGFALS | insulin-like growth factor binding protein, acid labile subunit | -2.5 |
| RIPK4 | receptor-interacting serine-threonine kinase 4 | -2.5 |
| CCL4 | chemokine (C-C motif) ligand 4 | -2.5 |
| PLIN1 | perilipin 1 | -2.5 |
| MRGPRF | MAS-related GPR, member F | -2.5 |
| FNIP2 | folliculin interacting protein 2 | -2.5 |
| ATOH8 | atonal homolog 8 (Drosophila) | -2.5 |
| BCKDHB | branched chain keto acid dehydrogenase E1, beta polypeptide | -2.5 |
| ZNF295 | zinc finger protein 295 | -2.5 |
| GNPNAT1 | glucosamine-phosphate N-acetyltransferase 1 | -2.5 |
| KLRF1 | killer cell lectin-like receptor subfamily F, member 1 | -2.5 |
| SPRYD4 | SPRY domain containing 4 | -2.5 |
| IDNK | idnK, gluconokinase homolog (E. coli) | -2.5 |
| TMPRSS2 | transmembrane protease, serine 2 | -2.5 |
| GPR125 | G protein-coupled receptor 125 | -2.5 |
| LPIN2 | lipin 2 | -2.5 |
| IGLL1/IGLL5 | immunoglobulin lambda-like polypeptide 1 | -2.5 |
| SPATA6L | spermatogenesis associated 6-like | -2.6 |
| ACACB | acetyl-CoA carboxylase beta | -2.6 |
| DUSP10 | dual specificity phosphatase 10 | -2.6 |
| FRMD6 | FERM domain containing 6 | -2.6 |
| FERMT2 | fermitin family member 2 | -2.6 |
| PTH1R | parathyroid hormone 1 receptor | -2.6 |
| GJB2 | gap junction protein, beta 2, 26kDa | -2.6 |
| MCTP2 | multiple C2 domains, transmembrane 2 | -2.6 |
| GADD45A | growth arrest and DNA-damage-inducible, alpha | -2.6 |
| SLC19A3 | solute carrier family 19, member 3 | -2.6 |
| ANKRD36BP2 | ankyrin repeat domain 36B pseudogene 2 | -2.6 |
| GIPC2 | GIPC PDZ domain containing family, member 2 | -2.6 |
| SLC25A20 | solute carrier family 25 (carnitine/acylcarnitine translocase), member 20 | -2.6 |
| MMRN1 | multimerin 1 | -2.6 |
| PSD3 | pleckstrin and Sec7 domain containing 3 | -2.6 |
| ACOX1 | acyl-CoA oxidase 1, palmitoyl | -2.6 |
| C6orf123 | chromosome 6 open reading frame 123 | -2.6 |
| MST1 | macrophage stimulating 1 (hepatocyte growth factor-like) | -2.6 |
| GCGR | glucagon receptor | -2.6 |
| RGS4 | regulator of G-protein signaling 4 | -2.6 |
| PAIP2B | poly(A) binding protein interacting protein 2B | -2.6 |
| KCNMA1 | potassium large conductance calcium-activated channel, subfamily M, alpha member 1 | -2.6 |
| NDRG2 | NDRG family member 2 | -2.6 |
| DAK | dihydroxyacetone kinase 2 homolog (S. cerevisiae) | -2.6 |
| VIPR1 | vasoactive intestinal peptide receptor 1 | -2.6 |
| GRAMD4 | GRAM domain containing 4 | -2.6 |
| CYP2J2 | cytochrome P450, family 2, subfamily J, polypeptide 2 | -2.6 |
| CBR4 | carbonyl reductase 4 | -2.6 |
| GFRA1 | GDNF family receptor alpha 1 | -2.6 |
| LARP1B | La ribonucleoprotein domain family, member 1B | -2.6 |
| BHLHE40 | basic helix-loop-helix family, member e40 | -2.6 |
| ACAA1 | acetyl-CoA acyltransferase 1 | -2.6 |
| CNTN4 | contactin 4 | -2.6 |
| KLHL15 | kelch-like 15 (Drosophila) | -2.6 |
| SCARA5 | scavenger receptor class A, member 5 (putative) | -2.7 |
| KRTCAP3 | keratinocyte associated protein 3 | -2.7 |
| DLC1 | deleted in liver cancer 1 | -2.7 |
| DBH | dopamine beta-hydroxylase (dopamine beta-monooxygenase) | -2.7 |
| SLC2A9 | solute carrier family 2 (facilitated glucose transporter), member 9 | -2.7 |
| GPD1 | glycerol-3-phosphate dehydrogenase 1 (soluble) | -2.7 |
| LOC200772 | uncharacterized LOC200772 | -2.7 |
| SORBS1 | sorbin and SH3 domain containing 1 | -2.7 |
| EPHA2 | EPH receptor A2 | -2.7 |
| ANKRD55 | ankyrin repeat domain 55 | -2.7 |
| NTS | neurotensin | -2.7 |
| PHYH | phytanoyl-CoA 2-hydroxylase | -2.7 |
| CCL14 | chemokine (C-C motif) ligand 14 | -2.7 |
| VTN | vitronectin | -2.7 |
| RAB25 | RAB25, member RAS oncogene family | -2.7 |
| BTG2 | BTG family, member 2 | -2.7 |
| GK | glycerol kinase | -2.7 |
| SHBG | sex hormone-binding globulin | -2.7 |
| RAB27A | RAB27A, member RAS oncogene family | -2.7 |
| DHRS1 | dehydrogenase/reductase (SDR family) member 1 | -2.7 |
| TCF21 | transcription factor 21 | -2.7 |
| ESRP2 | epithelial splicing regulatory protein 2 | -2.7 |
| TJP2 | tight junction protein 2 | -2.7 |
| GCDH | glutaryl-CoA dehydrogenase | -2.7 |
| LIPC | lipase, hepatic | -2.7 |
| HAS2 | hyaluronan synthase 2 | -2.7 |
| SERPINA6 | serpin peptidase inhibitor, clade A (alpha-1 antiproteinase, antitrypsin), member 6 | -2.7 |
| CD8A | CD8a molecule | -2.7 |
| PEMT | phosphatidylethanolamine N-methyltransferase | -2.7 |
| NBLA00301 | Nbla00301 | -2.7 |
| ACAA2 | acetyl-CoA acyltransferase 2 | -2.7 |
| MYOM2 | myomesin (M-protein) 2, 165kDa | -2.8 |
| PLIN2 | perilipin 2 | -2.8 |
| CCDC3 | coiled-coil domain containing 3 | -2.8 |
| SMAD9 | SMAD family member 9 | -2.8 |
| THBD | thrombomodulin | -2.8 |
| SLC9B2 | solute carrier family 9, subfamily B (NHA2, cation proton antiporter 2), member 2 | -2.8 |
| PRR18 | proline rich 18 | -2.8 |
| SCD5 | stearoyl-CoA desaturase 5 | -2.8 |
| DACH1 | dachshund homolog 1 (Drosophila) | -2.8 |
| EPB41L5 | erythrocyte membrane protein band 4.1 like 5 | -2.8 |
| WWC1 | WW and C2 domain containing 1 | -2.8 |
| EIF5 | eukaryotic translation initiation factor 5 | -2.8 |
| RND1 | Rho family GTPase 1 | -2.8 |
| IL33 | interleukin 33 | -2.8 |
| CCDC71L | coiled-coil domain containing 71-like | -2.8 |
| FMO2 | flavin containing monooxygenase 2 (non-functional) | -2.8 |
| SEMA6D | sema domain, transmembrane domain (TM), and cytoplasmic domain, (semaphorin) 6D | -2.8 |
| GPT2 | glutamic pyruvate transaminase (alanine aminotransferase) 2 | -2.8 |
| JUNB | jun B proto-oncogene | -2.9 |
| COL6A6 | collagen, type VI, alpha 6 | -2.9 |
| GADD45G | growth arrest and DNA-damage-inducible, gamma | -2.9 |
| ARHGEF26 | Rho guanine nucleotide exchange factor (GEF) 26 | -2.9 |
| PPP2R1B | protein phosphatase 2, regulatory subunit A, beta | -2.9 |
| IGLV3-21 | immunoglobulin lambda variable 3-21 | -2.9 |
| SLC20A1 | solute carrier family 20 (phosphate transporter), member 1 | -2.9 |
| MST1P9 | macrophage stimulating 1 (hepatocyte growth factor-like) pseudogene 9 | -2.9 |
| AGPAT9 | 1-acylglycerol-3-phosphate O-acyltransferase 9 | -2.9 |
| PFKFB3 | 6-phosphofructo-2-kinase/fructose-2,6-biphosphatase 3 | -2.9 |
| DLL1 | delta-like 1 (Drosophila) | -2.9 |
| CPED1 | cadherin-like and PC-esterase domain containing 1 | -2.9 |
| FAM59A | family with sequence similarity 59, member A | -2.9 |
| EHD3 | EH-domain containing 3 | -2.9 |
| SGK1 | serum/glucocorticoid regulated kinase 1 | -2.9 |
| FAM149A | family with sequence similarity 149, member A | -2.9 |
| ANGPTL6 | angiopoietin-like 6 | -2.9 |
| MPC1 | mitochondrial pyruvate carrier 1 | -2.9 |
| NR4A3 | nuclear receptor subfamily 4, group A, member 3 | -2.9 |
| GPR126 | G protein-coupled receptor 126 | -2.9 |
| AADAT | aminoadipate aminotransferase | -2.9 |
| ASL | argininosuccinate lyase | -2.9 |
| LOC100129447 | uncharacterized LOC100129447 | -2.9 |
| GSTZ1 | glutathione S-transferase zeta 1 | -2.9 |
| PLCXD3 | phosphatidylinositol-specific phospholipase C, X domain containing 3 | -2.9 |
| CD274 | CD274 molecule | -2.9 |
| TSLP | thymic stromal lymphopoietin | -2.9 |
| KAZN | kazrin, periplakin interacting protein | -2.9 |
| TGFA | transforming growth factor, alpha | -2.9 |
| PTN | pleiotrophin | -3.0 |
| SC5DL | sterol-C5-desaturase (ERG3 delta-5-desaturase homolog, S. cerevisiae)-like | -3.0 |
| CYFIP2 | cytoplasmic FMR1 interacting protein 2 | -3.0 |
| MIR22HG | MIR22 host gene (non-protein coding) | -3.0 |
| CHRDL1 | chordin-like 1 | -3.0 |
| IGFBP1 | insulin-like growth factor binding protein 1 | -3.0 |
| DUSP2 | dual specificity phosphatase 2 | -3.0 |
| ITIH4 | inter-alpha-trypsin inhibitor heavy chain family, member 4 | -3.0 |
| HAO1 | hydroxyacid oxidase (glycolate oxidase) 1 | -3.0 |
| F13B | coagulation factor XIII, B polypeptide | -3.0 |
| SLC16A2 | solute carrier family 16, member 2 (thyroid hormone transporter) | -3.0 |
| NAPSB | napsin B aspartic peptidase, pseudogene | -3.0 |
| PZP | pregnancy-zone protein | -3.0 |
| HOGA1 | 4-hydroxy-2-oxoglutarate aldolase 1 | -3.0 |
| MUT | methylmalonyl CoA mutase | -3.0 |
| PHLDA1 | pleckstrin homology-like domain, family A, member 1 | -3.0 |
| GATA6 | GATA binding protein 6 | -3.1 |
| KLF6 | Kruppel-like factor 6 | -3.1 |
| PDE7B | phosphodiesterase 7B | -3.1 |
| BDH2 | 3-hydroxybutyrate dehydrogenase, type 2 | -3.1 |
| TCTEX1D1 | Tctex1 domain containing 1 | -3.1 |
| CP | ceruloplasmin (ferroxidase) | -3.1 |
| PCSK6 | proprotein convertase subtilisin/kexin type 6 | -3.1 |
| CDC37L1 | cell division cycle 37 homolog (S. cerevisiae)-like 1 | -3.1 |
| RGN | regucalcin (senescence marker protein-30) | -3.1 |
| DNM3OS | DNM3 opposite strand/antisense RNA | -3.1 |
| DPF3 | D4, zinc and double PHD fingers, family 3 | -3.1 |
| FXYD1 | FXYD domain containing ion transport regulator 1 | -3.1 |
| RNF165 | ring finger protein 165 | -3.1 |
| IL18R1 | interleukin 18 receptor 1 | -3.1 |
| LY6E | lymphocyte antigen 6 complex, locus E | -3.1 |
| LOC255167 | uncharacterized LOC255167 | -3.1 |
| APBA1 | amyloid beta (A4) precursor protein-binding, family A, member 1 | -3.1 |
| SERPINB9 | serpin peptidase inhibitor, clade B (ovalbumin), member 9 | -3.2 |
| FLJ22763 | uncharacterized LOC401081 | -3.2 |
| F11 | coagulation factor XI | -3.2 |
| MAN1C1 | mannosidase, alpha, class 1C, member 1 | -3.2 |
| TARP | TCR gamma alternate reading frame protein | -3.2 |
| TMEM56 | transmembrane protein 56 | -3.2 |
| C8A | complement component 8, alpha polypeptide | -3.2 |
| MS4A1 | membrane-spanning 4-domains, subfamily A, member 1 | -3.2 |
| FXYD2 | FXYD domain containing ion transport regulator 2 | -3.2 |
| IGLL3P | immunoglobulin lambda-like polypeptide 3, pseudogene | -3.2 |
| GZMK | granzyme K (granzyme 3; tryptase II) | -3.2 |
| SRD5A1 | steroid-5-alpha-reductase, alpha polypeptide 1 (3-oxo-5 alpha-steroid delta 4-dehydrogenase alpha 1) | -3.2 |
| PAMR1 | peptidase domain containing associated with muscle regeneration 1 | -3.2 |
| PDLIM5 | PDZ and LIM domain 5 | -3.2 |
| ALDH8A1 | aldehyde dehydrogenase 8 family, member A1 | -3.2 |
| CD1D | CD1d molecule | -3.2 |
| MAT1A | methionine adenosyltransferase I, alpha | -3.2 |
| FEZ1 | fasciculation and elongation protein zeta 1 (zygin I) | -3.3 |
| SLCO1B1 | solute carrier organic anion transporter family, member 1B1 | -3.3 |
| SNORA28 | small nucleolar RNA, H/ACA box 28 | -3.3 |
| EPB41L4A | erythrocyte membrane protein band 4.1 like 4A | -3.3 |
| ADAMTSL3 | ADAMTS-like 3 | -3.3 |
| ACSM3 | acyl-CoA synthetase medium-chain family member 3 | -3.3 |
| C5orf4 | chromosome 5 open reading frame 4 | -3.3 |
| LOC286087 | uncharacterized LOC286087 | -3.3 |
| DEPDC7 | DEP domain containing 7 | -3.3 |
| KCNJ16 | potassium inwardly-rectifying channel, subfamily J, member 16 | -3.3 |
| ZGPAT | zinc finger, CCCH-type with G patch domain | -3.3 |
| ETFDH | electron-transferring-flavoprotein dehydrogenase | -3.3 |
| AGTR1 | angiotensin II receptor, type 1 | -3.3 |
| SAA2-SAA4/SAA4 | serum amyloid A4, constitutive | -3.3 |
| SLC41A2 | solute carrier family 41, member 2 | -3.4 |
| RCL1 | RNA terminal phosphate cyclase-like 1 | -3.4 |
| BCHE | butyrylcholinesterase | -3.4 |
| ZFP36 | zinc finger protein 36, C3H type, homolog (mouse) | -3.4 |
| NAAA | N-acylethanolamine acid amidase | -3.4 |
| MYO10 | myosin X | -3.4 |
| ERRFI1 | ERBB receptor feedback inhibitor 1 | -3.4 |
| PCDH9 | protocadherin 9 | -3.4 |
| CYP2C19 | cytochrome P450, family 2, subfamily C, polypeptide 19 | -3.4 |
| CYR61 | cysteine-rich, angiogenic inducer, 61 | -3.4 |
| PPAP2B | phosphatidic acid phosphatase type 2B | -3.4 |
| PPP1R1A | protein phosphatase 1, regulatory (inhibitor) subunit 1A | -3.4 |
| ACADSB | acyl-CoA dehydrogenase, short/branched chain | -3.4 |
| A2M | alpha-2-macroglobulin | -3.4 |
| FOXO1 | forkhead box O1 | -3.4 |
| EDNRB | endothelin receptor type B | -3.4 |
| ANGPTL4 | angiopoietin-like 4 | -3.4 |
| GPR171 | G protein-coupled receptor 171 | -3.4 |
| SLC22A7 | solute carrier family 22 (organic anion transporter), member 7 | -3.4 |
| KBTBD11 | kelch repeat and BTB (POZ) domain containing 11 | -3.4 |
| JUN | jun proto-oncogene | -3.4 |
| NFIL3 | nuclear factor, interleukin 3 regulated | -3.5 |
| OGDHL | oxoglutarate dehydrogenase-like | -3.5 |
| MBNL2 | muscleblind-like splicing regulator 2 | -3.5 |
| TUBE1 | tubulin, epsilon 1 | -3.5 |
| GLDC | glycine dehydrogenase (decarboxylating) | -3.5 |
| SLCO4C1 | solute carrier organic anion transporter family, member 4C1 | -3.5 |
| ADCY1 | adenylate cyclase 1 (brain) | -3.5 |
| PANK1 | pantothenate kinase 1 | -3.5 |
| CYP2A7 | cytochrome P450, family 2, subfamily A, polypeptide 7 | -3.5 |
| EGR2 | early growth response 2 | -3.5 |
| FGB | fibrinogen beta chain | -3.5 |
| SPATA18 | spermatogenesis associated 18 | -3.5 |
| ST6GAL2 | ST6 beta-galactosamide alpha-2,6-sialyltranferase 2 | -3.5 |
| TNFRSF10D | tumor necrosis factor receptor superfamily, member 10d, decoy with truncated death domain | -3.6 |
| BACH2 | BTB and CNC homology 1, basic leucine zipper transcription factor 2 | -3.6 |
| ADAMTS13 | ADAM metallopeptidase with thrombospondin type 1 motif, 13 | -3.6 |
| FAM13A | family with sequence similarity 13, member A | -3.6 |
| AMDHD1 | amidohydrolase domain containing 1 | -3.6 |
| MFAP3L | microfibrillar-associated protein 3-like | -3.6 |
| EPHX2 | epoxide hydrolase 2, cytoplasmic | -3.6 |
| PROZ | protein Z, vitamin K-dependent plasma glycoprotein | -3.6 |
| CFP | complement factor properdin | -3.6 |
| ITGA9 | integrin, alpha 9 | -3.7 |
| KDM8 | lysine (K)-specific demethylase 8 | -3.7 |
| IL1RL1 | interleukin 1 receptor-like 1 | -3.7 |
| CH25H | cholesterol 25-hydroxylase | -3.7 |
| ESRP1 | epithelial splicing regulatory protein 1 | -3.7 |
| ANGPTL3 | angiopoietin-like 3 | -3.7 |
| ACMSD | aminocarboxymuconate semialdehyde decarboxylase | -3.7 |
| CNGA1 | cyclic nucleotide gated channel alpha 1 | -3.7 |
| SYNPO2 | synaptopodin 2 | -3.7 |
| RNF125 | ring finger protein 125, E3 ubiquitin protein ligase | -3.7 |
| LDLR | low density lipoprotein receptor | -3.7 |
| CDH1 | cadherin 1, type 1, E-cadherin (epithelial) | -3.7 |
| ID2 | inhibitor of DNA binding 2, dominant negative helix-loop-helix protein | -3.7 |
| POU2AF1 | POU class 2 associating factor 1 | -3.7 |
| PDK4 | pyruvate dehydrogenase kinase, isozyme 4 | -3.7 |
| CDH19 | cadherin 19, type 2 | -3.7 |
| SLC25A18 | solute carrier family 25 (glutamate carrier), member 18 | -3.8 |
| CD14 | CD14 molecule | -3.8 |
| PITPNM3 | PITPNM family member 3 | -3.8 |
| GOT1 | glutamic-oxaloacetic transaminase 1, soluble (aspartate aminotransferase 1) | -3.8 |
| ZNF498 | zinc finger protein 498 | -3.8 |
| CSRNP1 | cysteine-serine-rich nuclear protein 1 | -3.8 |
| CYP3A5 | cytochrome P450, family 3, subfamily A, polypeptide 5 | -3.8 |
| IGFBP3 | insulin-like growth factor binding protein 3 | -3.8 |
| SLC25A15 | solute carrier family 25 (mitochondrial carrier; ornithine transporter) member 15 | -3.8 |
| ANGPTL1 | angiopoietin-like 1 | -3.8 |
| SOCS3 | suppressor of cytokine signaling 3 | -3.8 |
| ECM1 | extracellular matrix protein 1 | -3.8 |
| MCC | mutated in colorectal cancers | -3.8 |
| CYP4F3 | cytochrome P450, family 4, subfamily F, polypeptide 3 | -3.9 |
| INMT | indolethylamine N-methyltransferase | -3.9 |
| MRC1 | mannose receptor, C type 1 | -3.9 |
| HBA1/HBA2 | hemoglobin, alpha 1 | -3.9 |
| ENO3 | enolase 3 (beta, muscle) | -3.9 |
| CTH | cystathionase (cystathionine gamma-lyase) | -3.9 |
| CHST9 | carbohydrate (N-acetylgalactosamine 4-0) sulfotransferase 9 | -3.9 |
| DUSP5 | dual specificity phosphatase 5 | -3.9 |
| EPB41L4B | erythrocyte membrane protein band 4.1 like 4B | -3.9 |
| CDHR2 | cadherin-related family member 2 | -3.9 |
| NNMT | nicotinamide N-methyltransferase | -3.9 |
| KIAA0146 | KIAA0146 | -3.9 |
| DMGDH | dimethylglycine dehydrogenase | -3.9 |
| PALM2 | paralemmin 2 | -4.0 |
| PON3 | paraoxonase 3 | -4.0 |
| C1orf168 | chromosome 1 open reading frame 168 | -4.0 |
| SERPINA5 | serpin peptidase inhibitor, clade A (alpha-1 antiproteinase, antitrypsin), member 5 | -4.0 |
| ZFPM2 | zinc finger protein, multitype 2 | -4.0 |
| FAM150B | family with sequence similarity 150, member B | -4.0 |
| RBMS3 | RNA binding motif, single stranded interacting protein 3 | -4.0 |
| HRSP12 | heat-responsive protein 12 | -4.0 |
| ABI3BP | ABI family, member 3 (NESH) binding protein | -4.0 |
| TIMD4 | T-cell immunoglobulin and mucin domain containing 4 | -4.0 |
| CEBPD | CCAAT/enhancer binding protein (C/EBP), delta | -4.0 |
| CD69 | CD69 molecule | -4.0 |
| ETS2 | v-ets erythroblastosis virus E26 oncogene homolog 2 (avian) | -4.1 |
| CRISPLD2 | cysteine-rich secretory protein LCCL domain containing 2 | -4.1 |
| DUSP1 | dual specificity phosphatase 1 | -4.1 |
| NAT8 | N-acetyltransferase 8 (GCN5-related, putative) | -4.1 |
| ID1 | inhibitor of DNA binding 1, dominant negative helix-loop-helix protein | -4.1 |
| NAMPT | nicotinamide phosphoribosyltransferase | -4.1 |
| GPLD1 | glycosylphosphatidylinositol specific phospholipase D1 | -4.2 |
| SLC38A4 | solute carrier family 38, member 4 | -4.2 |
| PPID | peptidylprolyl isomerase D | -4.2 |
| ANO1 | anoctamin 1, calcium activated chloride channel | -4.2 |
| FOLH1 | folate hydrolase (prostate-specific membrane antigen) 1 | -4.2 |
| GPR182 | G protein-coupled receptor 182 | -4.2 |
| C3P1 | complement component 3 precursor pseudogene | -4.2 |
| CLDN10 | claudin 10 | -4.2 |
| CPN1 | carboxypeptidase N, polypeptide 1 | -4.2 |
| AQP3 | aquaporin 3 (Gill blood group) | -4.2 |
| GREM2 | gremlin 2 | -4.2 |
| KLKB1 | kallikrein B, plasma (Fletcher factor) 1 | -4.2 |
| RND3 | Rho family GTPase 3 | -4.2 |
| AGXT2L1 | alanine-glyoxylate aminotransferase 2-like 1 | -4.2 |
| LYVE1 | lymphatic vessel endothelial hyaluronan receptor 1 | -4.3 |
| AKAP12 | A kinase (PRKA) anchor protein 12 | -4.3 |
| STAB2 | stabilin 2 | -4.3 |
| ACADL | acyl-CoA dehydrogenase, long chain | -4.3 |
| CYP4V2 | cytochrome P450, family 4, subfamily V, polypeptide 2 | -4.3 |
| FNDC5 | fibronectin type III domain containing 5 | -4.3 |
| GCH1 | GTP cyclohydrolase 1 | -4.3 |
| RCAN1 | regulator of calcineurin 1 | -4.4 |
| FOLH1B | folate hydrolase 1B | -4.4 |
| TBX15 | T-box 15 | -4.4 |
| MASP2 | mannan-binding lectin serine peptidase 2 | -4.4 |
| LIPG | lipase, endothelial | -4.4 |
| MRO | maestro | -4.4 |
| CPEB3 | cytoplasmic polyadenylation element binding protein 3 | -4.4 |
| ADAMTS1 | ADAM metallopeptidase with thrombospondin type 1 motif, 1 | -4.4 |
| MOGAT2 | monoacylglycerol O-acyltransferase 2 | -4.5 |
| N4BP2L1 | NEDD4 binding protein 2-like 1 | -4.5 |
| ATF3 | activating transcription factor 3 | -4.5 |
| TMEM30B | transmembrane protein 30B | -4.5 |
| PPARGC-1α/PGC-1α | peroxisome proliferator-activated receptor gamma, coactivator 1 alpha | -4.5 |
| SLC27A2 | solute carrier family 27 (fatty acid transporter), member 2 | -4.5 |
| CLIC6 | chloride intracellular channel 6 | -4.5 |
| CHST4 | carbohydrate (N-acetylglucosamine 6-O) sulfotransferase 4 | -4.6 |
| OLFML3 | olfactomedin-like 3 | -4.6 |
| OAT | ornithine aminotransferase | -4.6 |
| HBB | hemoglobin, beta | -4.6 |
| ATF5 | activating transcription factor 5 | -4.6 |
| CFTR | cystic fibrosis transmembrane conductance regulator (ATP-binding cassette sub-family C, member 7) | -4.6 |
| TFPI2 | tissue factor pathway inhibitor 2 | -4.6 |
| AZGP1 | alpha-2-glycoprotein 1, zinc-binding | -4.6 |
| CYP4F2 | cytochrome P450, family 4, subfamily F, polypeptide 2 | -4.6 |
| FAM134B | family with sequence similarity 134, member B | -4.6 |
| C11orf96 | chromosome 11 open reading frame 96 | -4.6 |
| MT2A | metallothionein 2A | -4.7 |
| GABRP | gamma-aminobutyric acid (GABA) A receptor, pi | -4.7 |
| MASP1 | mannan-binding lectin serine peptidase 1 (C4/C2 activating component of Ra-reactive factor) | -4.7 |
| ALDH6A1 | aldehyde dehydrogenase 6 family, member A1 | -4.8 |
| ANK3 | ankyrin 3, node of Ranvier (ankyrin G) | -4.8 |
| NPY1R | neuropeptide Y receptor Y1 | -4.8 |
| VNN1 | vanin 1 | -4.9 |
| ID4 | inhibitor of DNA binding 4, dominant negative helix-loop-helix protein | -4.9 |
| CIDEB | cell death-inducing DFFA-like effector b | -4.9 |
| FTCD | formiminotransferase cyclodeaminase | -4.9 |
| EGR1 | early growth response 1 | -5.0 |
| SLC7A2 | solute carrier family 7 (cationic amino acid transporter, y+ system), member 2 | -5.0 |
| TAT | tyrosine aminotransferase | -5.0 |
| CCBE1 | collagen and calcium binding EGF domains 1 | -5.0 |
| BBOX1 | butyrobetaine (gamma), 2-oxoglutarate dioxygenase (gamma-butyrobetaine hydroxylase) 1 | -5.0 |
| TMEM27 | transmembrane protein 27 | -5.0 |
| C8B | complement component 8, beta polypeptide | -5.0 |
| C8orf4 | chromosome 8 open reading frame 4 | -5.0 |
| SRD5A2 | steroid-5-alpha-reductase, alpha polypeptide 2 (3-oxo-5 alpha-steroid delta 4-dehydrogenase alpha 2) | -5.0 |
| SIK1 | salt-inducible kinase 1 | -5.1 |
| ABCA8 | ATP-binding cassette, sub-family A (ABC1), member 8 | -5.1 |
| ITGB8 | integrin, beta 8 | -5.1 |
| HMGCS2 | 3-hydroxy-3-methylglutaryl-CoA synthase 2 (mitochondrial) | -5.2 |
| COLEC11 | collectin sub-family member 11 | -5.2 |
| SH3YL1 | SH3 domain containing, Ysc84-like 1 (S. cerevisiae) | -5.2 |
| HRG | histidine-rich glycoprotein | -5.3 |
| SERPINA4 | serpin peptidase inhibitor, clade A (alpha-1 antiproteinase, antitrypsin), member 4 | -5.3 |
| NAT2 | N-acetyltransferase 2 (arylamine N-acetyltransferase) | -5.3 |
| CYP2C18 | cytochrome P450, family 2, subfamily C, polypeptide 18 | -5.4 |
| FETUB | fetuin B | -5.4 |
| CNTN3 | contactin 3 (plasmacytoma associated) | -5.4 |
| STEAP4 | STEAP family member 4 | -5.4 |
| MT1H | metallothionein 1H | -5.4 |
| BCO2 | beta-carotene oxygenase 2 | -5.4 |
| HGFAC | HGF activator | -5.5 |
| MT1E | metallothionein 1E | -5.5 |
| COLEC10 | collectin sub-family member 10 (C-type lectin) | -5.6 |
| BHMT | betaine--homocysteine S-methyltransferase | -5.6 |
| MT1X | metallothionein 1X | -5.6 |
| MT1G | metallothionein 1G | -5.6 |
| FAM110C | family with sequence similarity 110, member C | -5.6 |
| ASS1 | argininosuccinate synthase 1 | -5.7 |
| A1BG | alpha-1-B glycoprotein | -5.8 |
| HPGD | hydroxyprostaglandin dehydrogenase 15-(NAD) | -5.8 |
| AVPR1A | arginine vasopressin receptor 1A | -5.8 |
| CXCL2 | chemokine (C-X-C motif) ligand 2 | -5.8 |
| ANXA10 | annexin A10 | -5.8 |
| HPX | hemopexin | -5.9 |
| MBL2 | mannose-binding lectin (protein C) 2, soluble | -5.9 |
| ACSL1 | acyl-CoA synthetase long-chain family member 1 | -5.9 |
| LCAT | lecithin-cholesterol acyltransferase | -6.0 |
| RSPO3 | R-spondin 3 | -6.0 |
| NR4A2 | nuclear receptor subfamily 4, group A, member 2 | -6.0 |
| ZG16 | zymogen granule protein 16 homolog (rat) | -6.0 |
| XDH | xanthine dehydrogenase | -6.1 |
| PLGLB1/PLGLB2 | plasminogen-like B2 | -6.1 |
| PRG4 | proteoglycan 4 | -6.1 |
| APOA5 | apolipoprotein A-V | -6.1 |
| DPT | dermatopontin | -6.2 |
| FAM65C | family with sequence similarity 65, member C | -6.2 |
| IL13RA2 | interleukin 13 receptor, alpha 2 | -6.3 |
| AGXT | alanine-glyoxylate aminotransferase | -6.4 |
| LEPR | leptin receptor | -6.5 |
| SRPX | sushi-repeat containing protein, X-linked | -6.5 |
| GLYAT | glycine-N-acyltransferase | -6.6 |
| CYP3A43 | cytochrome P450, family 3, subfamily A, polypeptide 43 | -6.7 |
| IGKC | immunoglobulin kappa constant | -6.7 |
| NRG1 | neuregulin 1 | -6.8 |
| TACSTD2 | tumor-associated calcium signal transducer 2 | -6.8 |
| HGF | hepatocyte growth factor (hepapoietin A; scatter factor) | -6.8 |
| RDH16 | retinol dehydrogenase 16 (all-trans) | -6.9 |
| HSD17B2 | hydroxysteroid (17-beta) dehydrogenase 2 | -6.9 |
| MT1F | metallothionein 1F | -6.9 |
| CD5L | CD5 molecule-like | -7.0 |
| IDO2 | indoleamine 2,3-dioxygenase 2 | -7.0 |
| GLYATL1 | glycine-N-acyltransferase-like 1 | -7.1 |
| GBA3 | glucosidase, beta, acid 3 (cytosolic) | -7.1 |
| CA2 | carbonic anhydrase II | -7.2 |
| C6 | complement component 6 | -7.2 |
| PLAC8 | placenta-specific 8 | -7.2 |
| G6PC | glucose-6-phosphatase, catalytic subunit | -7.2 |
| PTGS2 | prostaglandin-endoperoxide synthase 2 (prostaglandin G/H synthase and cyclooxygenase) | -7.3 |
| FGFR2 | fibroblast growth factor receptor 2 | -7.3 |
| ASPA | aspartoacylase | -7.3 |
| TTC36 | tetratricopeptide repeat domain 36 | -7.4 |
| IGL@ | immunoglobulin lambda locus | -7.4 |
| KCNN2 | potassium intermediate/small conductance calcium-activated channel, subfamily N, member 2 | -7.4 |
| KMO | kynurenine 3-monooxygenase (kynurenine 3-hydroxylase) | -7.4 |
| TDO2 | tryptophan 2,3-dioxygenase | -7.5 |
| GRAMD1C | GRAM domain containing 1C | -7.6 |
| CYP3A4 | cytochrome P450, family 3, subfamily A, polypeptide 4 | -7.6 |
| FABP1 | fatty acid binding protein 1, liver | -7.6 |
| IGHM | immunoglobulin heavy constant mu | -7.7 |
| GPM6A | glycoprotein M6A | -7.7 |
| SLC10A1 | solute carrier family 10 (sodium/bile acid cotransporter family), member 1 | -7.7 |
| SLC13A5 | solute carrier family 13 (sodium-dependent citrate transporter), member 5 | -7.7 |
| CYP2A13/CYP2A6 | cytochrome P450, family 2, subfamily A, polypeptide 6 | -7.8 |
| LIFR | leukemia inhibitory factor receptor alpha | -7.9 |
| CLEC4M | C-type lectin domain family 4, member M | -7.9 |
| UNC93A | unc-93 homolog A (C. elegans) | -8.0 |
| CETP | cholesteryl ester transfer protein, plasma | -8.1 |
| LPA | lipoprotein, Lp(a) | -8.2 |
| FOS | FBJ murine osteosarcoma viral oncogene homolog | -8.4 |
| GADD45B | growth arrest and DNA-damage-inducible, beta | -8.4 |
| CYP26A1 | cytochrome P450, family 26, subfamily A, polypeptide 1 | -8.6 |
| THBS1 | thrombospondin 1 | -8.8 |
| CYP2C9 | cytochrome P450, family 2, subfamily C, polypeptide 9 | -8.8 |
| ODZ1 | odz, odd Oz/ten-m homolog 1 (Drosophila) | -8.9 |
| EPCAM | epithelial cell adhesion molecule | -8.9 |
| CYP4A22 | cytochrome P450, family 4, subfamily A, polypeptide 22 | -8.9 |
| SLC51A | solute carrier family 51, alpha subunit | -9.1 |
| SOCS2 | suppressor of cytokine signaling 2 | -9.1 |
| ESR1 | estrogen receptor 1 | -9.4 |
| IGF1 | insulin-like growth factor 1 (somatomedin C) | -9.5 |
| SLC25A47 | solute carrier family 25, member 47 | -9.5 |
| SDS | serine dehydratase | -9.6 |
| ACOT12 | acyl-CoA thioesterase 12 | -9.6 |
| IL1RAP | interleukin 1 receptor accessory protein | -9.6 |
| CYP4A11 | cytochrome P450, family 4, subfamily A, polypeptide 11 | -9.6 |
| CYP2C8 | cytochrome P450, family 2, subfamily C, polypeptide 8 | -9.7 |
| GYS2 | glycogen synthase 2 (liver) | -9.9 |
| CNDP1 | carnosine dipeptidase 1 (metallopeptidase M20 family) | -9.9 |
| APOF | apolipoprotein F | -10.0 |
| IGHA1 | immunoglobulin heavy constant alpha 1 | -10.2 |
| CYP2B6 | cytochrome P450, family 2, subfamily B, polypeptide 6 | -10.3 |
| CYP3A7 | cytochrome P450, family 3, subfamily A, polypeptide 7 | -10.4 |
| FREM2 | FRAS1 related extracellular matrix protein 2 | -10.4 |
| DNASE1L3 | deoxyribonuclease I-like 3 | -10.5 |
| AFM | afamin | -10.5 |
| GNMT | glycine N-methyltransferase | -10.6 |
| HHIP | hedgehog interacting protein | -10.8 |
| DCN | decorin | -11.0 |
| HAL | histidine ammonia-lyase | -11.0 |
| ADH1A | alcohol dehydrogenase 1A (class I), alpha polypeptide | -11.0 |
| GHR | growth hormone receptor | -11.3 |
| SLCO1B3 | solute carrier organic anion transporter family, member 1B3 | -11.7 |
| MFSD2A | major facilitator superfamily domain containing 2A | -11.7 |
| PGLYRP2 | peptidoglycan recognition protein 2 | -11.8 |
| IGJ | immunoglobulin J polypeptide, linker protein for immunoglobulin alpha and mu polypeptides | -11.8 |
| CYP39A1 | cytochrome P450, family 39, subfamily A, polypeptide 1 | -11.8 |
| FOSB | FBJ murine osteosarcoma viral oncogene homolog B | -11.8 |
| FBP1 | fructose-1,6-bisphosphatase 1 | -12.0 |
| HAO2 | hydroxyacid oxidase 2 (long chain) | -12.0 |
| THRSP | thyroid hormone responsive | -12.3 |
| MT1M | metallothionein 1M | -12.6 |
| CLEC4G | C-type lectin domain family 4, member G | -12.9 |
| CXCL12 | chemokine (C-X-C motif) ligand 12 | -13.4 |
| CRHBP | corticotropin releasing hormone binding protein | -13.5 |
| ALDOB | aldolase B, fructose-bisphosphate | -13.6 |
| IGHG1 | immunoglobulin heavy constant gamma 1 (G1m marker) | -13.8 |
| MARCO | macrophage receptor with collagenous structure | -14.2 |
| CXCL14 | chemokine (C-X-C motif) ligand 14 | -14.4 |
| CYP1A2 | cytochrome P450, family 1, subfamily A, polypeptide 2 | -14.8 |
| GPR128 | G protein-coupled receptor 128 | -15.2 |
| ADH4 | alcohol dehydrogenase 4 (class II), pi polypeptide | -17.3 |
| C7 | complement component 7 | -17.5 |
| PCK1 | phosphoenolpyruvate carboxykinase 1 (soluble) | -17.7 |
| CLEC1B | C-type lectin domain family 1, member B | -17.8 |
| IGF2 | insulin-like growth factor 2 (somatomedin A) | -18.0 |
| AKR1D1 | aldo-keto reductase family 1, member D1 (delta 4-3-ketosteroid-5-beta-reductase) | -18.2 |
| GLS2 | glutaminase 2 (liver, mitochondrial) | -18.2 |
| FCN2 | ficolin (collagen/fibrinogen domain containing lectin) 2 (hucolin) | -20.7 |
| C9 | complement component 9 | -21.6 |
| FCN3 | ficolin (collagen/fibrinogen domain containing) 3 (Hakata antigen) | -21.9 |
| SPP2 | secreted phosphoprotein 2, 24kDa | -22.7 |
| SLC22A1 | solute carrier family 22 (organic cation transporter), member 1 | -22.8 |
| LOC100506229 | uncharacterized LOC100506229 | -24.8 |
| HAMP | hepcidin antimicrobial peptide | -27.3 |
| OIT3 | oncoprotein induced transcript 3 | -27.5 |
