# Supplementary material for: Viral expression and molecular profiling in liver tissue versus microdissected hepatocytes in hepatitis B virus - associated hepatocellular carcinoma
Source: J Transl Med. 2014 Aug 21;12:230. doi: 10.1186/s12967-014-0230-1 (PMC4142136; doi:10.1186/s12967-014-0230-1)
Supplement: Additional file 6: Table S5. — Differentially Expressed Genes Unique to Whole Liver Tissue. [file 12967_2014_230_MOESM6_ESM.docx]

| **Table S5.** Differentially Expressed Genes Unique to Whole Liver Tissue | | |
| --- | --- | --- |
| Gene Symbol | Gene Title | Fold Change |
| SFN | stratifin | 8.7 |
| CA12 | carbonic anhydrase XII | 5.0 |
| TTC39A | tetratricopeptide repeat domain 39A | 4.4 |
| TRIM55 | tripartite motif containing 55 | 4.2 |
| LOC100506403 | uncharacterized LOC100506403 | 4.0 |
| GPX2 | glutathione peroxidase 2 (gastrointestinal) | 3.7 |
| KIAA1244 | KIAA1244 | 3.7 |
| SP5 | Sp5 transcription factor | 3.0 |
| UHRF1 | ubiquitin-like with PHD and ring finger domains 1 | 2.9 |
| STEAP1 | six transmembrane epithelial antigen of the prostate 1 | 2.8 |
| MKI67 | antigen identified by monoclonal antibody Ki-67 | 2.8 |
| SDCBP2-AS1 | SDCBP2 antisense RNA 1 | 2.5 |
| EME1 | essential meiotic endonuclease 1 homolog 1 (S. pombe) | 2.5 |
| ZNF623 | zinc finger protein 623 | 2.5 |
| CDK5RAP2 | CDK5 regulatory subunit associated protein 2 | 2.4 |
| ABCB6 | ATP-binding cassette, sub-family B (MDR/TAP), member 6 | 2.3 |
| CCDC88A | coiled-coil domain containing 88A | 2.2 |
| SQLE | squalene epoxidase | 2.2 |
| PTGR1 | prostaglandin reductase 1 | 2.2 |
| THY1 | Thy-1 cell surface antigen | 2.2 |
| COL5A2 | collagen, type V, alpha 2 | 2.2 |
| ETV1 | ets variant 1 | 2.1 |
| PTP4A3 | protein tyrosine phosphatase type IVA, member 3 | 2.1 |
| PSRC1 | proline/serine-rich coiled-coil 1 | 2.1 |
| CENPJ | centromere protein J | 2.1 |
| COG2 | component of oligomeric golgi complex 2 | 2.1 |
| GPATCH2 | G patch domain containing 2 | 2.1 |
| PTK2 | PTK2 protein tyrosine kinase 2 | 2.1 |
| UGDH | UDP-glucose 6-dehydrogenase | 2.1 |
| SLC40A1 | solute carrier family 40 (iron-regulated transporter), member 1 | 2.0 |
| USP6NL | USP6 N-terminal like | -2.0 |
| C4orf19 | chromosome 4 open reading frame 19 | -2.0 |
| HAAO | 3-hydroxyanthranilate 3,4-dioxygenase | -2.0 |
| IPCEF1 | interaction protein for cytohesin exchange factors 1 | -2.0 |
| C1QTNF7 | C1q and tumor necrosis factor related protein 7 | -2.0 |
| ACADM | acyl-CoA dehydrogenase, C-4 to C-12 straight chain | -2.0 |
| BTD | biotinidase | -2.0 |
| TREH | trehalase (brush-border membrane glycoprotein) | -2.0 |
| TMEM86B | transmembrane protein 86B | -2.0 |
| NR3C2 | nuclear receptor subfamily 3, group C, member 2 | -2.0 |
| CNTLN | centlein, centrosomal protein | -2.0 |
| FDX1 | ferredoxin 1 | -2.0 |
| SELP | selectin P (granule membrane protein 140kDa, antigen CD62) | -2.0 |
| HDAC6 | histone deacetylase 6 | -2.0 |
| RAPGEF5 | Rap guanine nucleotide exchange factor (GEF) 5 | -2.0 |
| RAB17 | RAB17, member RAS oncogene family | -2.0 |
| KLF10 | Kruppel-like factor 10 | -2.0 |
| SLC37A4 | solute carrier family 37 (glucose-6-phosphate transporter), member 4 | -2.0 |
| PER1 | period homolog 1 (Drosophila) | -2.1 |
| SMOC1 | SPARC related modular calcium binding 1 | -2.1 |
| SYT9 | synaptotagmin IX | -2.1 |
| STOM | stomatin | -2.1 |
| LY9 | lymphocyte antigen 9 | -2.1 |
| FAM82A1 | family with sequence similarity 82, member A1 | -2.1 |
| SLC28A3 | solute carrier family 28 (sodium-coupled nucleoside transporter), member 3 | -2.1 |
| DCPS | decapping enzyme, scavenger | -2.1 |
| SHMT1 | serine hydroxymethyltransferase 1 (soluble) | -2.1 |
| ENDOG | endonuclease G | -2.1 |
| C14orf182 | chromosome 14 open reading frame 182 | -2.1 |
| GLIS3 | GLIS family zinc finger 3 | -2.1 |
| ATP5G3 | ATP synthase, H+ transporting, mitochondrial Fo complex, subunit C3 (subunit 9) | -2.1 |
| LURAP1L | leucine rich adaptor protein 1-like | -2.1 |
| SPRY2 | sprouty homolog 2 (Drosophila) | -2.1 |
| IGLJ3 | immunoglobulin lambda joining 3 | -2.1 |
| NR4A1 | nuclear receptor subfamily 4, group A, member 1 | -2.1 |
| EMR1 | egf-like module containing, mucin-like, hormone receptor-like 1 | -2.1 |
| TMEM220 | transmembrane protein 220 | -2.1 |
| MARC2 | mitochondrial amidoxime reducing component 2 | -2.1 |
| NR2F2-AS1 | NR2F2 antisense RNA 1 | -2.1 |
| F8 | coagulation factor VIII, procoagulant component | -2.1 |
| UAP1 | UDP-N-acteylglucosamine pyrophosphorylase 1 | -2.2 |
| GGT5 | gamma-glutamyltransferase 5 | -2.2 |
| PPAPDC1A | phosphatidic acid phosphatase type 2 domain containing 1A | -2.2 |
| ERLIN1 | ER lipid raft associated 1 | -2.2 |
| ATP11C | ATPase, class VI, type 11C | -2.2 |
| SATB1 | SATB homeobox 1 | -2.2 |
| KLRB1 | killer cell lectin-like receptor subfamily B, member 1 | -2.2 |
| ABHD15 | abhydrolase domain containing 15 | -2.2 |
| PCOLCE | procollagen C-endopeptidase enhancer | -2.2 |
| AVPI1 | arginine vasopressin-induced 1 | -2.2 |
| LOC692247 | uncharacterized LOC692247 | -2.2 |
| PSAT1 | phosphoserine aminotransferase 1 | -2.2 |
| MARC1 | mitochondrial amidoxime reducing component 1 | -2.2 |
| ST6GAL1 | ST6 beta-galactosamide alpha-2,6-sialyltranferase 1 | -2.2 |
| VMO1 | vitelline membrane outer layer 1 homolog (chicken) | -2.2 |
| TDRD6 | tudor domain containing 6 | -2.2 |
| GLUD2 | glutamate dehydrogenase 2 | -2.2 |
| STARD5 | StAR-related lipid transfer (START) domain containing 5 | -2.2 |
| FAT4 | FAT tumor suppressor homolog 4 (Drosophila) | -2.2 |
| FAH | fumarylacetoacetate hydrolase (fumarylacetoacetase) | -2.2 |
| TMC4 | transmembrane channel-like 4 | -2.3 |
| ADAMTSL2 | ADAMTS-like 2 | -2.3 |
| DGAT2 | diacylglycerol O-acyltransferase 2 | -2.3 |
| RNASE4 | ribonuclease, RNase A family, 4 | -2.3 |
| GABARAPL1 | GABA(A) receptor-associated protein like 1 | -2.3 |
| IGKV1-5 | immunoglobulin kappa variable 1-5 | -2.3 |
| BAI3 | brain-specific angiogenesis inhibitor 3 | -2.3 |
| GLUD1 | glutamate dehydrogenase 1 | -2.3 |
| CAT | catalase | -2.3 |
| FAHD2A | fumarylacetoacetate hydrolase domain containing 2A | -2.3 |
| LOC100128252 | uncharacterized LOC100128252 | -2.3 |
| QDPR | quinoid dihydropteridine reductase | -2.3 |
| SUCLG2 | succinate-CoA ligase, GDP-forming, beta subunit | -2.3 |
| FCRL3 | Fc receptor-like 3 | -2.3 |
| FAM46C | family with sequence similarity 46, member C | -2.3 |
| F3 | coagulation factor III (thromboplastin, tissue factor) | -2.3 |
| SPINT2 | serine peptidase inhibitor, Kunitz type, 2 | -2.3 |
| PTP4A1 | protein tyrosine phosphatase type IVA, member 1 | -2.4 |
| PXMP2 | peroxisomal membrane protein 2, 22kDa | -2.4 |
| FAM176A | family with sequence similarity 176, member A | -2.4 |
| GPRASP1 | G protein-coupled receptor associated sorting protein 1 | -2.4 |
| ARAP2 | ArfGAP with RhoGAP domain, ankyrin repeat and PH domain 2 | -2.4 |
| CREM | cAMP responsive element modulator | -2.4 |
| mir-214 | microRNA 214 | -2.4 |
| ORM1 | orosomucoid 1 | -2.4 |
| C2orf40 | chromosome 2 open reading frame 40 | -2.4 |
| DMD | dystrophin | -2.4 |
| ALDH2 | aldehyde dehydrogenase 2 family (mitochondrial) | -2.4 |
| CFD | complement factor D (adipsin) | -2.4 |
| KLF9 | Kruppel-like factor 9 | -2.4 |
| KLRC4-KLRK1/KLRK1 | killer cell lectin-like receptor subfamily K, member 1 | -2.4 |
| MFAP4 | microfibrillar-associated protein 4 | -2.4 |
| TNFRSF17 | tumor necrosis factor receptor superfamily, member 17 | -2.4 |
| ACAT1 | acetyl-CoA acetyltransferase 1 | -2.4 |
| IL18RAP | interleukin 18 receptor accessory protein | -2.5 |
| CBS | cystathionine-beta-synthase | -2.5 |
| MTHFD1 | methylenetetrahydrofolate dehydrogenase (NADP+ dependent) 1, methenyltetrahydrofolate cyclohydrolase, formyltetrahydrofolate synthetase | -2.5 |
| TMEM200C | transmembrane protein 200C | -2.5 |
| CDKN1C | cyclin-dependent kinase inhibitor 1C (p57, Kip2) | -2.5 |
| CD302 | CD302 molecule | -2.5 |
| PAH | phenylalanine hydroxylase | -2.5 |
| ART4 | ADP-ribosyltransferase 4 (Dombrock blood group) | -2.5 |
| KCNJ8 | potassium inwardly-rectifying channel, subfamily J, member 8 | -2.5 |
| SARDH | sarcosine dehydrogenase | -2.5 |
| TEK | TEK tyrosine kinase, endothelial | -2.5 |
| CR1 | complement component (3b/4b) receptor 1 (Knops blood group) | -2.5 |
| CCL4 | chemokine (C-C motif) ligand 4 | -2.5 |
| BCKDHB | branched chain keto acid dehydrogenase E1, beta polypeptide | -2.5 |
| IGLL1/IGLL5 | immunoglobulin lambda-like polypeptide 1 | -2.5 |
| FRMD6 | FERM domain containing 6 | -2.6 |
| GJB2 | gap junction protein, beta 2, 26kDa | -2.6 |
| ANKRD36BP2 | ankyrin repeat domain 36B pseudogene 2 | -2.6 |
| MMRN1 | multimerin 1 | -2.6 |
| RGS4 | regulator of G-protein signaling 4 | -2.6 |
| KCNMA1 | potassium large conductance calcium-activated channel, subfamily M, alpha member 1 | -2.6 |
| GRAMD4 | GRAM domain containing 4 | -2.6 |
| BHLHE40 | basic helix-loop-helix family, member e40 | -2.6 |
| KLHL15 | kelch-like 15 (Drosophila) | -2.6 |
| DBH | dopamine beta-hydroxylase (dopamine beta-monooxygenase) | -2.7 |
| SLC2A9 | solute carrier family 2 (facilitated glucose transporter), member 9 | -2.7 |
| EPHA2 | EPH receptor A2 | -2.7 |
| NTS | neurotensin | -2.7 |
| CCL14 | chemokine (C-C motif) ligand 14 | -2.7 |
| VTN | vitronectin | -2.7 |
| BTG2 | BTG family, member 2 | -2.7 |
| TCF21 | transcription factor 21 | -2.7 |
| HAS2 | hyaluronan synthase 2 | -2.7 |
| SERPINA6 | serpin peptidase inhibitor, clade A (alpha-1 antiproteinase, antitrypsin), member 6 | -2.7 |
| CD8A | CD8a molecule | -2.7 |
| PEMT | phosphatidylethanolamine N-methyltransferase | -2.7 |
| CCDC3 | coiled-coil domain containing 3 | -2.8 |
| SMAD9 | SMAD family member 9 | -2.8 |
| THBD | thrombomodulin | -2.8 |
| SCD5 | stearoyl-CoA desaturase 5 | -2.8 |
| RND1 | Rho family GTPase 1 | -2.8 |
| IL33 | interleukin 33 | -2.8 |
| CCDC71L | coiled-coil domain containing 71-like | -2.8 |
| FMO2 | flavin containing monooxygenase 2 (non-functional) | -2.8 |
| IGLV3-21 | immunoglobulin lambda variable 3-21 | -2.9 |
| PFKFB3 | 6-phosphofructo-2-kinase/fructose-2,6-biphosphatase 3 | -2.9 |
| DLL1 | delta-like 1 (Drosophila) | -2.9 |
| CPED1 | cadherin-like and PC-esterase domain containing 1 | -2.9 |
| SGK1 | serum/glucocorticoid regulated kinase 1 | -2.9 |
| NR4A3 | nuclear receptor subfamily 4, group A, member 3 | -2.9 |
| ASL | argininosuccinate lyase | -2.9 |
| PLCXD3 | phosphatidylinositol-specific phospholipase C, X domain containing 3 | -2.9 |
| CD274 | CD274 molecule | -2.9 |
| TSLP | thymic stromal lymphopoietin | -2.9 |
| PTN | pleiotrophin | -3.0 |
| SC5DL | sterol-C5-desaturase (ERG3 delta-5-desaturase homolog, S. cerevisiae)-like | -3.0 |
| MIR22HG | MIR22 host gene (non-protein coding) | -3.0 |
| CHRDL1 | chordin-like 1 | -3.0 |
| IGFBP1 | insulin-like growth factor binding protein 1 | -3.0 |
| DUSP2 | dual specificity phosphatase 2 | -3.0 |
| F13B | coagulation factor XIII, B polypeptide | -3.0 |
| SLC16A2 | solute carrier family 16, member 2 (thyroid hormone transporter) | -3.0 |
| PHLDA1 | pleckstrin homology-like domain, family A, member 1 | -3.0 |
| GATA6 | GATA binding protein 6 | -3.1 |
| KLF6 | Kruppel-like factor 6 | -3.1 |
| RGN | regucalcin (senescence marker protein-30) | -3.1 |
| TARP | TCR gamma alternate reading frame protein | -3.2 |
| MS4A1 | membrane-spanning 4-domains, subfamily A, member 1 | -3.2 |
| IGLL3P | immunoglobulin lambda-like polypeptide 3, pseudogene | -3.2 |
| GZMK | granzyme K (granzyme 3; tryptase II) | -3.2 |
| FEZ1 | fasciculation and elongation protein zeta 1 (zygin I) | -3.3 |
| ADAMTSL3 | ADAMTS-like 3 | -3.3 |
| C5orf4 | chromosome 5 open reading frame 4 | -3.3 |
| AGTR1 | angiotensin II receptor, type 1 | -3.3 |
| SAA2-SAA4/SAA4 | serum amyloid A4, constitutive | -3.3 |
| PCDH9 | protocadherin 9 | -3.4 |
| EDNRB | endothelin receptor type B | -3.4 |
| GPR171 | G protein-coupled receptor 171 | -3.4 |
| JUN | jun proto-oncogene | -3.4 |
| NFIL3 | nuclear factor, interleukin 3 regulated | -3.5 |
| ADCY1 | adenylate cyclase 1 (brain) | -3.5 |
| EGR2 | early growth response 2 | -3.5 |
| FGB | fibrinogen beta chain | -3.5 |
| EPHX2 | epoxide hydrolase 2, cytoplasmic | -3.6 |
| CH25H | cholesterol 25-hydroxylase | -3.7 |
| SYNPO2 | synaptopodin 2 | -3.7 |
| LDLR | low density lipoprotein receptor | -3.7 |
| POU2AF1 | POU class 2 associating factor 1 | -3.7 |
| CDH19 | cadherin 19, type 2 | -3.7 |
| ANGPTL1 | angiopoietin-like 1 | -3.8 |
| SOCS3 | suppressor of cytokine signaling 3 | -3.8 |
| CYP4F3 | cytochrome P450, family 4, subfamily F, polypeptide 3 | -3.9 |
| INMT | indolethylamine N-methyltransferase | -3.9 |
| HBA1/HBA2 | hemoglobin, alpha 1 | -3.9 |
| DUSP5 | dual specificity phosphatase 5 | -3.9 |
| NNMT | nicotinamide N-methyltransferase | -3.9 |
| ZFPM2 | zinc finger protein, multitype 2 | -4.0 |
| ABI3BP | ABI family, member 3 (NESH) binding protein | -4.0 |
| TIMD4 | T-cell immunoglobulin and mucin domain containing 4 | -4.0 |
| CEBPD | CCAAT/enhancer binding protein (C/EBP), delta | -4.0 |
| CD69 | CD69 molecule | -4.0 |
| CRISPLD2 | cysteine-rich secretory protein LCCL domain containing 2 | -4.1 |
| NAT8 | N-acetyltransferase 8 (GCN5-related, putative) | -4.1 |
| GPLD1 | glycosylphosphatidylinositol specific phospholipase D1 | -4.2 |
| CLDN10 | claudin 10 | -4.2 |
| AGXT2L1 | alanine-glyoxylate aminotransferase 2-like 1 | -4.2 |
| LYVE1 | lymphatic vessel endothelial hyaluronan receptor 1 | -4.3 |
| AKAP12 | A kinase (PRKA) anchor protein 12 | -4.3 |
| FNDC5 | fibronectin type III domain containing 5 | -4.3 |
| ADAMTS1 | ADAM metallopeptidase with thrombospondin type 1 motif, 1 | -4.4 |
| ATF3 | activating transcription factor 3 | -4.5 |
| CLIC6 | chloride intracellular channel 6 | -4.5 |
| HBB | hemoglobin, beta | -4.6 |
| CFTR | cystic fibrosis transmembrane conductance regulator (ATP-binding cassette sub-family C, member 7) | -4.6 |
| TFPI2 | tissue factor pathway inhibitor 2 | -4.6 |
| C11orf96 | chromosome 11 open reading frame 96 | -4.6 |
| GABRP | gamma-aminobutyric acid (GABA) A receptor, pi | -4.7 |
| ID4 | inhibitor of DNA binding 4, dominant negative helix-loop-helix protein | -4.9 |
| BBOX1 | butyrobetaine (gamma), 2-oxoglutarate dioxygenase (gamma-butyrobetaine hydroxylase) 1 | -5.0 |
| ITGB8 | integrin, beta 8 | -5.1 |
| HRG | histidine-rich glycoprotein | -5.3 |
| FETUB | fetuin B | -5.4 |
| A1BG | alpha-1-B glycoprotein | -5.8 |
| HPGD | hydroxyprostaglandin dehydrogenase 15-(NAD) | -5.8 |
| HPX | hemopexin | -5.9 |
| NR4A2 | nuclear receptor subfamily 4, group A, member 2 | -6.0 |
| DPT | dermatopontin | -6.2 |
| IL13RA2 | interleukin 13 receptor, alpha 2 | -6.3 |
| AGXT | alanine-glyoxylate aminotransferase | -6.4 |
| SRPX | sushi-repeat containing protein, X-linked | -6.5 |
| HSD17B2 | hydroxysteroid (17-beta) dehydrogenase 2 | -6.9 |
| PTGS2 | prostaglandin-endoperoxide synthase 2 (prostaglandin G/H synthase and cyclooxygenase) | -7.3 |
| IGL@ | immunoglobulin lambda locus | -7.4 |
| IGHM | immunoglobulin heavy constant mu | -7.7 |
| ADH1A | alcohol dehydrogenase 1A (class I), alpha polypeptide | -11.0 |
| C7 | complement component 7 | -17.5 |
