# Supplementary material for: Viral expression and molecular profiling in liver tissue versus microdissected hepatocytes in hepatitis B virus - associated hepatocellular carcinoma
Source: J Transl Med. 2014 Aug 21;12:230. doi: 10.1186/s12967-014-0230-1 (PMC4142136; doi:10.1186/s12967-014-0230-1)
Supplement: Additional file 7: Table S6. — Differentially Expressed Genes Unique to Microdissected Hepatocytes. [file 12967_2014_230_MOESM7_ESM.docx]

| **Table S6.** Differentially Expressed Genes Unique to Microdissected Hepatocytes | | |
| --- | --- | --- |
| Gene Symbol | Gene Title | Fold Change |
| REG3A | regenerating islet-derived 3 alpha | 24.1 |
| MAGEA3/MAGEA6 | melanoma antigen family A, 6 | 12.1 |
| SPARCL1 | SPARC-like 1 (hevin) | 7.4 |
| LINC00348 | long intergenic non-protein coding RNA 348 | 6.4 |
| B3GNT5 | UDP-GlcNAc:betaGal beta-1,3-N-acetylglucosaminyltransferase 5 | 6.3 |
| NUF2 | NUF2, NDC80 kinetochore complex component, homolog (S. cerevisiae) | 5.8 |
| HSPB8 | heat shock 22kDa protein 8 | 5.5 |
| DKK3 | dickkopf 3 homolog (Xenopus laevis) | 5.5 |
| LY96 | lymphocyte antigen 96 | 5.4 |
| HOXA3 | homeobox A3 | 5.2 |
| LPCAT2 | lysophosphatidylcholine acyltransferase 2 | 5.1 |
| CENPA | centromere protein A | 5.0 |
| CEP55 | centrosomal protein 55kDa | 4.9 |
| TSPAN5 | tetraspanin 5 | 4.8 |
| LMOD1 | leiomodin 1 (smooth muscle) | 4.7 |
| HOXA13 | homeobox A13 | 4.6 |
| MYEF2 | myelin expression factor 2 | 4.6 |
| SLC2A5 | solute carrier family 2 (facilitated glucose/fructose transporter), member 5 | 4.6 |
| MCTP1 | multiple C2 domains, transmembrane 1 | 4.5 |
| TTK | TTK protein kinase | 4.5 |
| CRTAP | cartilage associated protein | 4.4 |
| TRIP13 | thyroid hormone receptor interactor 13 | 4.2 |
| GOLM1 | golgi membrane protein 1 | 4.1 |
| LOC100132891 | uncharacterized LOC100132891 | 4.1 |
| SMYD3 | SET and MYND domain containing 3 | 3.9 |
| GBAP1 | glucosidase, beta, acid pseudogene 1 | 3.9 |
| VNN2 | vanin 2 | 3.8 |
| HSPB1 | heat shock 27kDa protein 1 | 3.8 |
| TMTC1 | transmembrane and tetratricopeptide repeat containing 1 | 3.8 |
| FRMD3 | FERM domain containing 3 | 3.8 |
| SMOC2 | SPARC related modular calcium binding 2 | 3.8 |
| ACSL6 | acyl-CoA synthetase long-chain family member 6 | 3.7 |
| DEPDC1B | DEP domain containing 1B | 3.7 |
| FAM169A | family with sequence similarity 169, member A | 3.7 |
| LGALS8 | lectin, galactoside-binding, soluble, 8 | 3.7 |
| BAG2 | BCL2-associated athanogene 2 | 3.6 |
| STAU2 | staufen, RNA binding protein, homolog 2 (Drosophila) | 3.6 |
| ASF1A | ASF1 anti-silencing function 1 homolog A (S. cerevisiae) | 3.5 |
| MSH2 | mutS homolog 2, colon cancer, nonpolyposis type 1 (E. coli) | 3.5 |
| MCAM | melanoma cell adhesion molecule | 3.5 |
| E2F8 | E2F transcription factor 8 | 3.5 |
| ANGPT1 | angiopoietin 1 | 3.5 |
| ZSWIM5 | zinc finger, SWIM-type containing 5 | 3.5 |
| HSP90AB1 | heat shock protein 90kDa alpha (cytosolic), class B member 1 | 3.4 |
| MARCKS | myristoylated alanine-rich protein kinase C substrate | 3.4 |
| CD200 | CD200 molecule | 3.4 |
| CLIC5 | chloride intracellular channel 5 | 3.4 |
| STK39 | serine threonine kinase 39 | 3.4 |
| ANKRD29 | ankyrin repeat domain 29 | 3.4 |
| SWAP70 | SWAP switching B-cell complex 70kDa subunit | 3.4 |
| LOC100288911 | uncharacterized LOC100288911 | 3.4 |
| TBCE | tubulin folding cofactor E | 3.3 |
| LOC541471 | uncharacterized LOC541471 | 3.3 |
| MPPED2 | metallophosphoesterase domain containing 2 | 3.3 |
| SCRN1 | secernin 1 | 3.3 |
| RBP7 | retinol binding protein 7, cellular | 3.2 |
| MPP7 | membrane protein, palmitoylated 7 (MAGUK p55 subfamily member 7) | 3.2 |
| B3GALNT1 | beta-1,3-N-acetylgalactosaminyltransferase 1 (globoside blood group) | 3.2 |
| ATP1B3 | ATPase, Na+/K+ transporting, beta 3 polypeptide | 3.2 |
| EIF5A2 | eukaryotic translation initiation factor 5A2 | 3.2 |
| ZNF738 | zinc finger protein 738 | 3.2 |
| EPDR1 | ependymin related protein 1 (zebrafish) | 3.1 |
| ADAM9 | ADAM metallopeptidase domain 9 | 3.1 |
| SLC35G2 | solute carrier family 35, member G2 | 3.1 |
| VWF | von Willebrand factor | 3.1 |
| STIL | SCL/TAL1 interrupting locus | 3.1 |
| SCPEP1 | serine carboxypeptidase 1 | 3.1 |
| LMNA | lamin A/C | 3.1 |
| MRPS23 | mitochondrial ribosomal protein S23 | 3.1 |
| ZC2HC1A | zinc finger, C2HC-type containing 1A | 3.1 |
| MYO5A | myosin VA (heavy chain 12, myoxin) | 3.1 |
| CNIH4 | cornichon homolog 4 (Drosophila) | 3.0 |
| NEU1 | sialidase 1 (lysosomal sialidase) | 3.0 |
| SLC39A10 | solute carrier family 39 (zinc transporter), member 10 | 3.0 |
| GJA1 | gap junction protein, alpha 1, 43kDa | 3.0 |
| RCN2 | reticulocalbin 2, EF-hand calcium binding domain | 3.0 |
| NPL | N-acetylneuraminate pyruvate lyase (dihydrodipicolinate synthase) | 3.0 |
| CLIC1 | chloride intracellular channel 1 | 3.0 |
| ATP6V1C1 | ATPase, H+ transporting, lysosomal 42kDa, V1 subunit C1 | 3.0 |
| S100A11 | S100 calcium binding protein A11 | 3.0 |
| TMEM106C | transmembrane protein 106C | 2.9 |
| ZNF323 | zinc finger protein 323 | 2.9 |
| PTPN14 | protein tyrosine phosphatase, non-receptor type 14 | 2.9 |
| DCK | deoxycytidine kinase | 2.9 |
| AIDA | axin interactor, dorsalization associated | 2.9 |
| GNS | glucosamine (N-acetyl)-6-sulfatase | 2.9 |
| MANEAL | mannosidase, endo-alpha-like | 2.9 |
| CTSA | cathepsin A | 2.9 |
| COL4A1 | collagen, type IV, alpha 1 | 2.9 |
| TRPS1 | trichorhinophalangeal syndrome I | 2.9 |
| RNF157 | ring finger protein 157 | 2.9 |
| TRAF5 | TNF receptor-associated factor 5 | 2.9 |
| PALLD | palladin, cytoskeletal associated protein | 2.9 |
| ITGAM | integrin, alpha M (complement component 3 receptor 3 subunit) | 2.8 |
| SPAG4 | sperm associated antigen 4 | 2.8 |
| RFC4 | replication factor C (activator 1) 4, 37kDa | 2.8 |
| PEA15 | phosphoprotein enriched in astrocytes 15 | 2.8 |
| CD58 | CD58 molecule | 2.8 |
| ALDOA | aldolase A, fructose-bisphosphate | 2.8 |
| BAMBI | BMP and activin membrane-bound inhibitor homolog (Xenopus laevis) | 2.8 |
| ILF2 | interleukin enhancer binding factor 2, 45kDa | 2.8 |
| FAM54A | family with sequence similarity 54, member A | 2.8 |
| SEMA3G | sema domain, immunoglobulin domain (Ig), short basic domain, secreted, (semaphorin) 3G | 2.8 |
| PPP1R2 | protein phosphatase 1, regulatory (inhibitor) subunit 2 | 2.8 |
| FRZB | frizzled-related protein | 2.8 |
| SYT1 | synaptotagmin I | 2.8 |
| PRIM1 | primase, DNA, polypeptide 1 (49kDa) | 2.8 |
| ZEB1-AS1 | ZEB1 antisense RNA 1 | 2.8 |
| COL4A2 | collagen, type IV, alpha 2 | 2.8 |
| FAT1 | FAT tumor suppressor homolog 1 (Drosophila) | 2.7 |
| SKAP2 | src kinase associated phosphoprotein 2 | 2.7 |
| RDBP | RD RNA binding protein | 2.7 |
| TPM2 | tropomyosin 2 (beta) | 2.7 |
| ACTN2 | actinin, alpha 2 | 2.7 |
| CACYBP | calcyclin binding protein | 2.7 |
| GGH | gamma-glutamyl hydrolase (conjugase, folylpolygammaglutamyl hydrolase) | 2.7 |
| LINC00622 | long intergenic non-protein coding RNA 622 | 2.7 |
| CDK5 | cyclin-dependent kinase 5 | 2.7 |
| SOCS5 | suppressor of cytokine signaling 5 | 2.7 |
| SGCE | sarcoglycan, epsilon | 2.7 |
| NLRP1 | NLR family, pyrin domain containing 1 | 2.7 |
| FAM198B | family with sequence similarity 198, member B | 2.7 |
| PRCC | papillary renal cell carcinoma (translocation-associated) | 2.7 |
| LOX | lysyl oxidase | 2.6 |
| BTG3 | BTG family, member 3 | 2.6 |
| MFSD6 | major facilitator superfamily domain containing 6 | 2.6 |
| ARPC5 | actin related protein 2/3 complex, subunit 5, 16kDa | 2.6 |
| ASRGL1 | asparaginase like 1 | 2.6 |
| TUBA1B | tubulin, alpha 1b | 2.6 |
| HN1 | hematological and neurological expressed 1 | 2.6 |
| TMEM98 | transmembrane protein 98 | 2.6 |
| WDR67 | WD repeat domain 67 | 2.6 |
| EPS8L3 | EPS8-like 3 | 2.6 |
| ENO1 | enolase 1, (alpha) | 2.6 |
| TMEM48 | transmembrane protein 48 | 2.6 |
| MPZL1 | myelin protein zero-like 1 | 2.6 |
| PANX2 | pannexin 2 | 2.6 |
| IMPAD1 | inositol monophosphatase domain containing 1 | 2.6 |
| LRP12 | low density lipoprotein receptor-related protein 12 | 2.6 |
| PHB | prohibitin | 2.6 |
| ZNF107 | zinc finger protein 107 | 2.6 |
| MEOX2 | mesenchyme homeobox 2 | 2.6 |
| RAP2A | RAP2A, member of RAS oncogene family | 2.6 |
| AIF1L | allograft inflammatory factor 1-like | 2.6 |
| IER5 | immediate early response 5 | 2.6 |
| RIT1 | Ras-like without CAAX 1 | 2.6 |
| ANXA2P2 | annexin A2 pseudogene 2 | 2.6 |
| NME1 | NME/NM23 nucleoside diphosphate kinase 1 | 2.5 |
| MIF | macrophage migration inhibitory factor (glycosylation-inhibiting factor) | 2.5 |
| FAM129A | family with sequence similarity 129, member A | 2.5 |
| CCT3 | chaperonin containing TCP1, subunit 3 (gamma) | 2.5 |
| NETO2 | neuropilin (NRP) and tolloid (TLL)-like 2 | 2.5 |
| SPATA5 | spermatogenesis associated 5 | 2.5 |
| PTPRG | protein tyrosine phosphatase, receptor type, G | 2.5 |
| TCEB1 | transcription elongation factor B (SIII), polypeptide 1 (15kDa, elongin C) | 2.5 |
| RRP15 | ribosomal RNA processing 15 homolog (S. cerevisiae) | 2.5 |
| HMGB2 | high mobility group box 2 | 2.5 |
| C7orf23 | chromosome 7 open reading frame 23 | 2.5 |
| RFX5 | regulatory factor X, 5 (influences HLA class II expression) | 2.5 |
| ABCC1 | ATP-binding cassette, sub-family C (CFTR/MRP), member 1 | 2.5 |
| DDOST | dolichyl-diphosphooligosaccharide--protein glycosyltransferase | 2.5 |
| STC1 | stanniocalcin 1 | 2.5 |
| UBE2C | ubiquitin-conjugating enzyme E2C | 2.5 |
| SNN | stannin | 2.5 |
| CKLF | chemokine-like factor | 2.5 |
| TTLL7 | tubulin tyrosine ligase-like family, member 7 | 2.5 |
| GALNT10 | UDP-N-acetyl-alpha-D-galactosamine:polypeptide N-acetylgalactosaminyltransferase 10 (GalNAc-T10) | 2.5 |
| DPCD | deleted in primary ciliary dyskinesia homolog (mouse) | 2.5 |
| SLC1A3 | solute carrier family 1 (glial high affinity glutamate transporter), member 3 | 2.5 |
| ASAP1 | ArfGAP with SH3 domain, ankyrin repeat and PH domain 1 | 2.5 |
| HIST1H2AG | histone cluster 1, H2ag | 2.5 |
| CSTB | cystatin B (stefin B) | 2.5 |
| CAMSAP2 | calmodulin regulated spectrin-associated protein family, member 2 | 2.5 |
| TUBA1C | tubulin, alpha 1c | 2.5 |
| PPT1 | palmitoyl-protein thioesterase 1 | 2.5 |
| FKBP11 | FK506 binding protein 11, 19 kDa | 2.5 |
| LOC284513 | uncharacterized LOC284513 | 2.5 |
| SORT1 | sortilin 1 | 2.4 |
| TAGLN2 | transgelin 2 | 2.4 |
| VWA5A | von Willebrand factor A domain containing 5A | 2.4 |
| TMOD2 | tropomodulin 2 (neuronal) | 2.4 |
| SMC4 | structural maintenance of chromosomes 4 | 2.4 |
| FAM217B | family with sequence similarity 217, member B | 2.4 |
| MCM7 | minichromosome maintenance complex component 7 | 2.4 |
| SPTBN1 | spectrin, beta, non-erythrocytic 1 | 2.4 |
| ELK3 | ELK3, ETS-domain protein (SRF accessory protein 2) | 2.4 |
| CEP68 | centrosomal protein 68kDa | 2.4 |
| PECAM1 | platelet/endothelial cell adhesion molecule 1 | 2.4 |
| PIGF | phosphatidylinositol glycan anchor biosynthesis, class F | 2.4 |
| TACC1 | transforming, acidic coiled-coil containing protein 1 | 2.4 |
| IFT81 | intraflagellar transport 81 homolog (Chlamydomonas) | 2.4 |
| MYBL1 | v-myb myeloblastosis viral oncogene homolog (avian)-like 1 | 2.4 |
| PRTFDC1 | phosphoribosyl transferase domain containing 1 | 2.4 |
| FAM13C | family with sequence similarity 13, member C | 2.4 |
| C1orf198 | chromosome 1 open reading frame 198 | 2.4 |
| TEAD2 | TEA domain family member 2 | 2.4 |
| SERF1A/SERF1B | small EDRK-rich factor 1A (telomeric) | 2.4 |
| UTP23 | UTP23, small subunit (SSU) processome component, homolog (yeast) | 2.4 |
| CD93 | CD93 molecule | 2.4 |
| RAN | RAN, member RAS oncogene family | 2.4 |
| LPGAT1 | lysophosphatidylglycerol acyltransferase 1 | 2.4 |
| ANP32E | acidic (leucine-rich) nuclear phosphoprotein 32 family, member E | 2.4 |
| CANX | calnexin | 2.4 |
| GAPDH | glyceraldehyde-3-phosphate dehydrogenase | 2.4 |
| AFAP1L1 | actin filament associated protein 1-like 1 | 2.4 |
| CCDC77 | coiled-coil domain containing 77 | 2.4 |
| DISC1 | disrupted in schizophrenia 1 | 2.4 |
| SAP30 | Sin3A-associated protein, 30kDa | 2.3 |
| TMEM144 | transmembrane protein 144 | 2.3 |
| ZBTB26 | zinc finger and BTB domain containing 26 | 2.3 |
| CFL1 | cofilin 1 (non-muscle) | 2.3 |
| PDK1 | pyruvate dehydrogenase kinase, isozyme 1 | 2.3 |
| PPP1R16A | protein phosphatase 1, regulatory subunit 16A | 2.3 |
| C3orf32 | chromosome 3 open reading frame 32 | 2.3 |
| KCNE3 | potassium voltage-gated channel, Isk-related family, member 3 | 2.3 |
| ADA | adenosine deaminase | 2.3 |
| IGDCC4 | immunoglobulin superfamily, DCC subclass, member 4 | 2.3 |
| RHOA | ras homolog family member A | 2.3 |
| C2orf76 | chromosome 2 open reading frame 76 | 2.3 |
| SLC25A43 | solute carrier family 25, member 43 | 2.3 |
| LPCAT1 | lysophosphatidylcholine acyltransferase 1 | 2.3 |
| DYNLL1 | dynein, light chain, LC8-type 1 | 2.3 |
| CBX4 | chromobox homolog 4 | 2.3 |
| IPO11 | importin 11 | 2.3 |
| DR1 | down-regulator of transcription 1, TBP-binding (negative cofactor 2) | 2.3 |
| BBS12 | Bardet-Biedl syndrome 12 | 2.3 |
| ARF1 | ADP-ribosylation factor 1 | 2.3 |
| YWHAB | tyrosine 3-monooxygenase/tryptophan 5-monooxygenase activation protein, beta polypeptide | 2.3 |
| BROX | BRO1 domain and CAAX motif containing | 2.3 |
| GOLPH3L | golgi phosphoprotein 3-like | 2.2 |
| GLA | galactosidase, alpha | 2.2 |
| LOC100505519 | uncharacterized LOC100505519 | 2.2 |
| PRLR | prolactin receptor | 2.2 |
| METTL18 | methyltransferase like 18 | 2.2 |
| C11orf73 | chromosome 11 open reading frame 73 | 2.2 |
| MTMR2 | myotubularin related protein 2 | 2.2 |
| TIPIN | TIMELESS interacting protein | 2.2 |
| OVOS/OVOS2 | ovostatin | 2.2 |
| ADAMTS9 | ADAM metallopeptidase with thrombospondin type 1 motif, 9 | 2.2 |
| MRPS12 | mitochondrial ribosomal protein S12 | 2.2 |
| PTBP3 | polypyrimidine tract binding protein 3 | 2.2 |
| PSME3 | proteasome (prosome, macropain) activator subunit 3 (PA28 gamma; Ki) | 2.2 |
| CCDC90A | coiled-coil domain containing 90A | 2.2 |
| SEPT8 | septin 8 | 2.2 |
| AP3M2 | adaptor-related protein complex 3, mu 2 subunit | 2.2 |
| COMMD8 | COMM domain containing 8 | 2.2 |
| CLN3 | ceroid-lipofuscinosis, neuronal 3 | 2.2 |
| USP46 | ubiquitin specific peptidase 46 | 2.2 |
| DLAT | dihydrolipoamide S-acetyltransferase | 2.2 |
| C8orf59 | chromosome 8 open reading frame 59 | 2.2 |
| ACLY | ATP citrate lyase | 2.2 |
| NAP1L1 | nucleosome assembly protein 1-like 1 | 2.2 |
| TSEN15 | tRNA splicing endonuclease 15 homolog (S. cerevisiae) | 2.2 |
| TOMM20 | translocase of outer mitochondrial membrane 20 homolog (yeast) | 2.2 |
| DSTYK | dual serine/threonine and tyrosine protein kinase | 2.2 |
| TGFB2 | transforming growth factor, beta 2 | 2.2 |
| MAP4K4 | mitogen-activated protein kinase kinase kinase kinase 4 | 2.2 |
| POLR3K | polymerase (RNA) III (DNA directed) polypeptide K, 12.3 kDa | 2.2 |
| LOC642852 | uncharacterized LOC642852 | 2.2 |
| SMAD2 | SMAD family member 2 | 2.2 |
| UXS1 | UDP-glucuronate decarboxylase 1 | 2.2 |
| KBTBD2 | kelch repeat and BTB (POZ) domain containing 2 | 2.2 |
| KDM5B | lysine (K)-specific demethylase 5B | 2.2 |
| CASK | calcium/calmodulin-dependent serine protein kinase (MAGUK family) | 2.2 |
| RHOC | ras homolog family member C | 2.2 |
| ARPC1B | actin related protein 2/3 complex, subunit 1B, 41kDa | 2.2 |
| LAMB3 | laminin, beta 3 | 2.2 |
| LYPLAL1 | lysophospholipase-like 1 | 2.2 |
| ATP8B2 | ATPase, aminophospholipid transporter, class I, type 8B, member 2 | 2.2 |
| AACS | acetoacetyl-CoA synthetase | 2.1 |
| FLT1 | fms-related tyrosine kinase 1 (vascular endothelial growth factor/vascular permeability factor receptor) | 2.1 |
| GPR176 | G protein-coupled receptor 176 | 2.1 |
| WASF1 | WAS protein family, member 1 | 2.1 |
| HNRNPU | heterogeneous nuclear ribonucleoprotein U (scaffold attachment factor A) | 2.1 |
| AIFM2 | apoptosis-inducing factor, mitochondrion-associated, 2 | 2.1 |
| SMARCC1 | SWI/SNF related, matrix associated, actin dependent regulator of chromatin, subfamily c, member 1 | 2.1 |
| LOC100127983 | uncharacterized LOC100127983 | 2.1 |
| BBS7 | Bardet-Biedl syndrome 7 | 2.1 |
| ZNF174 | zinc finger protein 174 | 2.1 |
| ARPC1A | actin related protein 2/3 complex, subunit 1A, 41kDa | 2.1 |
| ZNF238 | zinc finger protein 238 | 2.1 |
| VARS | valyl-tRNA synthetase | 2.1 |
| HEY1 | hairy/enhancer-of-split related with YRPW motif 1 | 2.1 |
| GPD2 | glycerol-3-phosphate dehydrogenase 2 (mitochondrial) | 2.1 |
| GPATCH4 | G patch domain containing 4 | 2.1 |
| TMA16 | translation machinery associated 16 homolog (S. cerevisiae) | 2.1 |
| SFR1 | SWI5-dependent recombination repair 1 | 2.1 |
| RALA | v-ral simian leukemia viral oncogene homolog A (ras related) | 2.1 |
| GRN | granulin | 2.1 |
| IMPDH2 | IMP (inosine 5'-monophosphate) dehydrogenase 2 | 2.1 |
| GOLT1B | golgi transport 1B | 2.1 |
| S100A6 | S100 calcium binding protein A6 | 2.1 |
| ZNF704 | zinc finger protein 704 | 2.1 |
| LIX1L | Lix1 homolog (mouse)-like | 2.1 |
| HMGN4 | high mobility group nucleosomal binding domain 4 | 2.1 |
| PAPSS1 | 3'-phosphoadenosine 5'-phosphosulfate synthase 1 | 2.1 |
| C7orf60 | chromosome 7 open reading frame 60 | 2.1 |
| YEATS4 | YEATS domain containing 4 | 2.1 |
| PDIA6 | protein disulfide isomerase family A, member 6 | 2.1 |
| HEXA | hexosaminidase A (alpha polypeptide) | 2.1 |
| IRF6 | interferon regulatory factor 6 | 2.1 |
| PSMB4 | proteasome (prosome, macropain) subunit, beta type, 4 | 2.1 |
| LOC100996419 | uncharacterized LOC100996419 | 2.1 |
| ZNF521 | zinc finger protein 521 | 2.1 |
| NDE1 | nudE nuclear distribution E homolog 1 (A. nidulans) | 2.1 |
| DCLK1 | doublecortin-like kinase 1 | 2.1 |
| BCAP31 | B-cell receptor-associated protein 31 | 2.1 |
| DNAJC10 | DnaJ (Hsp40) homolog, subfamily C, member 10 | 2.1 |
| MRAS | muscle RAS oncogene homolog | 2.1 |
| SSR2 | signal sequence receptor, beta (translocon-associated protein beta) | 2.1 |
| CDC42SE2 | CDC42 small effector 2 | 2.1 |
| PIK3R3 | phosphoinositide-3-kinase, regulatory subunit 3 (gamma) | 2.1 |
| MAD2L2 | MAD2 mitotic arrest deficient-like 2 (yeast) | 2.1 |
| CYTH3 | cytohesin 3 | 2.1 |
| RAB7L1 | RAB7, member RAS oncogene family-like 1 | 2.1 |
| PRUNE | prune homolog (Drosophila) | 2.1 |
| MIB1 | mindbomb E3 ubiquitin protein ligase 1 | 2.1 |
| PDZD2 | PDZ domain containing 2 | 2.1 |
| GPX1 | glutathione peroxidase 1 | 2.1 |
| FADS1 | fatty acid desaturase 1 | 2.1 |
| LSM4 | LSM4 homolog, U6 small nuclear RNA associated (S. cerevisiae) | 2.1 |
| MITD1 | MIT, microtubule interacting and transport, domain containing 1 | 2.1 |
| RFC3 | replication factor C (activator 1) 3, 38kDa | 2.1 |
| DUS4L | dihydrouridine synthase 4-like (S. cerevisiae) | 2.1 |
| TAF10 | TAF10 RNA polymerase II, TATA box binding protein (TBP)-associated factor, 30kDa | 2.1 |
| DTNBP1 | dystrobrevin binding protein 1 | 2.0 |
| TULP3 | tubby like protein 3 | 2.0 |
| TET1 | tet methylcytosine dioxygenase 1 | 2.0 |
| PRDX1 | peroxiredoxin 1 | 2.0 |
| PLK1S1 | polo-like kinase 1 substrate 1 | 2.0 |
| BBX | bobby sox homolog (Drosophila) | 2.0 |
| EFCAB7 | EF-hand calcium binding domain 7 | 2.0 |
| TUBB3 | tubulin, beta 3 class III | 2.0 |
| AZIN1 | antizyme inhibitor 1 | 2.0 |
| OTUD6B | OTU domain containing 6B | 2.0 |
| MPV17 | MpV17 mitochondrial inner membrane protein | 2.0 |
| HSPA13 | heat shock protein 70kDa family, member 13 | 2.0 |
| RAD21 | RAD21 homolog (S. pombe) | 2.0 |
| ORMDL2 | ORM1-like 2 (S. cerevisiae) | 2.0 |
| GTF2H2 | general transcription factor IIH, polypeptide 2, 44kDa | 2.0 |
| GCKR | glucokinase (hexokinase 4) regulator | -2.0 |
| UBE2D3 | ubiquitin-conjugating enzyme E2D 3 | -2.0 |
| RAB6A | RAB6A, member RAS oncogene family | -2.0 |
| JMJD1C | jumonji domain containing 1C | -2.0 |
| FGA | fibrinogen alpha chain | -2.0 |
| FLNB | filamin B, beta | -2.0 |
| SLC16A1 | solute carrier family 16, member 1 (monocarboxylic acid transporter 1) | -2.0 |
| ZNF542 | zinc finger protein 542 | -2.0 |
| GSDMB | gasdermin B | -2.0 |
| EHBP1 | EH domain binding protein 1 | -2.0 |
| CAMK2B | calcium/calmodulin-dependent protein kinase II beta | -2.0 |
| C21orf91 | chromosome 21 open reading frame 91 | -2.0 |
| NUMB | numb homolog (Drosophila) | -2.1 |
| APOA1 | apolipoprotein A-I | -2.1 |
| AK3 | adenylate kinase 3 | -2.1 |
| PALM3 | paralemmin 3 | -2.1 |
| C10orf116 | chromosome 10 open reading frame 116 | -2.1 |
| SPRY4-IT1 | SPRY4 intronic transcript 1 (non-protein coding) | -2.1 |
| KIAA1456 | KIAA1456 | -2.1 |
| SMAD1 | SMAD family member 1 | -2.1 |
| SPATS2L | spermatogenesis associated, serine-rich 2-like | -2.1 |
| SCAF4 | SR-related CTD-associated factor 4 | -2.1 |
| APOC3 | apolipoprotein C-III | -2.1 |
| OSBP | oxysterol binding protein | -2.1 |
| ZC2HC1C | zinc finger, C2HC-type containing 1C | -2.1 |
| FRMD4A | FERM domain containing 4A | -2.1 |
| THSD4 | thrombospondin, type I, domain containing 4 | -2.1 |
| NMRK1 | nicotinamide riboside kinase 1 | -2.1 |
| CYP4F12 | cytochrome P450, family 4, subfamily F, polypeptide 12 | -2.1 |
| NME5 | NME/NM23 family member 5 | -2.1 |
| MTUS1 | microtubule associated tumor suppressor 1 | -2.1 |
| PDE3B | phosphodiesterase 3B, cGMP-inhibited | -2.1 |
| DNAJC25 | DnaJ (Hsp40) homolog, subfamily C , member 25 | -2.1 |
| ZFP1 | zinc finger protein 1 homolog (mouse) | -2.2 |
| NANOGNB | NANOG neighbor homeobox | -2.2 |
| NAT1 | N-acetyltransferase 1 (arylamine N-acetyltransferase) | -2.2 |
| IAPP | islet amyloid polypeptide | -2.2 |
| SIK3 | SIK family kinase 3 | -2.2 |
| PROX1 | prospero homeobox 1 | -2.2 |
| ANKRD18A | ankyrin repeat domain 18A | -2.2 |
| TMED5 | transmembrane emp24 protein transport domain containing 5 | -2.2 |
| SGK2 | serum/glucocorticoid regulated kinase 2 | -2.2 |
| FITM1 | fat storage-inducing transmembrane protein 1 | -2.2 |
| SEC24D | SEC24 family, member D (S. cerevisiae) | -2.2 |
| B2M | beta-2-microglobulin | -2.2 |
| CMYA5 | cardiomyopathy associated 5 | -2.2 |
| KIAA1217 | KIAA1217 | -2.2 |
| ZCCHC2 | zinc finger, CCHC domain containing 2 | -2.2 |
| MBD4 | methyl-CpG binding domain protein 4 | -2.2 |
| PRO2852 | uncharacterized protein PRO2852 | -2.2 |
| BCL2L11 | BCL2-like 11 (apoptosis facilitator) | -2.2 |
| SLC17A9 | solute carrier family 17, member 9 | -2.2 |
| SPATA13 | spermatogenesis associated 13 | -2.2 |
| PCK2 | phosphoenolpyruvate carboxykinase 2 (mitochondrial) | -2.2 |
| LPP | LIM domain containing preferred translocation partner in lipoma | -2.2 |
| MORF4L2 | mortality factor 4 like 2 | -2.2 |
| WDR1 | WD repeat domain 1 | -2.2 |
| STAT3 | signal transducer and activator of transcription 3 (acute-phase response factor) | -2.2 |
| CFLAR | CASP8 and FADD-like apoptosis regulator | -2.2 |
| ASGR2 | asialoglycoprotein receptor 2 | -2.2 |
| CLN8 | ceroid-lipofuscinosis, neuronal 8 (epilepsy, progressive with mental retardation) | -2.3 |
| INTS6 | integrator complex subunit 6 | -2.3 |
| CAND2 | cullin-associated and neddylation-dissociated 2 (putative) | -2.3 |
| LPAL2 | lipoprotein, Lp(a)-like 2, pseudogene | -2.3 |
| LOC100128508 | PP12100 | -2.3 |
| PELI1 | pellino E3 ubiquitin protein ligase 1 | -2.3 |
| FBXO21 | F-box protein 21 | -2.3 |
| SLC8A1 | solute carrier family 8 (sodium/calcium exchanger), member 1 | -2.3 |
| SCNN1A | sodium channel, non-voltage-gated 1 alpha subunit | -2.3 |
| PPFIBP1 | PTPRF interacting protein, binding protein 1 (liprin beta 1) | -2.3 |
| WNT11 | wingless-type MMTV integration site family, member 11 | -2.3 |
| SLC26A6 | solute carrier family 26, member 6 | -2.3 |
| PLCG2 | phospholipase C, gamma 2 (phosphatidylinositol-specific) | -2.3 |
| C4orf29 | chromosome 4 open reading frame 29 | -2.4 |
| LOC100506974 | uncharacterized LOC100506974 | -2.4 |
| FCGR2B | Fc fragment of IgG, low affinity IIb, receptor (CD32) | -2.4 |
| IRF8 | interferon regulatory factor 8 | -2.4 |
| ATOH7 | atonal homolog 7 (Drosophila) | -2.4 |
| ST3GAL6 | ST3 beta-galactoside alpha-2,3-sialyltransferase 6 | -2.4 |
| ABCA9 | ATP-binding cassette, sub-family A (ABC1), member 9 | -2.5 |
| PNPLA3 | patatin-like phospholipase domain containing 3 | -2.5 |
| PRKRA | protein kinase, interferon-inducible double stranded RNA dependent activator | -2.5 |
| DST | dystonin | -2.5 |
| RHEB | Ras homolog enriched in brain | -2.5 |
| CPT1A | carnitine palmitoyltransferase 1A (liver) | -2.5 |
| TPPP2 | tubulin polymerization-promoting protein family member 2 | -2.5 |
| ARMCX3 | armadillo repeat containing, X-linked 3 | -2.6 |
| NSUN6 | NOP2/Sun domain family, member 6 | -2.6 |
| HES1 | hairy and enhancer of split 1, (Drosophila) | -2.6 |
| SDCBP2 | syndecan binding protein (syntenin) 2 | -2.6 |
| ABCG5 | ATP-binding cassette, sub-family G (WHITE), member 5 | -2.6 |
| CCDC150 | coiled-coil domain containing 150 | -2.6 |
| RALGAPA2 | Ral GTPase activating protein, alpha subunit 2 (catalytic) | -2.7 |
| C8orf42 | chromosome 8 open reading frame 42 | -2.7 |
| PRPF18 | PRP18 pre-mRNA processing factor 18 homolog (S. cerevisiae) | -2.7 |
| MAGI1 | membrane associated guanylate kinase, WW and PDZ domain containing 1 | -2.8 |
| CSAD | cysteine sulfinic acid decarboxylase | -2.8 |
| GNAO1 | guanine nucleotide binding protein (G protein), alpha activating activity polypeptide O | -2.8 |
| TTR | transthyretin | -2.8 |
| PRSS8 | protease, serine, 8 | -2.9 |
| SCG5 | secretogranin V (7B2 protein) | -2.9 |
| PTGIS | prostaglandin I2 (prostacyclin) synthase | -3.0 |
| PHLDB2 | pleckstrin homology-like domain, family B, member 2 | -3.0 |
| AGBL2 | ATP/GTP binding protein-like 2 | -3.0 |
| ZFAND5 | zinc finger, AN1-type domain 5 | -3.0 |
| LGSN | lengsin, lens protein with glutamine synthetase domain | -3.0 |
| PLG | plasminogen | -3.0 |
| LOC283587 | uncharacterized LOC283587 | -3.1 |
| NREP | neuronal regeneration related protein homolog (rat) | -3.1 |
| SPIRE1 | spire homolog 1 (Drosophila) | -3.3 |
| FAM9B | family with sequence similarity 9, member B | -3.3 |
| SCARNA17 | small Cajal body-specific RNA 17 | -3.3 |
| PIK3C2G | phosphatidylinositol-4-phosphate 3-kinase, catalytic subunit type 2 gamma | -3.3 |
| DBH-AS1 | DBH antisense RNA 1 | -3.5 |
| TMEM154 | transmembrane protein 154 | -3.6 |
| MXRA5 | matrix-remodelling associated 5 | -3.6 |
| LONRF2 | LON peptidase N-terminal domain and ring finger 2 | -3.6 |
| WDR72 | WD repeat domain 72 | -3.7 |
| PKHD1 | polycystic kidney and hepatic disease 1 (autosomal recessive) | -3.7 |
| CFHR3 | complement factor H-related 3 | -3.9 |
| SULT1E1 | sulfotransferase family 1E, estrogen-preferring, member 1 | -4.0 |
| SLC1A1 | solute carrier family 1 (neuronal/epithelial high affinity glutamate transporter, system Xag), member 1 | -4.0 |
| GRHL1 | grainyhead-like 1 (Drosophila) | -4.2 |
| LOC284801 | uncharacterized LOC284801 | -4.2 |
| F9 | coagulation factor IX | -4.3 |
| BGN | biglycan | -4.6 |
| GABRB3 | gamma-aminobutyric acid (GABA) A receptor, beta 3 | -4.9 |
| CLRN3 | clarin 3 | -5.8 |
